# Novel approaches to circumvent the devastating effects of pests on sugarcane

**DOI:** 10.1038/s41598-021-91985-8

**Published:** 2021-06-14

**Authors:** Zahida Qamar, Idrees Ahmad Nasir, Mounir G. Abouhaidar, Kathleen L. Hefferon, Abdul Qayyum Rao, Ayesha Latif, Qurban Ali, Saima Anwar, Bushra Rashid, Ahmad Ali Shahid

**Affiliations:** 1 Centre of Excellence in Molecular Biology, University of the Punjab, Lahore, Pakistan; 2 Department of Cell and Systems Biology, University of Toronto, Toronto, ON Canada; 3 Department of Microbiology, Cornell University, Ithaca, NY 14850 USA; 4 Institute of Molecular Biology and Biotechnology, The University of Lahore, Lahore, Pakistan; 5 Pakistan Biomedical Engineering Centre University of Engineering and Technology, New Campus, Lahore, Pakistan

**Keywords:** Biotechnology, Plant biotechnology

## Abstract

Sugarcane (*Saccharum officinarum* L*.*) is a cash crop grown commercially for its higher amounts of sucrose, stored within the mature internodes of the stem. Numerous studies have been done for the resistance development against biotic and abiotic stresses to save the sucrose yields. Quality and yield of sugarcane production is always threatened by the damages of cane borers and weeds. In current study two problems were better addressed through the genetic modification of sugarcane for provision of resistance against insects and weedicide via the expression of two modified cane borer resistant CEMB-*Cry*1Ac (1.8 kb), CEMB-*Cry*2A (1.9 kb) and one glyphosate tolerant CEMB-GTGene (1.4 kb) genes, driven by maize Ubiquitin Promoter and nos terminator. Insect Bio-toxicity assays were carried out for the assessment of *Cry* proteins through mortality percent of shoot borer *Chilo infuscatellus* at 2nd instar larvae stage. During V_0_, V_1_ and V_2_ generations young leaves from the transgenic sugarcane plants were collected at plant age of 20, 40, 60, 80 days and fed to the *Chilo infuscatellus* larvae. Up to 100% mortality of *Chilo infuscatellus* from 80 days old transgenic plants of V_2_ generation indicated that these transgenic plants were highly resistant against shoot borer and the gene expression level is sufficient to provide complete resistance against target pests. Glyphosate spray assay was carried out for complete removal of weeds. In V_1_-generation, 70–76% transgenic sugarcane plants were found tolerant against glyphosate spray (3000 mL/ha) under field conditions. While in V_2_-generation, the replicates of five selected lines 4L/2, 5L/5, 6L/5, L8/4, and L9/6 were found 100% tolerant against 3000 mL/ha glyphosate spray. It is evident from current study that CEMB-GTGene, CEMB-*Cry*1Ac and CEMB-*Cry*2A genes expression in sugarcane variety CPF-246 showed an efficient resistance against cane borers (*Chilo infuscatellus*) and was also highly tolerant against glyphosate spray. The selected transgenic sugarcane lines showed sustainable resistance against cane borer and glyphosate spray can be further exploited at farmer’s field level after fulfilling the biosafety requirements to boost the sugarcane production in the country.

## Introduction

Sugarcane (*Saccharum officinarum* L.) is significantly prominent among the essential food cash crops of the world^1–3^ which has been cultivated in 58 countries around the globe. The projected area of the world under sugarcane cultivation was 26.9 million hectares^4^. This grass of genus *Saccharum* meets 80% of world sugar requirements though most popular sweetener chemically known as sucrose^5,6^. Sucrose deposited in stalk’s internodes^7^. About two billion metric ton stalks are crushed in sugar mills to get the sucrose juice that produces 2/3 world sugar production^8^. Sugarcane is more than just a sugar crop among the cash crops of the world^9,10^. Sugarcane yields a variety of products from fiber to biofuel, chemicals and industrial products such as alcohol, furfural, dextran, chipboard, paper, beverages, confectionery, plastics, paints, synthetics, pharmaceutical products, industrial enzymes, insecticides and detergents^1,11,12^.

In Pakistan, sugarcane has been cultivated on an area of 1.2171 million hectares and contributes 3.4% value added in agriculture sector amounting to nearly 0.7% of the GDP. Pakistan boosted the overall sugarcane production to 73.6 million tons of the commodities^13^. This gives Pakistan a reputation as being one of the top five sugarcane producers across the world. It occupies an important position in the national economy of Pakistan, by employing more than 9 million Pakistanis, significantly helping out driving the export economy of Pakistan^14^ and proving itself a backbone of second largest agroindustry of Pakistan by providing raw material to 84 sugar mills for sugar production^2^. Pakistan exports sugar to the Tajikistan, Afghanistan, and other central Asian countries. Sugar industry is entirely dependent on the availability of sugarcane for sugar production in Pakistan^15^.

Globally, pests along with diseases cause 37% loss to the agriculture production from which 13% is just because of insects^16,17^. Approximately 1300, insect pests attack on sugarcane crop around the world. In Pakistan 61 insect species have been accounted for attacking sugarcane crop^18^. The borers species reported from Pakistan included *Chilo infuscatellus* and *Chilo auricilius* (stem borers) with 15–36% yield loss, *Scirpophaga excerptalis* and *Scirpophaga novella* (top borers) with 10–15% loss, *Emmalocera depressella* (root borer) with 10–20% crop infestation, *Acigona steniella* (Gurdaspur borer) with 20% usual yield loss and *Pyrilla perpusilla* (sugarcane leaf hoper/Walker) with 25% crop loss respectively. Due to 80% leaf infestation and 15–25% yield reduction, *Aleurolobus barodensis* (white fly) is also considered among the most deleterious insects. *Saccharicoccus sacchari* (Mealy bug) and *Cavelerious excavatus* (black bug) are minor pests of sugarcane who attack the crop throughout the year^19^. Extensive and excessive use of insecticides/pesticides is increasing every year to control yield losses from insect’s damage.

*Cry* genes from *Bacillus thuringiensis* (*Bt*.) produces crystalline protein that defend transgenic plants against *Lepidopteran*, *Dipterans* and *Coleopteran*^20–22^. *Bt.* crops now contain *Cry* genes such as *Cry*1Ab, *Cry*1Ac, *Cry*1Ac + *Cry*1F, *Cry*2A, *Cry*1Ac + *Cry*2A significantly efficient in controlling *Lepidopteran* insects^23,24^. The *Bt.* transformed crops were observed with minimum insecticidal sprays throughout the season while non-transgenic plants were treated with five to twelve pesticidal sprays^25^. Glyphosate (N-phosphonomethyl glycine) is a broad spectrum and most effective herbicide to control weeds. Glyphosate usually kills the plants through the blockage of EPSPS^26^ enzyme. Incorporation of this soil bacterium gene produces a glyphosate tolerant form of EPSPS enzyme; its binding affinity for glyphosate confers the increased tolerance against glyphosate in transgenic plants^27–31^. Its distinctive target (EPSPS enzyme), non-selective nature for the plants/weeds, great potential to sink inside the tissues of the plants, and minor toxicity effects to the atmosphere and the human beings has rendered it to its current status^32,33^. The objectives of our study was that by controlling these cane borers and weeds, improved self sufficiency in sugar production is achievable to execute the compelling/demanding requirement of the increasing population for the sweetener utility.

## Materials and methods

### Construction of plant expression vectors

The *CEMB-Cry1Ac*, *CEMB-Cry2A* and *CEMB-GTGene* are patented by the Centre of Excellence in Molecular Biology (CEMB), University of the Punjab Lahore, Pakistan (Patent No. K140649-aroA). It have been confirmed that the experimental research and field studies on sugarcane plants, including the collection of plant material, complied with relevant institutional, national, and international guidelines and legislation with appropriate permissions from Centre of Excellence in Molecular Biology (CEMB), University of the Punjab Lahore, and Ayub Agricultural Research Institute Faisalabad, Pakistan for collection of plant specimens. These genes were constructed with maize Ubiquitin promoter and Nos terminator independently in separate cassettes (Supplementary Figure 1). Chemically synthesized genes were confirmed via restriction digestion as well as through protein expression studies as was done (Rao et al*.*^34^; Qamar et al.^35^).

### Transient expression of transgene (Gus) to determine the efficacy of plasmid constructs

#### Electroporation

Plasmids constructs *pCEMB-SC12* and *pCEMB-SGTG* were transformed into *Agrobacteriurm tumefaciens* competent cells (LBA4404) with Bio-Rad Electroporator (Model 1652078) using standard procedure as done by Puspito et al.^27^. Transformed cells were spread on YEP medium plates with kanamycin (50 mg/L) and incubated at 30 °C for overnight. Positive transformants were confirmed by colony PCR that was performed using gene specific primers.

### Agroinfiltration of tobacco leaves

Agrobacterium-mediated transient transformation of tobacco was conducted on fully expanded leaves attached to 8–10 weeks-old intact plants. Bacterial suspension prepared as reported by Bhaskar et al.^36^ was infiltrated into intercellular spaces of intact leaves using a 1 ml syringe. Tagged *agro*infiltrated leaves were left for 72 h in the greenhouse at 28 °C with 16 h light period.

### Histochemical detection of GUS activity

The agroinfiltrated leaves were sacrificed for histochemical detection of GUS activity that was used as a reporter for expression of transgenes. They were incubated with substrate solution (0.08% w/v 5-bromo-4-chloro-3-indolyl-b-Dglucuronic acid (X-Gluc; sigma) in 100 mM Na2HPO4/KH2PO4 pH 7.0, 0.01% w/v Chloramphenicol, 20% Triton X-100, 20% Methanol) at 37 °C for 16 h. The GUS expression was detected in the form of blue precipitates in agroinfiltrated leaves under fluorescent microscope that were produced as a result of enzyme mediated hydrolysis of X-Gluc.

### Genetic transformation and regeneration of transformants

Four sugarcane cultivars SPF-213, SPF-234, HSF-240 and CPF-246 were received from Agricultural Research Institute (AARI), Faisalabad, Pakistan. Genetic transformation of these lines was done using the biolistic transformation method. Tungsten particles were used as microprojectiles and were prepared as described Qamar et al.^35^. The sugarcane transformation was proceeded by following the protocol described by Nasir et al.^37^.

### Molecular characterization of transgenic lines

GUS assay was performed on young shoots to detect expression of transgene during early events of transgenesis. Subsequently potential transgenic lines (V_0_ as well as V_1_ generation) were further screened for integration and expression of foreign genes by gene specific PCR, dipstick assay, southern blotting and enzyme linked immunosorbent assay (ELISA). After molecular characterization, qualitative assessment of transgenic plants of V_0_, V_1_ and V_2_ generation was performed via biotoxicity assay and Glyphosate spray assay.

### Screening through polymerase chain reaction (PCR)

Genomic DNA was extracted from fresh leaves of transgenic lines using the method described by Edward et al.^38^. After qualitative as well as quantitative assessment of DNA, PCR reactions were set up using *CEMB-CrylAc*, *CEMB-Cry2A* and *CEMB-GTGene* specific primers. DNA from non-transformed plant was included as a negative control while plant expression constructs were included as positive controls. Each PCR reaction was carried out in a total volume of 20 µL having 100 ng of template DNA, 50 pm of each forward and reverse primers, 200 mM dNTPs and 1–2 units of Taq DNA polymerase. Success and specificity of reaction was checked by running 15 µL of each reaction on 1% agarose gel. Primer sequences are given in Supplementary material (Table 1).

**Table 1:** Varietal responses of sugarcane cultivars for tissue culturing.

Sr. no	Varieties	Callus induction (%)	Regenerated callus (%)	Shoot multiplication (%)
1	SPF-213	80	65	80
2	HSF-234	81	80	90
3	CPF-240	68	62	81
4	CPF-246	98	99	100

### Southern blotting

Southern blot analysis was performed on PCR positive transformed lines to confirm stable integration. High quality genomic DNA was isolated from leaf tissues using plant phytopure DNA extraction kit (Amersham) following the manufacturer’s instructions. Approximately 20 µg of genomic DNA from each transgenic line along with control plants was double digested with *KpnI & SacI* for *CEMB-GTGene*, *KpnI* and *HindIII* for *CEMB-Cry1Ac* and *CEMB-Cry2A*. After complete digestion, each digestion mixture was resolved on 1% agarose gel at 15 V for 18 h. and transferred onto the nylon membrane (Hi-bond) using standard capillary transfer^39^. DIG labeled probe of each gene was prepared using Decanucleotide Biotin labeling Kit (Cat # Ko561) according to the manufacturer’s instructions and then hybridization and detection was done using Cat# K0562.

### Dipstick assay

Total protein was extracted from fresh leaves of transgenic plants and then quantified as described by Qamar et al.^35^. The GTGene*, Cry1Ac and Cry2A* expression was detected with EnviroLogix^®^ dipstick kits (Cat # AS011LSS; Cat # AS003 # CTLSS and Cat # AS005 LSS) following the manufacturer’s instructions. Dipsticks were coated with monoclonal antibodies IgG of purified *EPSPS, Cry1Ac and Cry2A respectively.* The antibody coated dipsticks were dipped in transgenic plants protein samples and incubated at room temperature for 15 min.

### Enzyme linked immunosorbant assay (ELISA)

Protein expressions of *CEMB-Cry1Ac*, *CEMB-Cry2A,* and *CEMB-GTGene* were quantified in sugarcane positive lines through ELISA. Total crude protein from fresh leaves was used in ELISA. Total crude protein was extracted using protein extraction buffer (10% Glycerol, 40 mM EDTA (pH 7.5), 100 mM NaCl, 10 mM Tris, 100 mM NH4Cl, 20 mM DTT, 2 mM PMSF) and then quantified by Bradford assay^40^. ELISA was performed according to the instruction manual of Envirologix Kit (Portland USA; Cat # 051) and its results were quantified using Micro Plate ELISA Reader Model ELx800. Serial dilutions of the calibrator were used to generate standard curve by plotting OD_450_ of each dilution on Y-axis and corresponding concentration on X-axis. The concentrations of trans-proteins were determined by finding their OD_450_ values on the curve and relating them to the corresponding concentration on X-axis.

### Leaf biotoxicity assay

Leaf biotoxicity assay was used to demonstrate toxic effect of *Cry1Ac* and *Cry2A* encoded endotoxins on larvae of shoot borer *chilo infuscatellus*. Leaves from transgenic as well as control plants at the age of 20, 40, 60 and 80 days were used for the biotoxicity assays. Leaves from each plant were used to feed three 2nd instar larvae in a petri dish. Each experiment was performed in triplicates. After 3 days, rate of mortality was measured with reference to control.

$$ {\text{\% Mortality = }}\frac{{{\text{No}}{\text{. of dead larvae}}}}{{{\text{Total no}}{\text{. of larvae}}}} \times 100 $$

### Glyphosate spray assay

Glyphosate spray (Roundup Ready Dupont Staple LX Plus USA) with active ingredient [(Pyrithiobac sodium, Sodium-2-Chloro-6(4,6 dimethoxy pyrimidine-2-yl) thio]benzoate) assay was used for physiological evaluation of *CEMB-GTGene* (modified CP4 EPSPS) encoded herbicide resistant protein. Glyphosate was sprayed at the optimized rate of 1200 mL/80 L H_2_O/acre on the sugarcane transgenic plants of V_0_ as well as V_1_ and V2 generations during field study. Same concentration of spray was also used on non-transgenic plants along with weeds present in the sugarcane field.

### Statistical analysis

Analysis of variance (ANOVA), least significant difference test (LSD), and Dunnett’s test were performed for comparison of significantly different mean values of different lines between the control plants and transgenic lines.

## Results

### Restriction digestion for the confirmation of sugarcane codon optimized synthetic *BT.* genes (*p*UC-57 vector)

The synthetic genes received in *p*UC-57 vector and confirmed through the Restriction Digestion and protein expressions analyses. Before cloning in plant expression vector (*p*CAMBIA-1301), CEMB-*Cry*1Ac, CEMB-*Cry*2A genes were analyzed through the restriction digestion by using Sph1 sites that released the fragments of 1.8 Kb and 1.9 Kb respectively. Restriction digestion with Sph-I also confirmed a fragment of 2.1 Kb for the Ubiquitin promoter. The undigested transformed vector (*p*UC-57) also shown in the (Supplementary Figure 2; Supplementary Tables 2 and 3).

### Confirmation of His-tagged protein expressions (CEMB-*Cry*1Ac + CEMB-*Cry*2A, CEMB-GTGene) through Western Blot analysis

**Figure 1:**
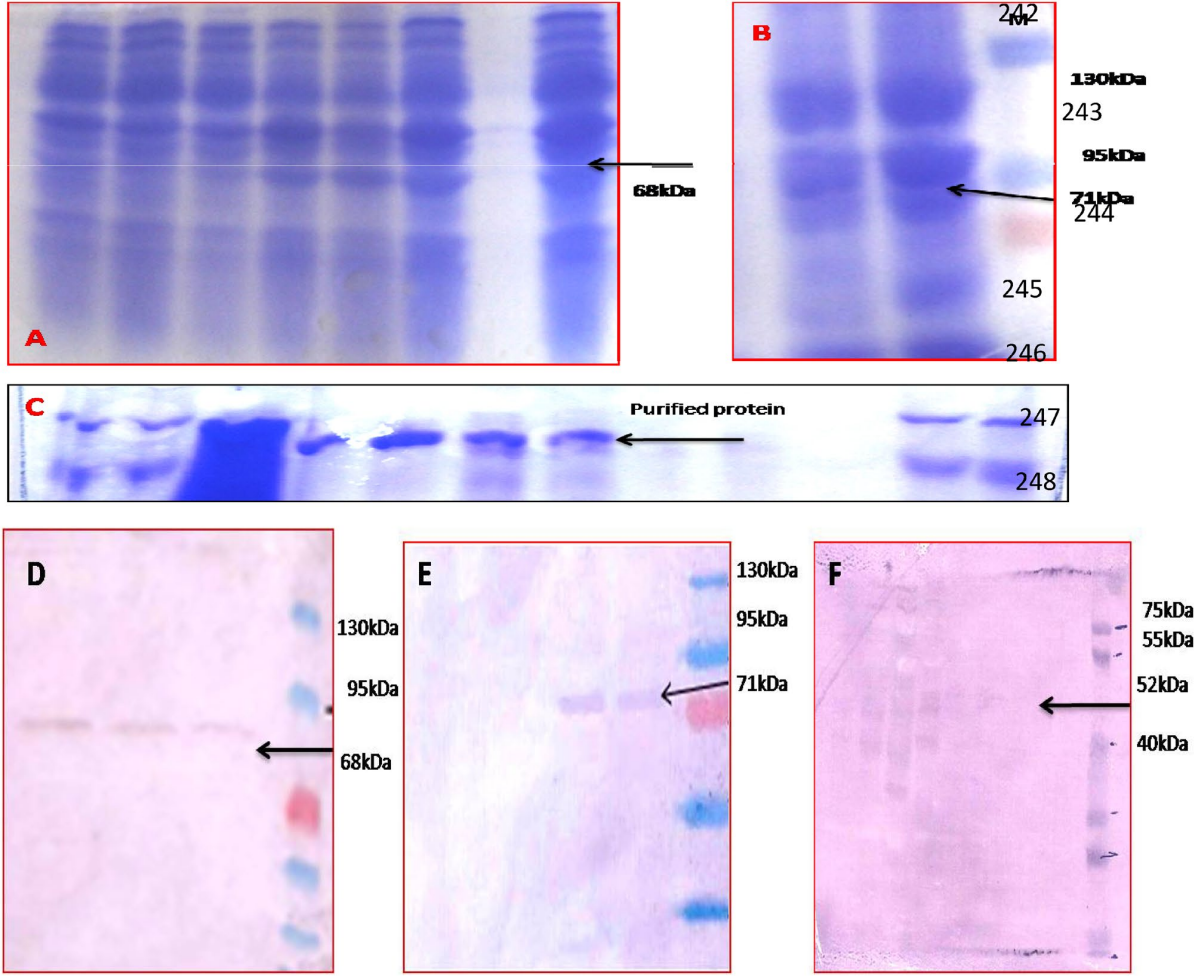
Protein expressions analyzed through SDS-PAGE and confirmed by Western Blot analysis. (**A**,**B**) SDS-PAGE of induced crude (1 mM-IPTG) proteins of CEMB-*Cry*1Ac, CEMB-*Cry*2A and CEMB-GTGene genes. (**C**) Purified proteins for Western Blot analysis. (**D**) Western Blot Analysis for CEMB-*Cry*1Ac protein, Lanes 1–3: CEMB-*Cry*1Ac protein (68 kDa), Lane M. Pre-strained protein ladder (Fermentas). (**E**) Western Blot Analysis for CEMB-*Cry*2Ac protein, Lanes 1–2: CEMB-*Cry*1Ac protein (71 kDa), Lane M: Pre-strained protein ladder (Fermentas). (**F**) Western Blot Analysis for CEMB-GTG protein, Lanes 1–4 CEMB-GTGene protein (54 kDa), Lane M. Pre-strained protein ladder (Fermentas).

For the confirmation of protein expressions, *p*ET-30 protein expression vector was constructed with CEMB-*Cry*1Ac, CEMB-*Cry*2A and CEMB-GTGene modified synthetic genes with KpnI, HindIII and SacI restriction sites. Physical mapping is drawn in the (Fig. 1A–C). The constructed plasmid vectors were transformed in Rosetta cells (*E. coli*) and analyzed on 10% SDS–Polyacrylamide gel (PAGE) stained with Coomassie blue. His-tagged proteins of CEMB-*Cry*1Ac (68 kDa), CEMB-*Cry*2A (71 kDa) and CEMB-GTG (52 kDa) were confirmed through Western Blot analysis (Fig. 1D–F) on nitrocellulose membranes (HyBond C, Amersham) through His-specific primary antibodies and AP-conjugated (Alkaline Phosphatase) secondary antibodies. For color development BCIP/NBT substrate tablets were used.

### Cloning of CEMB-*Cry*1Ac, CEMB-*Cry*2A, CEMB-GTGene in plant expression vector (*p*CAMBIA 1301)

After successful protein expressions of three genes through *p*ET-30 vector, for transformation, two final expression plasmids *p*CEMB-SC12 and *p*CEMB-SGTG were constructed to tailor the sugarcane with glyphosate tolerance, insect resistance and GUS as a reporter. The three synthetic genes were expressed from Maize Ubiquitin-1 for constitutive expression and NOS terminator for the stability of mRNA. CEMB-*Cry*1Ac + CEMB-*Cry*2A and CEMB-GTG expression cassettes were cloned in plant expression vector (*p*CAMBIA-1301) with the help of KpnI, SacI, SphI, and HindIII diagnostic sites. Restriction analysis of the final plant expression constructs confirmed the cloning of CEMB-*Cry*1Ac (4 kb), CEMB-*Cry*2A (4 kb) and CEMB-GTGene (1.4) (Supplementary Figure 3A,B). After confirmation, maxi-prep of the plasmids was done (Supplementary Figure 3C).

### Transient expression of transgene (GUS) to determine the efficacy of plasmid constructs

**Figure 2:**
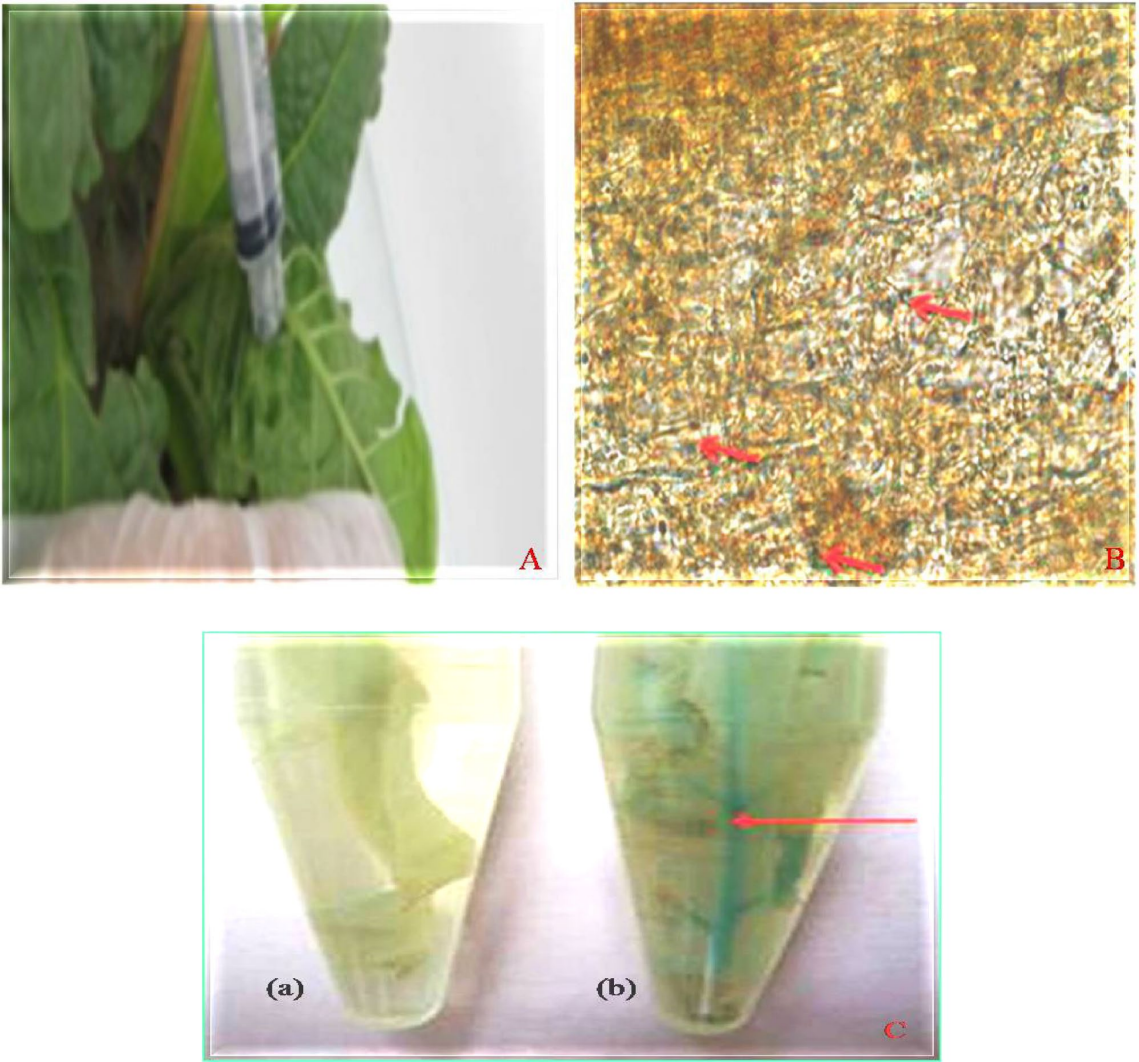
Through syringe method *Agro*-infiltrated Gus expression was efficiently observed in tobacco leaves. (**A**) *Agro*infiltrated tobacco leaves. (**B**) Bluish-green color under fluorescent microscope. (**C**) Visual GUS expression in Tobacco leaf (a): Non-*Agro*infiltrated tobacco leaves (negative control) (b): *Agro*infiltrated tobacco leaves predicting bluish-green color.

For the transient expression of GUS trasngnene in tobacco leaves, first of all these plant expression plasmids constructs *p*CEMB-SC12 and *p*CEMB-SGTG were introduced into *Agrobacterium tumefaciens* (LBA4404) by electroporation. The positive colonies of these constructs from test cultures were screened through colony PCR (Supplementary Figure 4A–C). The screened positive colonies were used for *Agro*-infiltration. The leaves were analyzed after 72 h. Under fluorescent micro scope (Olympus Szx7) bluish green color confirmed the GUS-transient expression and expression efficiency of the constructs (Fig. 2A–C).

### Selection of sugarcane variety for transformation and field study

In this study, SPF-213, SPF-234, HSF-240 and CPF-246 sugarcane varieties were selected on the basis of their cane yield (t/ha) and sugar recovery (%) for sugarcane tissue culturing and transformation (Supplementary Figure 5), however, after optimization of glyphosate spray assays the best responded variety was selected for further field studies.

### Tissue culture response of sugarcane cultivars

Diverse responses under tissue culture and regeneration of four varieties were noted. Four combinations for best callus induction media were used to obtain maximum embryogenic calli from these varieties. The MS Media 4.43 g/L, sucrose 30 g/L and phytagel 3 g/L were constant ingredients of every media combination. The maximum regeneration ability of SPF-234 (90%) was observed on media (RM-4) containing Kinetein (3 mg/L), BAP (3 mg/L)_,_ charcoal (1 g/L), Myo-inositol (200 mg/L)_._ The maximum shoot multiplication percentage of regenerated calli for CPF-246 (100%), SPF-234 (90%), SPF-213 (90%), and HSF-240 (81%), were recorded on media (SMM-2) consisting of Kinetin (2 mg/L), BAP (2 mg/L), GA3 (2 mg/L), IAA (1 mg/L) and charcoal (1 g/L). The sugarcane plants after multiplication were shifted on rooting media. The half strength media with NAA (1 mg/L) was used for rooting. These, one and a half month old rooted plants were acclimatized in the soil pots under green house conditions. From these selected sugarcane varieties CPF-246 revealed maximum regeneration and shoot multiplication (Supplementary Figure 6; Supplementary Tables 4 and 5).

### Biolistic transformation in sugarcane and selection of transgenic plants

**Figure 3:**
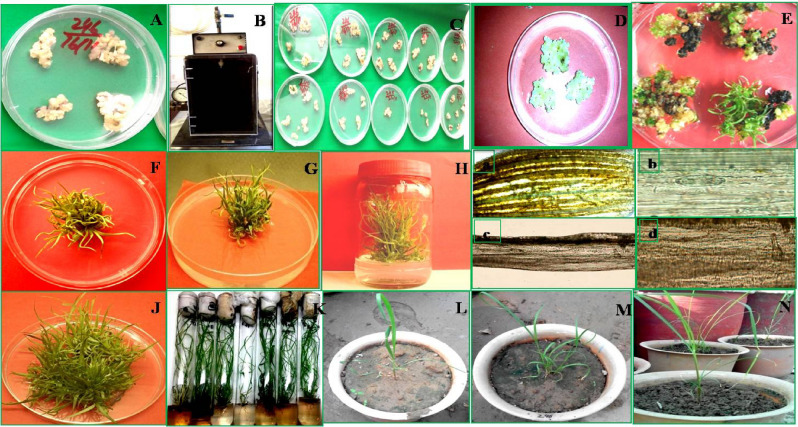
Schematic presentation of all the steps involved in genetic modification of sugarcane. (**A**) Callus for Bombardment. (**B**) Homemade Biolistic machine. (**C**) Bombarded Callus after bombardment with DNA-coated tungsten particles. (**D**) Bombarded callus shifted on selection media with Kanamycime (50 mg/L) after 2 days. (**E**,**F**) Transformed callus regenerated on double selection (Kanamycine 50 mg/L + Glyphosate 40 mM) media, (**G**,**H**) Regenerated sugarcane plantlets on glyphosate selection media (45 mM), shifting on shoot multiplication media with Kanamycime (50 mg/L) and glyphosate (50 mM) selections. (**I**) Gus Assay for transgenic plant screening (abcd). (**J**) Transgenic plants for rooting. (**K**) Shifting on rooting media without any selection drug. (**L**,**M**) Acclimatization: Transgenic sugarcane plantlets in soil pots under green house conditions.

Tungsten particles coated with *p*CEMB-SC12 and CEMB-GTGene plasmid constructs were used for Biolistic transformation. The CEMB-*Cry*lAc + CEMB-*Cry*2A and CEMB-GTG genes driven by Maize Ubiquitin-1 promoter with kanamycin as selection marker, were successfully transformed into four sugarcane varieties SPF-213, SPF-234, HSF-240, and CPF-246 by using CEMB homemade gene gun (Fig. 3). A total of 400 calli were used for the transformation process (Table 2). The transformed calli and regenerated transgenic sugarcane plants with these three genes were screened first on selection medium with only Kanamycine (50 mg/L), the response of resistant calli of four varieties SPF-213, SPF-234, HSF-240, and CPF-246 was as 70%, 74%, 45% and 91% respectively, while on double selection medium (kanamycine and glyphosate) 34% of SPF-213, 40% of SPF-234, 29% of HSF-240 and 81% of CPF-246 transformed calli survived (Supplementary Table 1). After shoot multiplication 1000 plantlets for each variety were obtained. From molecular analyses, PCR with glyphosate gene specific primers was carried out for this initial stage screening of these multiplied transgenic putative plants. From this screening survived plant shifted on rooting media were as 656, 680, 205, and 900 of CPF-213, CPF-234, HSF-240 and CPF-246 respectively. From PCR, Southern Blot, Dipstick and ELISA analyses 14 plants of CPF-213, 15 of CPF-234, 11 of HSF-240, and 30 of CPF-246 were positive for CEMB-*Cry*1Ac and 11 plants of CPF-213, 13 of CPF-234, 10 of HSF-240, and 15 of CPF-246 were positive for CEMB-*Cry*2A genes. For glyphosate tolerant gene, 27 plants of CPF-213, 39 of CPF-234, 15 of HSF-240, and 66 of CPF-246 were resulted positive through these analyses. Through these molecular analyses, 11 plants of CPF-213, 13 of CPF-234, 10 of HSF-240, and 15 of CPF-246 were considered three genes positive for protein expressions (Table 2). Stable putative transgenic sugarcane plants containing CEMB-*Cry*1Ac, CEMB-GTGene and CEMB-GTG genes were selected for further generation’s study. Finally, 15 lines of CPF-246 were selected for further generation’s studies under field conditions. These lines were further analyzed through molecular techniques to check the consistent trans-protein expression of the transformed genes in the next generations. For CPF-246, 66 transgenic plantlets were obtained after three screenings of glyphosate spray assays (2250 mL/80 L/hectare, 2750 mL/80 L/hectare, 3000 mL/80 L/hectare). Overall transformation efficiency of CPF-246 with respect to three genes protein expressions was 1.5%.

**Table 2:** Biolistic transformation in sugarcane and selection of transgenic plants.

Variety	Total no of calli bombarded	No of calli survived on kanamycine selection (%)	No of calli survived on kanamycine + glyphosate selection (%)	No of calli regenerated on selection medium (%)	Regenerated plantlets on selection media	Plantlets after GUS assay screening multiplied in the tubes	PCR (GTGene) screening and in the process of acclimatization	In tubes on rooting media	Shifted to green house in soil pots	Survived (one and half month old plants) after spray 900 mL/ 80 L/acre	In field	2-month old plants after glyphosate spray 1100 mL/80 L	Spray tolerant (1200 mL/80 L/acr) plants further analyzed for three genes through PCR, Southern Blot, DIPSTICK and ELISA	Positive for CEMB-*Cry*1Ac	Positive for CEMB-*Cry*2A	Positive for CEMB-GTGene	Three genes positive plants (transgenic plants survived in field after glyphosate spray (1200 mL/80 L/acre)	Transformation efficiency (%)
SPF-213	100	70	34	21	500	1000	800	752	656	200	122	54	27	14	11	27	11	1.1
SPF-234	100	74	40	32	600	1000	700	680	525	250	141	90	39	15	13	39	13	1.3
HSF-240	100	45	29	13	300	1000	320	205	125	75	60	40	15	11	10	15	10	1
CPF-246	100	91	81	48	950	1000	966	900	900	900	751	300	66	30	15	66	15	1.5
Total													147	71	58	147	49	

### Screening of sugarcane putative transgenic plants (CPF-246) with glyphosate resistance

**Figure 4:**
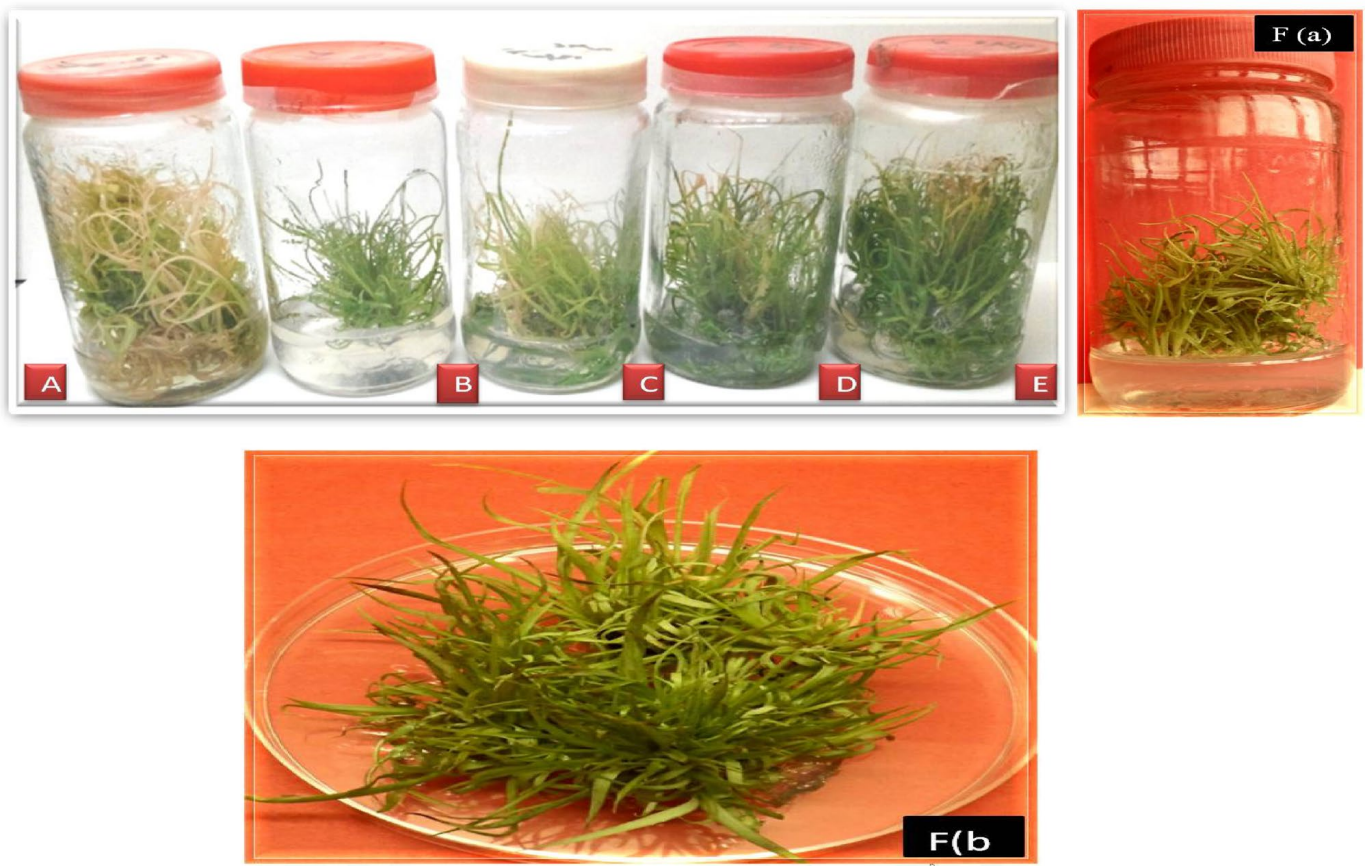
Sugarcane putative transgenic plantlets (CPF-246) on glyphosate selection medium. (**A**) Control (Non-Transgenic plants) Kanamycine (50 mg/L). (**B**) Kanamycine (50 mg/L). (**C**) Kanamycine (50 mg/L) + Glyphosate (40 mM). (**D**) Kanamycine (50 mg/L) + Glyphosate (45 mM). (**E**) Kanamycine (50 mg/L) + Glyphosate (50 mM). (**Fa**) Resistant Plant on Kanamycine (50 mg/L) + Glyphosate (50 mM). (**Fb**) Resistant transgenic sugarcane pants multiplied on Kanamycin (50 mg/L) and Glyphosate (50 mM) media.

For the optimization of glyphosate resistance four selection pressures were given to the transformed callus as well as regenerated transgenic and control plantlets (Fig. 4). After, 48 h of bombardment the transformed calli were transferred on kanamycine (50 mg/L) selection media. Following one week, survived calli were shifted to the regeneration media (RM-1 and RM-2) containing Kanamycine 50 mg/L and Glyphosate 40 mM. After 2-weeks resistant transgenic plants were further multiplied on shoot multiplication media (SMM-2) contained glyphosate concentration (50 mM).

### GUS transient expression for the screening of transgenic sugarcane plants

**Figure 5:**
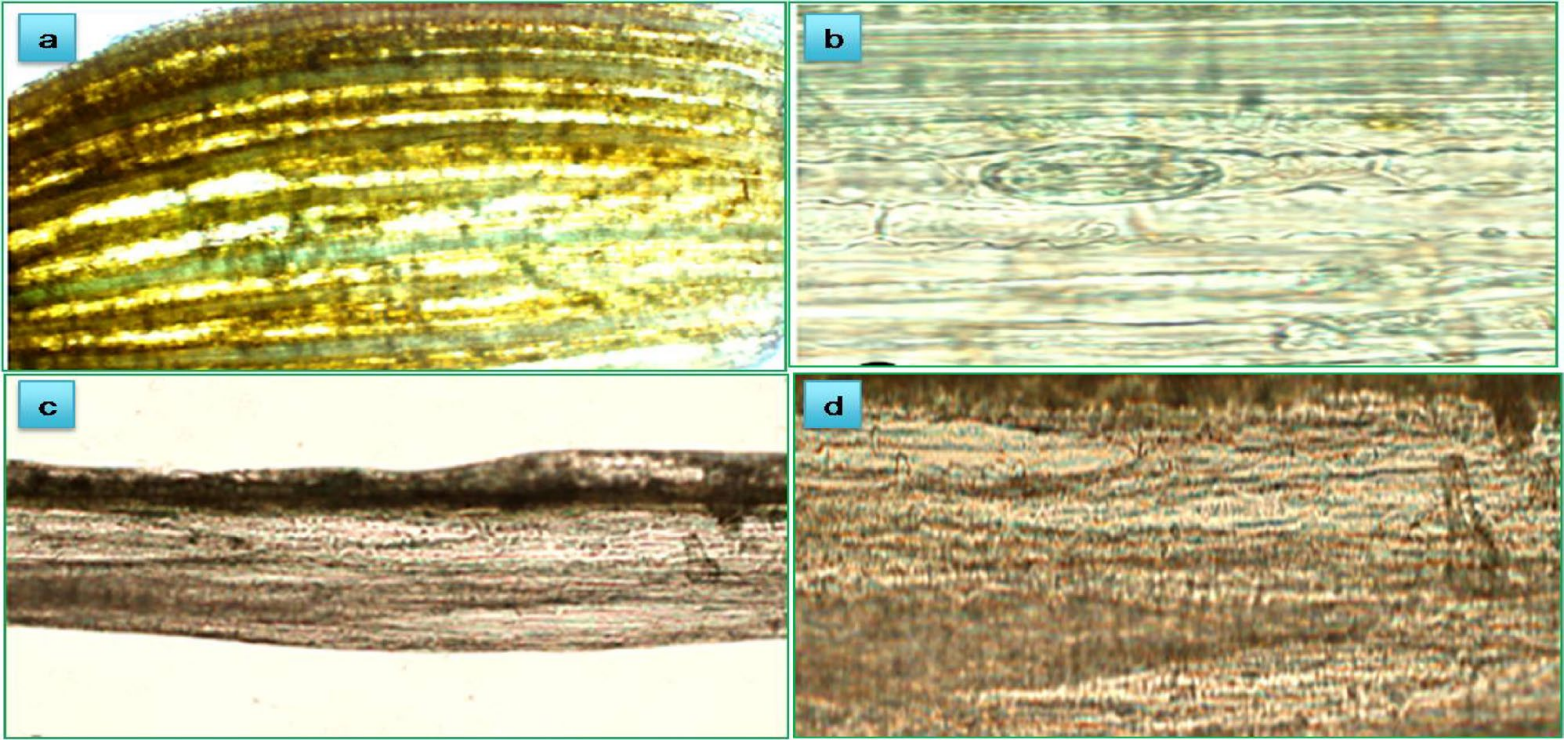
GUS Assay of transgenic sugarcane plants by using fluorescent microscope. (**a**,**b**) Young transgenic leaf. (**c**,**d**) Immature transgenic stem portion.

Plasmid constructs *p*CEMB-SC12 and *p*CEMB-SGTG was transformed in the sugarcane calli through Biolistic delivery system. These calli containing three transgenes and were regenerated on double selection pressure Kanamycine (50 mg/L) and Glyphosate (45 mM). Young regenerating plants from the screened resistant calli were further selected through the GUS assay. The transgenic expression of GUS assay of reporter gene was done for early detection of transgenic events. After visual observation, GUS expression in leaves and stems is further confirmed by a fluorescent microscope, cells were showing uniform expression through its evenly distributed bluish green color in cross sections, depicting the success of whole transformation process (Fig. 5).

### Molecular analyses of putative transgenic sugarcane plants at v_0_ generation

The putative transgenic sugarcane plants containing CEMB-*Cry*1Ac + CEMB-*Cry*2A and CEMB-GTGene were subjected to molecular analyses i.e. PCR, Southern DNA Hybridization Dipstick assay and ELISA.

### PCR screening of three genes CEMB-*Cry*1AC, CEMB-*Cry*2A and CEMB-GTG positive putative transgenic plants (V_0_)

**Figure 6:**
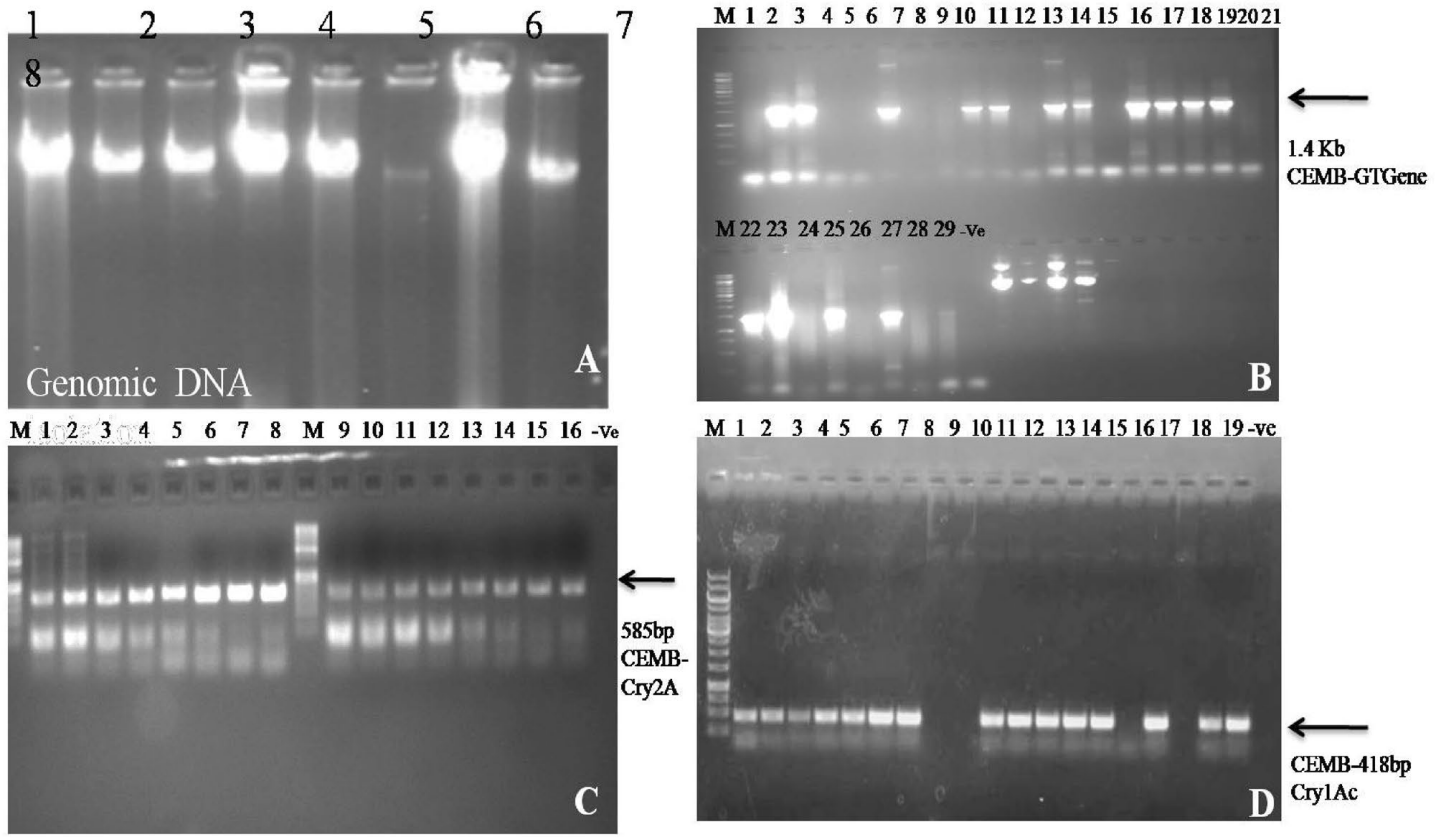
PCR screened positive plants of CPF-246 plant containing CEMB-GTGene, CEMB-*Cry*2A and CEMB-*Cry*1Ac. (**A**) Genomic DNA isolated from putative sugarcane plants. (**B**) Lane M: 1 kb DNA Ladder, Lane 1–28: Putative transgenic sugarcane plants with CEMB-GTGene Lane 29: Positive control, Lane −ve: Non-transgenic plant (control plant). (**C**) Lane M: 1 kb DNA Ladder, Lane 1: Positive control, Lane 2–16: Putative transgenic sugarcane plants with CEMB-*Cry*1Ac Lane −ve: Non-transgenic plant (control plant). (**D**) Lane M: 1 kb DNA Ladder, Lane 1: Positive control Ladder, Lane 2–19: Screening of Putative transgenic sugarcane plants with CEMB-*Cry*2Ac, Lane −ve: Non-transgenic plant (control plant).

The glyphosate tolerant 15 putative transgenic plants of CPF-246 were designated as CPF-246 (2L/6), CPF-246 (2L/8),V- (3L/3), CPF-246 (3L/4), CPF-246 (4L/7), CPF-246 (4L/8), CPF-246 (5L/1), CPF-246 (5L/5), CPF-246 (6L/2), CPF-246 (6L/5), CPF-246 (7L/2), CPF-246 (7L/7), CPF-246 (L8/4),and CPF-246(L9L/6).These putative transgenic plants were positive for CEMB-*Cry*1Ac, CEMB-*Cry*2A, and GTGene genes as confirmed through PCR by using gene specific primers during screening process. The amplified products of 1.4 kb for CEMB-GTGene, 418 bp for CEMB-*Cry*1Ac and 580 bp for CEMB-*Cry*2A confirmed the integration of three genes in the genome so referred these plants for further molecular analyses. No amplification was found in non transgenic plants (Fig. 6A–D).

### Southern Blot analysis

**Figure 7:**
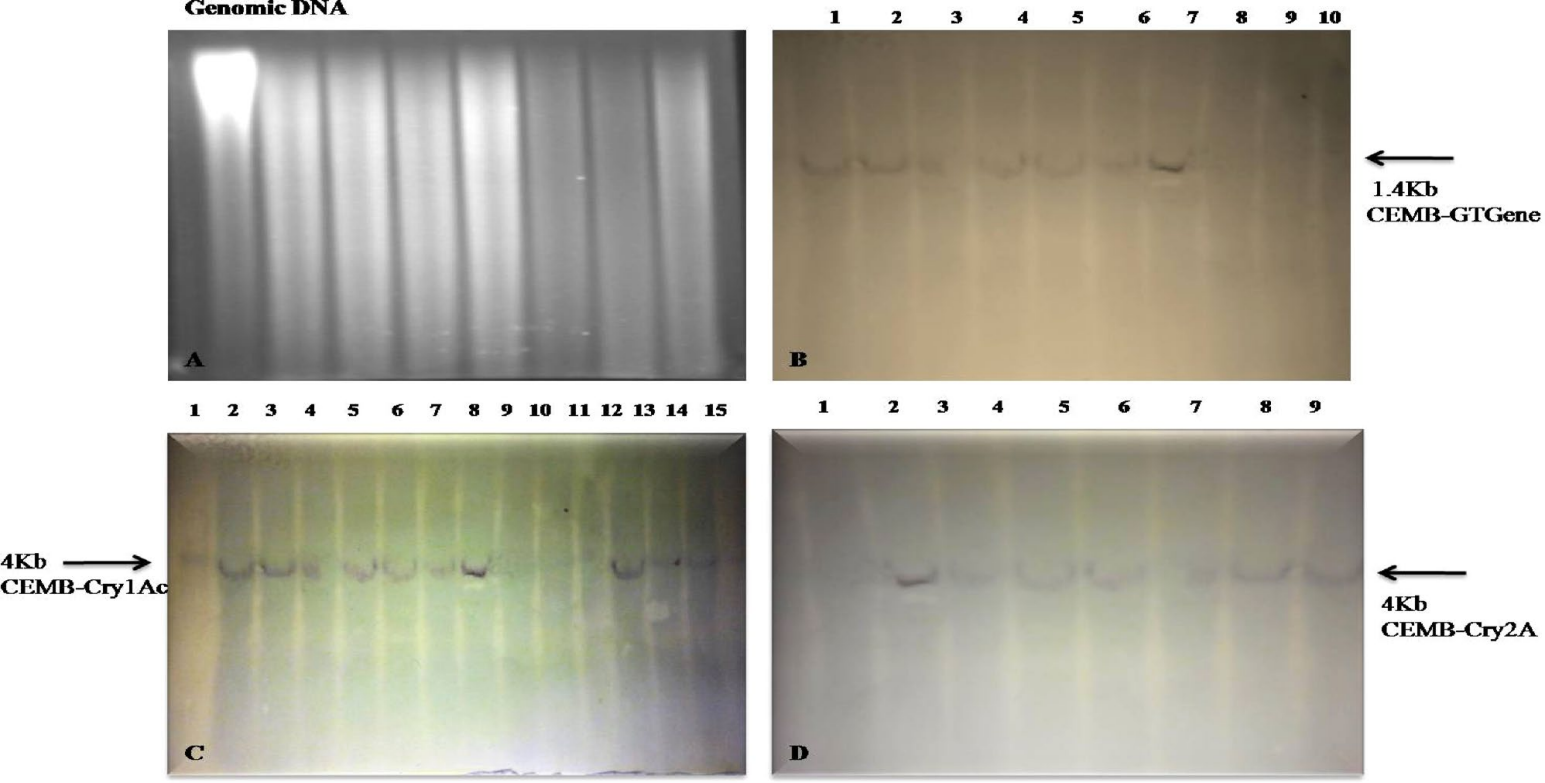
Southern Blot analysis of the transgenic sugarcane plants (CEMB-*Cry*1Ac, CEMB-*Cry*2A and CEMB-GTGene) in V_0_-generation. (**A**) Denaturing of genomic DNA. (**B**) Lane 1: Positive control, Lane 2–14: Southern DNA hybridization of transgenic sugarcane plants with CEMB-GTGene probe. (**C**) Lane 1: Positive control, Lane 2–15: Southern DNA hybridization of transgenic sugarcane plants with CEMB-*Cry*1Ac probe. (**D**) Lane 1: Positive control, Lane 2–9: Southern DNA hybridization of transgenic sugarcane plants with CEMB-*Cry*2A probe.

The stable integration of three genes CEMB-*Cry*1Ac, CEMB-*Cry*2A and CEMB-GTGene in the putative transgenic sugarcane plants was confirmed by the Southern Blot analysis through probe binding and its succeeding detection with the substrate. The PCR positive putative transgenic plants CPF-246 (2L/6), CPF-246 (2L/8), V- (3L/3), CPF-246 (3L/4), CPF-246 (4L/7), CPF-246 (4L/8), CPF-246 (5L/1), CPF-246 (5L/5), CPF-246 (6L/2), CPF-246 (6L/5), CPF-246 (7L/2), CPF-246 (7L/7), CPF-246 (L8/4), and CPF-246 (L9L/6) were analyzed for gene integration. Their respective probes highlighted the integration of the transgenes when the plant genomic DNA was digested with enzymes, KpnI-SacI for CEMB-GTGene, KpnI and HindIII for CEMB-*Cry*1Ac and CEMB-*Cry*2A and hybridized with probes. This hybridization with the specific probes confirmed the stable integrity of all three transgenes in the genome of these 15 sugarcane plants. The CEMB-GTGene with 1.4 kb, CEMB-*Cry*1Ac with 4 kb and CEMB-*Cry*2A with 4 kb fragments (full cassettes) were revealed by Southern Blot analysis (Fig. 7A–D).

### Dipstick assay

**Figure 8:**
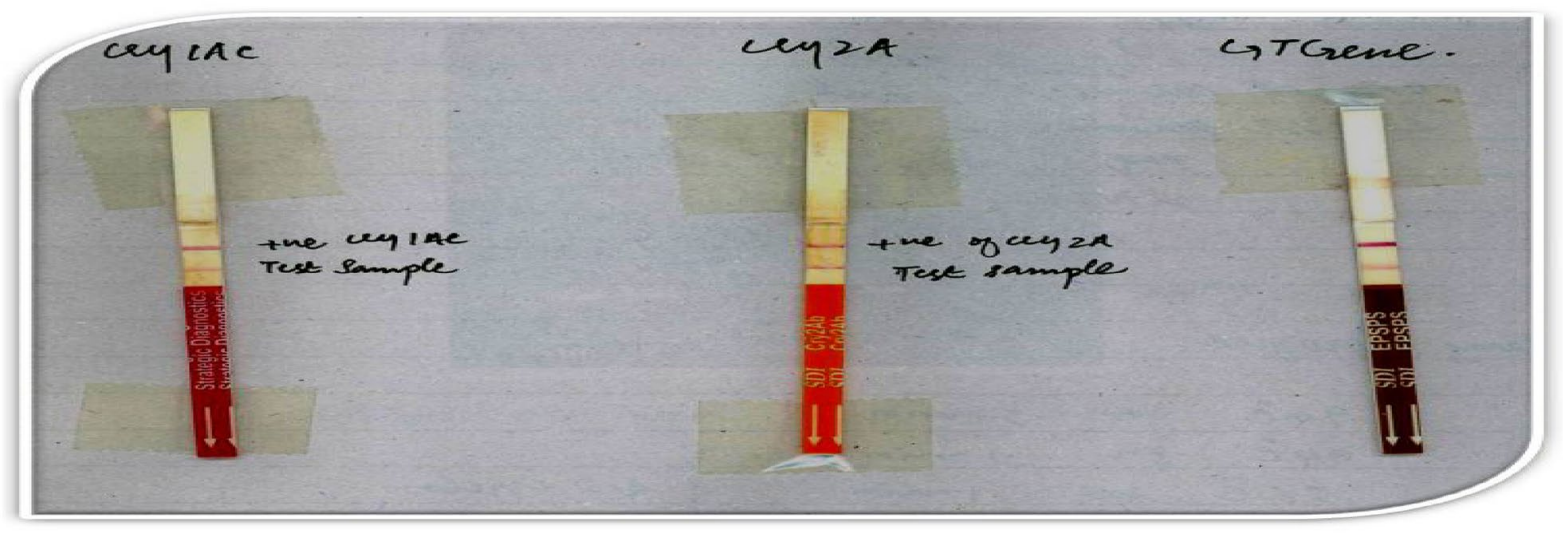
ELISA-Dipstick assay for the expression confirmation of three trans-proteins CEMB-*Cry*1Ac, CEMB-*Cry*2A and CEMB-GTGene in V_0_-generation. Lane 1: ELISA-Dipstick Positive for CEMB-*Cry*1Ac transgenic plants. Lane 2: ELISA-Dipstick Positive for CEMB-*Cry*2A transgenic plants. Lane 3: ELISA-Dipstick Positive for CEMB-GTGene transgenic plants.

All the 15 putative transgenic plants CPF-246 (2L/6), CPF-246 (2L/8),V- (3L/3), CPF-246 (3L/4), CPF-246 (4L/7), CPF-246 (4L/8), CPF-246 (5L/1), CPF-246 (5L/5), CPF-246 (6L/2), CPF-246 (6L/5), CPF-246 (7L/2), CPF-246 (7L/7), CPF-246 (L8/4), and CPF-246 (L9L/6) were positive for Dipstick assay that confirmed the protein expressions of the CEMB-*Cry*1Ac,CEMB-*Cry*2A and CEMB-GTGene transgenes into the genome of putative transgenic sugarcane plants (Fig. 8).

### Enzyme linked immuno-sorbent assay (ELISA) in V_0_-generation

The ultimate objective of the sugarcane transformation experiments was to get maximum expression of these transgenes (CEMB-*Cry*1Ac, CEMB-*Cry*2A and CEMB-GTGene) in sugarcane. Enzyme linked Immuno-sorbant Assay was done to quantify the proteins for CEMB-*Cry*1Ac, CEMB-*Cry*2A and CEMB-GTGene. The total crude protein was extracted from under study plant leaves (Supplementary Figure 7; Supplementary Table 6). The proteins from putative transgenic sugarcane plants were coated in the ELISA plates. The detections of the respective transprotein from transformed plants were confirmed after specific antibodies with the appearance of yellow color. Quantification of CEMB-*Bt*. and CEMB-GTGene proteins of 15 clones was done through ELISA, maximum 0.475 µg/g protein of CEMB-*Cry*1Ac from fresh leaves protein was acquired, 0.567 µg/g CEMB-*Cry*2A of fresh leaves protein was obtained, while for CEMB-GTGene maximum 0.486 µg/g of total leaf protein was acquired as represented in (Supplementary Table 7; Supplementary Figures 8–10). Based on ELISA quantification, comparison between proteins of CEMB-*Cry*1Ac CEMB-*Cry*2A and CEMB-GTGene was also made between transgenic sugarcane plants of V_0_-generation (Supplementary Figures 10, 11).

### Leaf bio-assay assay of the transgenic sugarcane

**Figure 9:**
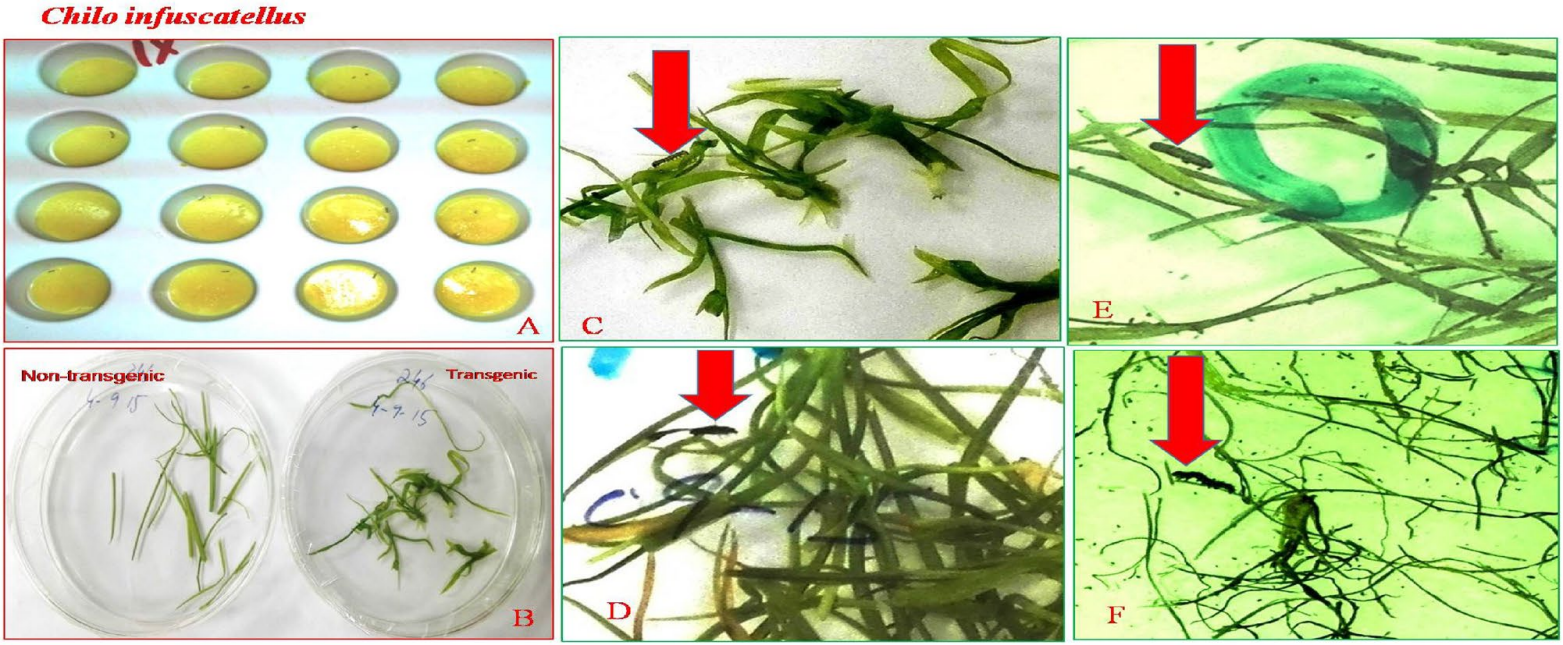
Leaf bioassay of transgenic plant leaves and control sugarcane plants. Plate (**A**) *Chilo infuscatellus* from CEMB-insectray Lab. Plate (**B**) Non-transgenic sugarcane and transgenic plant leaves after 20 days. Plate (**C**) *Chilo infuscatellus* with transgenic sugarcane leaves. Plate (**D**,**E**) Dead cane borer, 3rd day of infestation. Plate (**F**) *Chilo infuscatellus* is alive and healthy with control; non transgenic sugarcane leaves.

**Figure 10:**
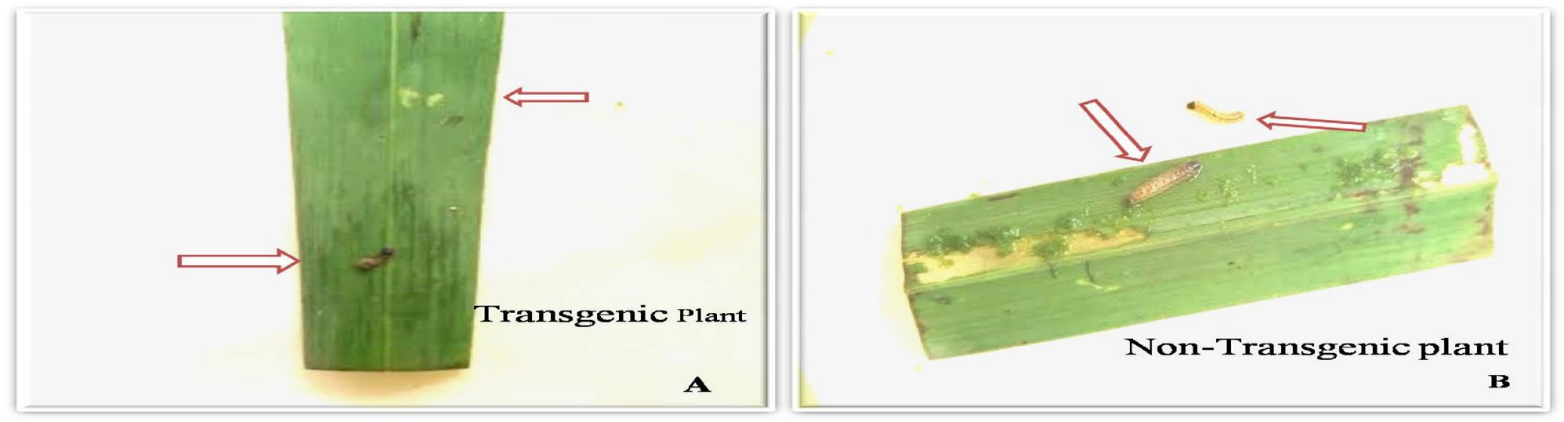
Leaf bioassay of 80 days old transgenic and non transgenic sugarcane. Plate (**A**) Transgenic sugarcane leaves killed *Chilo infuscatellus* larvae. Plate (**B**) Non-transgenic sugarcane plant. *Chilo infustcatellus*, alive and actively growing.

**Figure 11:**
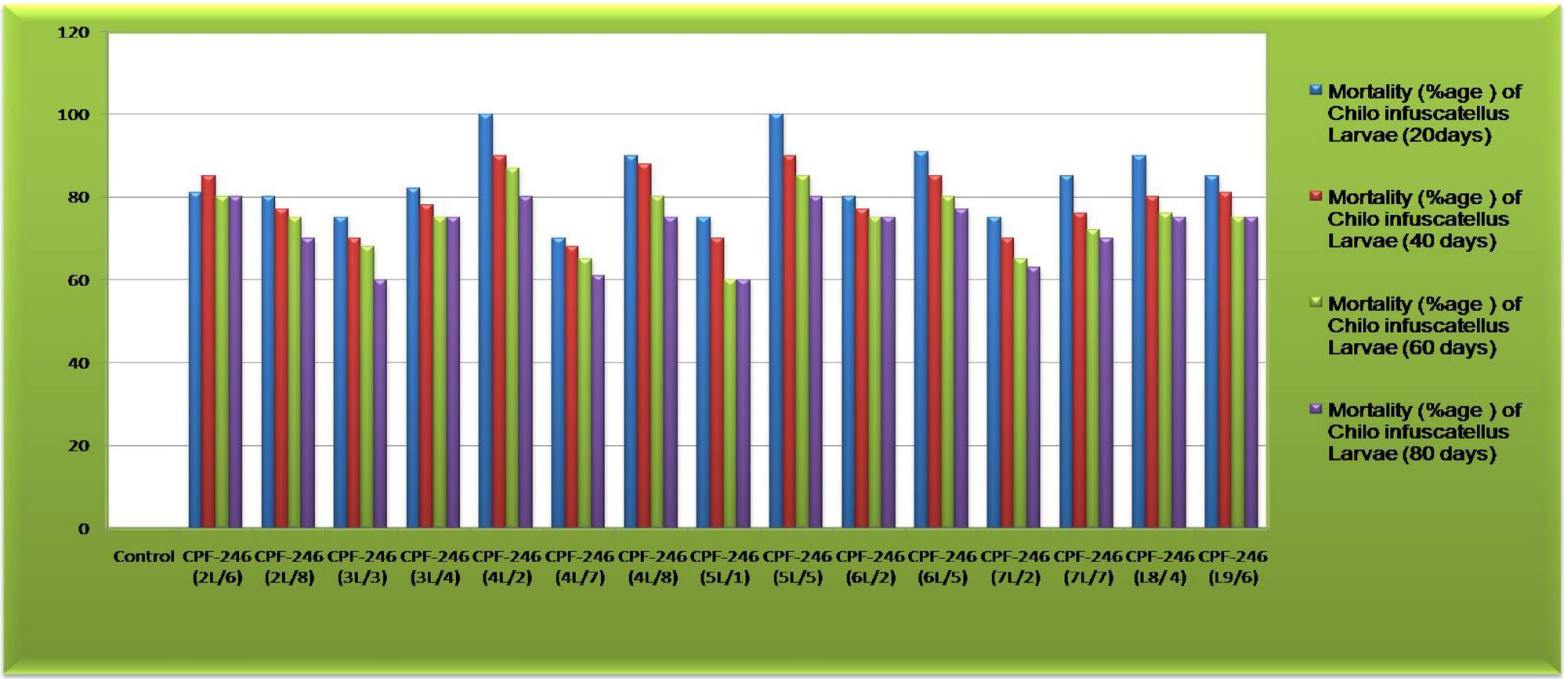
Graphical presentation of morality percentages of *Chilo infuscatellus* against putative transgenic sugarcane plants at the plant age of 20, 40, 60 and 80 days in V_0_-generation.

To interpret the efficacy of the CEMB-*Cry*lAc, CEMB-*Cry*2A and CEMB-GTGene transproteins in transgenic sugarcane plant, immature leaf portions were fed to the larvae of *Chilo infuscattellus* at the plant age of 20, 40, 60 and 80 days in the Petri plates. The calculated mortality of the *Chilo infuscattellus* larvae ranged from 60 to 100% against transgenic sugarcane leaves, whereas in non-transgenic plants, all insects were active, alived and showed increased weight. The results showed that the transgenic sugarcane plants have such enough quantities of CEMB-*Bt*.toxins that can kill the cane borers even at initial stages of the plant growth (Figs. 9, 10, 11; Table 3).

**Table 3:** Leaf bio-toxicity assay of transgenic sugarcane plants from V_0_-generation.

Sugarcane plants	Leaf bio-assay, mortality percentage of *Chilo infuscatellus* larvae at different ages of transgenic plants (days)			
	20 days	40 days	60 days	80 days
Control	0	0	0	0
CPF-246 (2L/6)	81	85	80	80
CPF-246 (2L/8)	80	77	75	70
CPF-246 (3L/3)	75	70	68	60
CPF-246 (3L/4)	82	78	75	75
CPF-246 (4L/2)	100	90	87	80
CPF-246 (4L/7)	70	68	65	60
CPF-246 (4L/8)	90	88	80	75
CPF-246 (5L/1)	75	70	60	60
CPF-246 (5L/5)	100	90	85	80
CPF-246 (6L/2)	80	77	75	75
CPF-246 (6L/5)	91	85	80	77
CPF-246 (7L/2)	75	70	65	63
CPF-246 (7L/7)	85	76	72	70
CPF-246 (L8/4)	90	80	76	75
CPF-246 (L9/6)	85	81	75	75

Numbers in brackets are representing the event nos.

### Glyphosate spray assay in V_0_-generation

**Figure 12:**
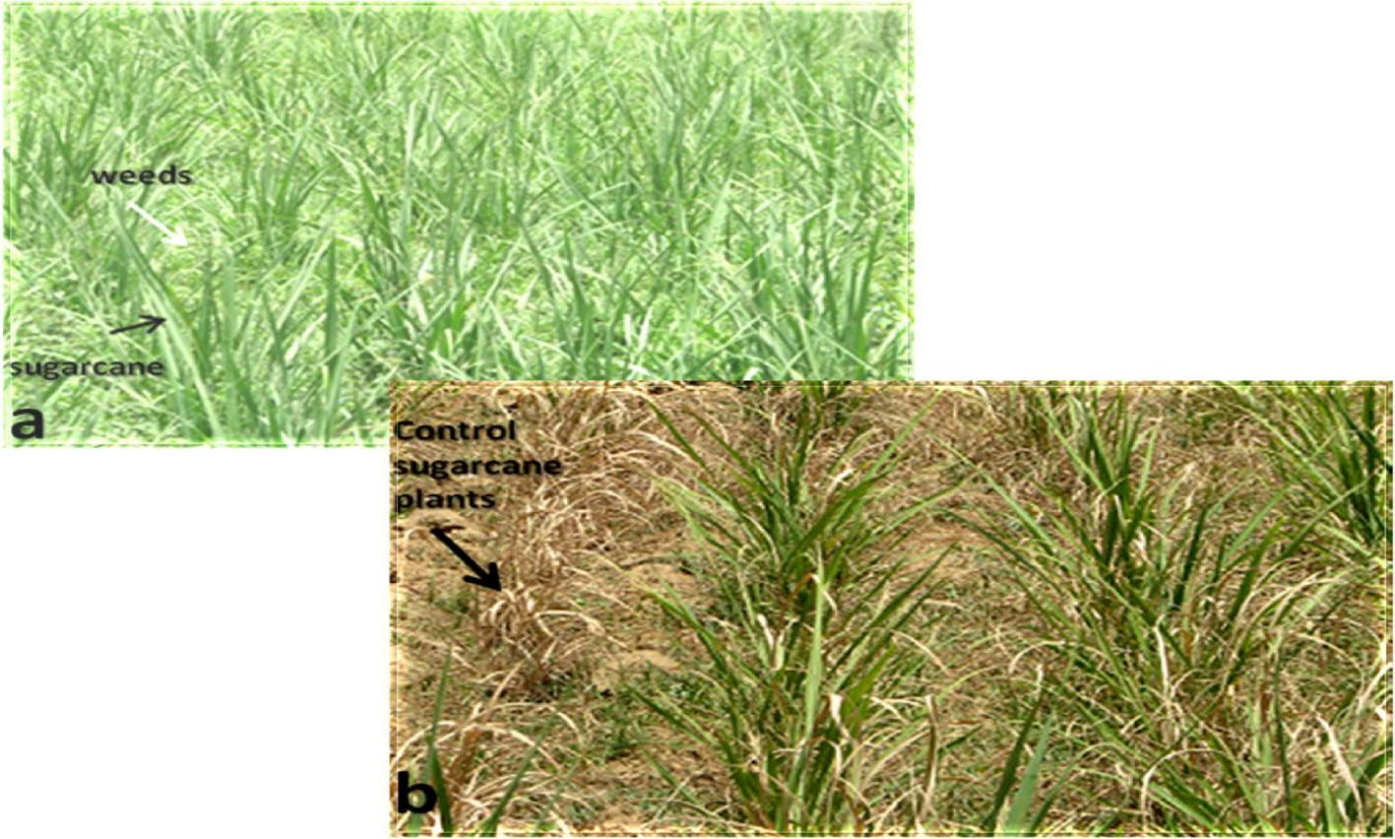
Glyphosate herbicide spray assay (1200 ml/acre) of sugarcane transgenic plants and Weed/non-transgenic (V_0_-genearation). (**a**) Transgenic sugarcane field plants before spray. (**b**) Non-transgenic plant and weeds died after glyphosate spray (1200 ml/80 L/acre), while survived transgenic sugarcane plants in the field after 5–7 days of spray.

Spray assays were carried out to optimize the tolerance level of transgenic sugarcane plant against glyphosate under open environmental conditions in V_0_-generation (1200 mL/80 L/acre). Finally, Roundup, sprayed at the proportion of 1200 ml/acre on the field plants. After 5–7 days, the weed/herbs and control plants that first shown necrotic patches, dead tissues ultimately resulted into complete withering of plant (100% mortality) (Fig. 12). The sugarcane transgenic plants CPF-246 (2L/6), CPF-246 (2L/8), V- (3L/3), CPF-246 (3L/4), CPF-246 (4L/7), CPF-246 (4L/8), CPF-246 (5L/1), CPF-246 (5L/5), CPF-246 (6L/2), CPF-246 (6L/5), CPF-246 (7L/2), CPF-246 (7L/7), CPF-246 (L8/4), and CPF-246 (L9L/6) were tolerant for glyphosate spray at the rate of 1200 mL/80 L/acre (Supplementary material Table 8).

### Inheritance of CEMB-*Cry*1Ac, CEMB-*Cry*2A and CEMB-GTGene genes in sugarcane V_1_-generaton

These 15 putative transgenic sugarcane plants of CPPF-246, resistant against cane borers and tolerant for glyphosate herbicide spray assay (1200 ml/acre) were selected further for generation’s advancement (Table 4) and evaluation by molecular analyses like PCR with amplified products 418 for CEMB-*Cry*1Ac, 585 bp for CEMB-*Cry*2A and 1.4 kb for CEMB-GTGene (Fig. 15a–c), Southern Blot analysis (Fig. 16a–d), Dipstick assay and ELISA (Supplementary Figures 12–16) in V_1_-generation. Insect Bioassay and Glyphosate Herbicide Spray assay were also carried out (Supplementary material Table 9). Like Dunnett and Tamhane^41^, statistical analyses such as Analysis of Variance, least significant difference test (LSD) and Dunnett’s test were also applied for the evaluation of data from transgenic sugarcane plants in V_1_-generation. Fifteen lines each with 10 replicates (nodes) (15 × 10), total 150 transgenic plants in V_1_-generation were field studied. Initially randomly selected plants during field study were analyzed from V_1_-generation. All lines were highly cane borer (*Chilo infuscatellus*) resistant and efficiently glyphosate tolerant.

**Table 4:** Summary of molecular analysis of randomly selected transgenic sugarcane plants (V_1_-generation).

Transgenic lines of sugarcane (CPF-246)	PCR			Southern Blot			Dipstick assay			ELISA (µg/g)			Glyphosate spray assay (1200 mL/acre)
	CEMB-*Cry*1Ac	CEMB-*Cry*2A	CEMB-GTG	CEMB-*Cry*1Ac	CEMB-*Cry*2A	CEMB-GTG	CEMB-*Cry*1Ac	CEMB-*Cry*2A	CEMB-GTG	CEMB-*Cry*1Ac	CEMB-*Cry*2A	CEMB-GTG	
CPF-246 (2L/6-3) 1	+VE	+VE	+VE	+VE	+VE	+VE	+VE	+VE	+VE	0.490	0.411	0.451	Tolerant
CPF-246 (2L/8-5) 2	+VE	+VE	+VE	+VE	+VE	+VE	+VE	+VE	+VE	0.410	0.480	0.432	Tolerant
CPF-246 (3L/3-8) 3	+VE	+VE	+VE	+VE	+VE	+VE	+VE	+VE	+VE	0.510	0.441	0.421	Tolerant
CPF-246 (3L/4-4) 4	+VE	+VE	+VE	+VE	+VE	+VE	+VE	+VE	+VE	0.464	0.542	0.434	Tolerant
CPF-246 (4L/2-7) 5	+VE	+VE	+VE	+VE	+VE	+VE	+VE	+VE	+VE	**0.672**	**0.642**	**0.569**	Tolerant
CPF-246 (4L/7-1) 6	+VE	+VE	+VE	+VE	+VE	+VE	+VE	+VE	+VE	0.456	0.467	0.459	Tolerant
CPF-246 (4L/8-4) 7	+VE	+VE	+VE	+VE	+VE	+VE	+VE	+VE	+VE	0.552	0.560	0.454	Tolerant
CPF-246 (5L/1-7) 8	+VE	+VE	+VE	+VE	+VE	+VE	+VE	+VE	+VE	0.345	0.423	0.458	Tolerant
CPF-246 (5L/5-2) 9	+VE	+VE	+VE	+VE	+VE	+VE	+VE	+VE	+VE	**0.678**	**0.599**	**0.578**	Tolerant
CPF-246 (6L/2-10) 10	+VE	+VE	+VE	+VE	+VE	+VE	+VE	+VE	+VE	0.458	0.523	0.455	Tolerant
CPF-246 (6L/5-3) 11	+VE	+VE	+VE	+VE	+VE	+VE	+VE	+VE	+VE	**0.687**	**0.611**	**0.589**	Tolerant
CPF-246 (7L/2-1) 12	+VE	+VE	+VE	+VE	+VE	+VE	+VE	+VE	+VE	0.489	0.478	0.458	Tolerant
CPF-246 (7L/7-7) 13	+VE	+VE	+VE	+VE	+VE	+VE	+VE	+VE	+VE	0.521	0.455	0.404	Tolerant
CPF-246 (L8/4-10) 14	+VE	+VE	+VE	+VE	+VE	+VE	+VE	+VE	+VE	**0.642**	**0.648**	**0.551**	Tolerant
CPF-246 (L9/6-9) 15	+VE	+VE	+VE	+VE	+VE	+VE	+VE	+VE	+VE	**0.652**	**0.622**	**0.564**	Tolerant

Bold values indicate higher protein concentration

### Inheritance of CEMB-***Cry***1Ac, CEMB-***Cry***2Ac and CEMB-GTGene genes in sugarcane lines of V_2_-generation

**Figure 13:**
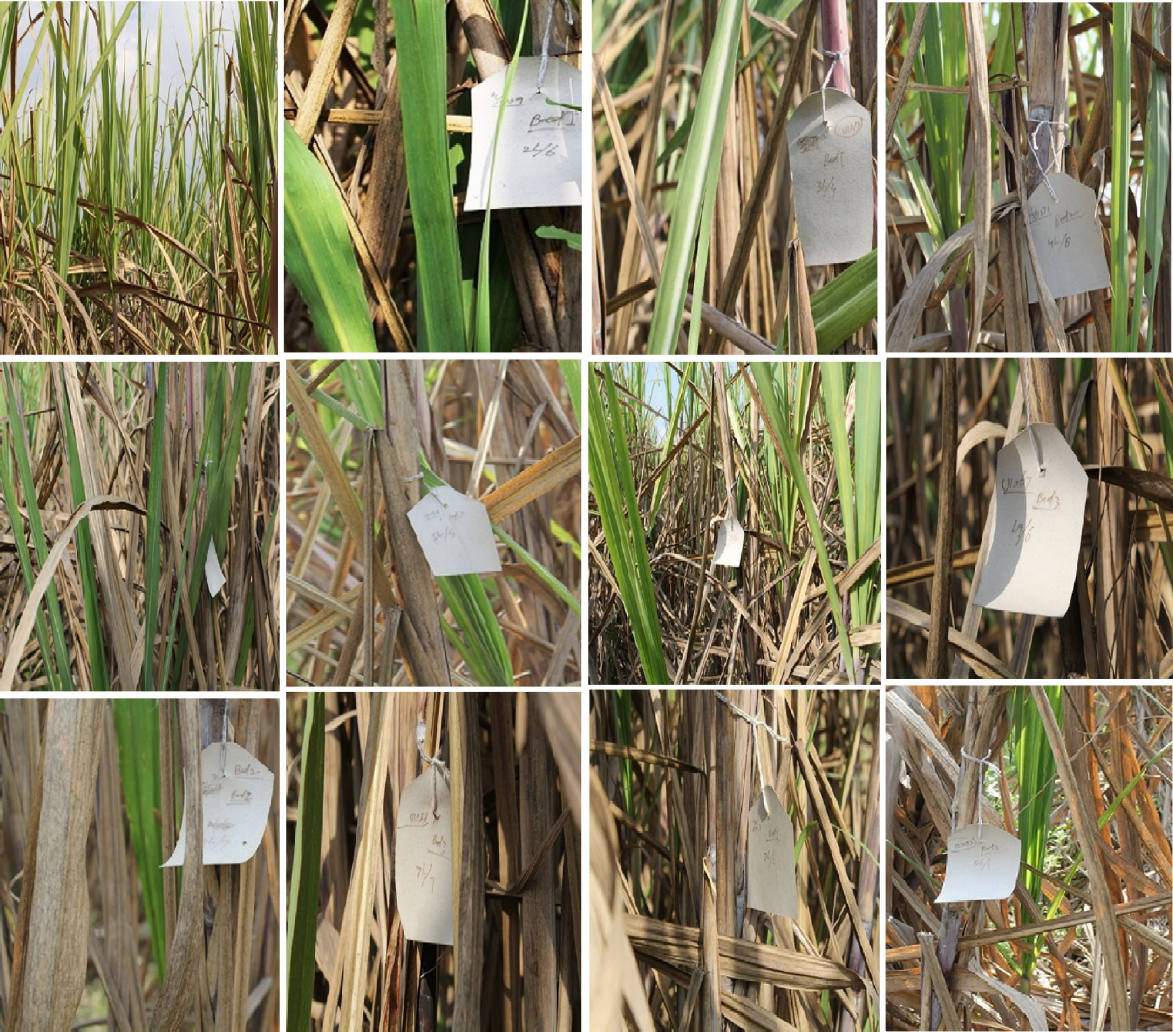
Glyphosate tolerant and cane borer resistant lines of V_1_-generation during the field studies.

**Figure 14:**
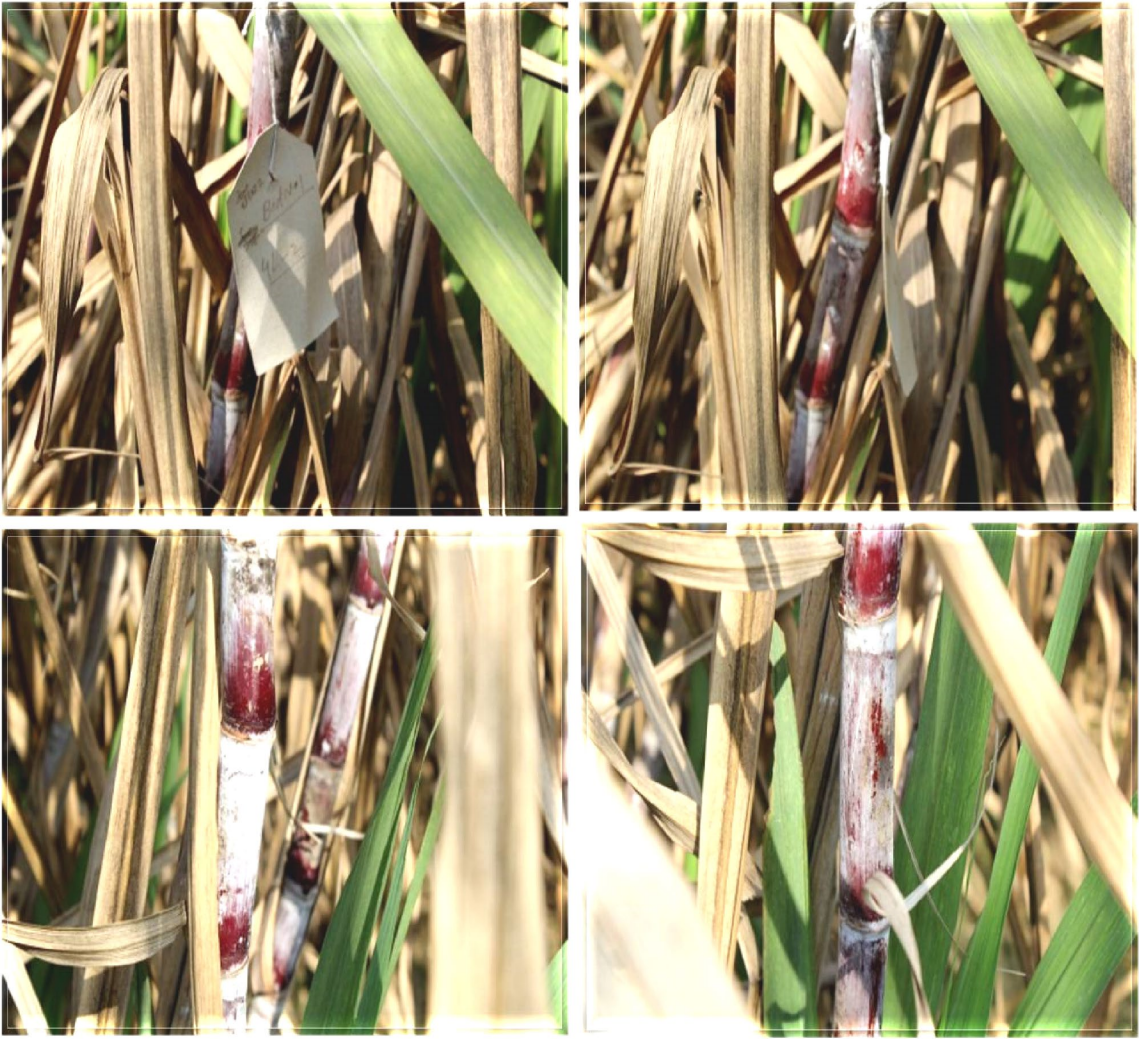
Five sugarcane transgenic lines from 15 V_1_-generation free of any kind of insect attack and tolerant to glyphosate spray 3000 mL/ha) were screened as V_2_-generation.

**Figure 15:**
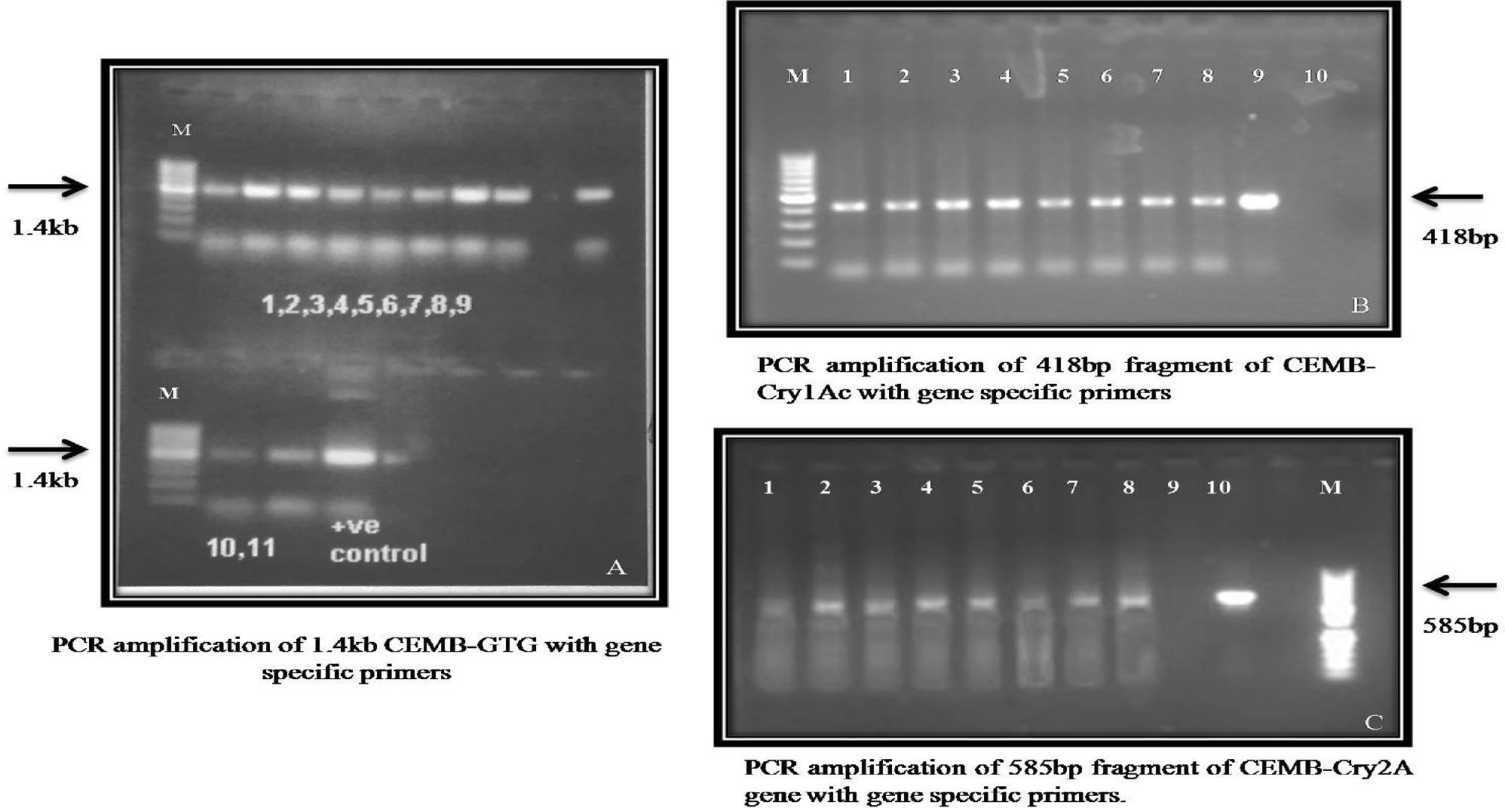
PCR amplified transgenic plants of CPF-246 for CEMB-*Cry*1Ac, CEMB-*Cry*2A and CEMB-GTGene. (**A**) Lane M: 1 kb DNA Ladder, Lane 1–11: Putative transgenic sugarcane plants with CEMB-GTGene (1.4 kb) Lane + ve: Positive control Lane 13: Non-transgenic plant (control plant). (**B**) Lane M: 1 kb DNA Ladder Lane 1–8: Putative transgenic sugarcane plants with CEMB-*Cry*1Ac (418 bp) Lane 9: Positive control Lane 10: Non-transgenic plant (control plant). (**C**) Lane M: 1 kb DNA Ladder Lane 1–11: 1–8 Putative transgenic sugarcane plants with CEMB-*Cry*2A (585 bp) Lane 9: Non-transgenic plant (control plant), Lane 10: Positive control.

**Figure 16:**
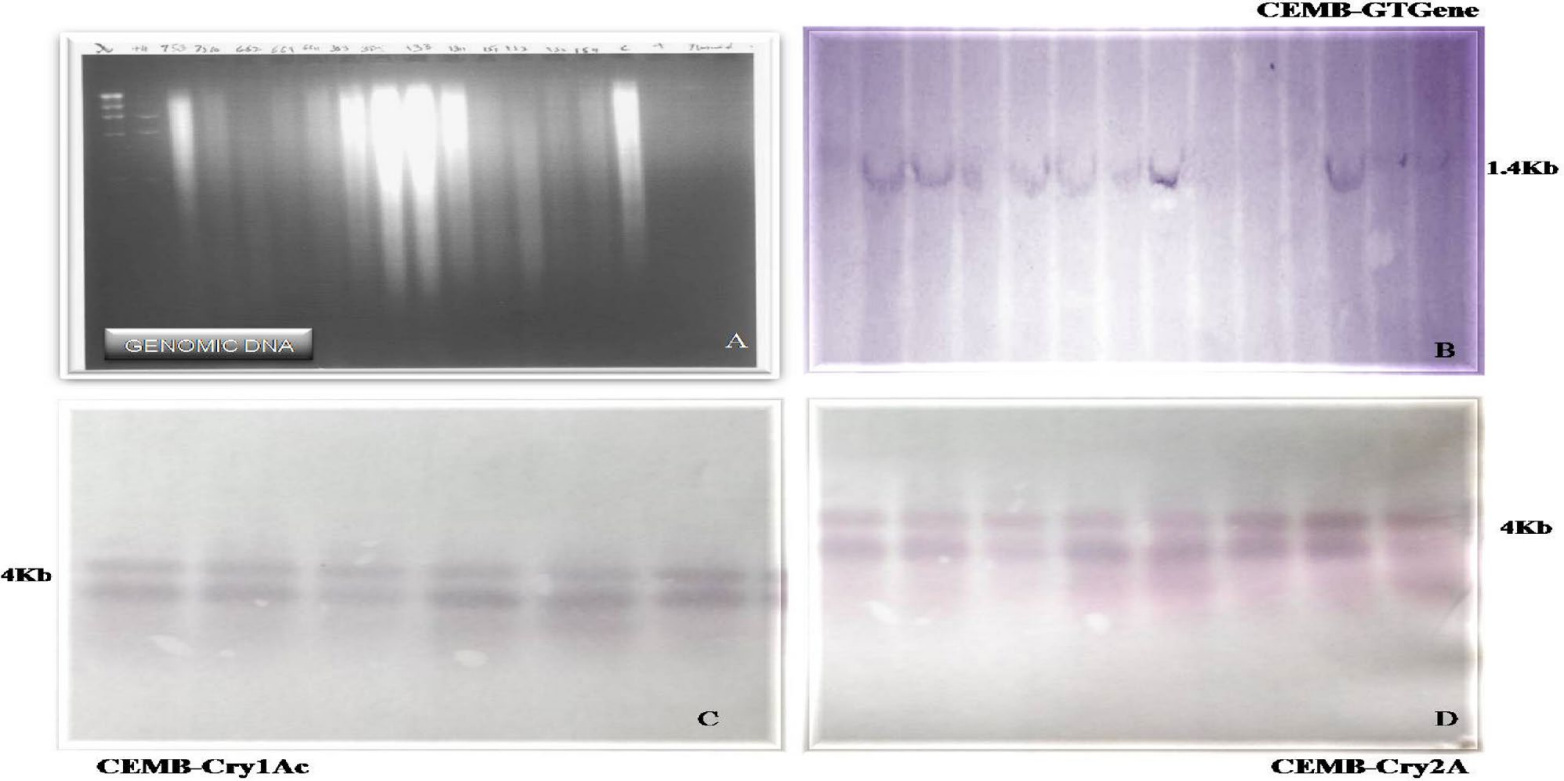
Southern Blot analysis of the transgenic sugarcane plants (CEMB-*Cry*1Ac, CEMB-*Cry*2A and CEMB-GTGene) in V_1_-generation. (**A**) Denaturing genomic DNA. (**B**) Southern DNA hybridization of transgenic sugarcane plants with CEMB-GTGene probe. **(C**) Southern DNA hybridization of transgenic sugarcane plants with CEMB-*Cry*1Ac probe. (**D**) Southern DNA hybridization of transgenic sugarcane plants with CEMB-*Cry*2A probe.

The transgenic sugarcane (CPF-246) lines 4L/2, 5L/5, 6L/5, L8/4, and L9/6 were screened from V_1_-generation (Fig. 13) on the basis of three selection criteria, (1) overall transgenes expressions (CEMB-*Cry*1Ac, CEMB-*Cry*2A and CEMB-GTGene, (2) leaf Bio-toxicity (mortality %age) and (3) Glyphosate herbicide spray assays, for the generation advancement as V_2_-generation in the field (Fig. 14). Five from 15 V_1_-generation free of any kind insect attack (Stem, Root and Top borers in the field) and completely tolerant to glyphosate spray were screened as V_2_-generation for further molecular analyses and field expression studies. The highest CEMB-*Cry*1Ac and CEMB-*Cry*2A expression was found 0.680 (µg/g) and 0.656 (µg/g) respectively in sugarcane transgenic line (4L/2-9) of V_2_-generation (Tables 5, 6, 7, 8, 9). The highest dose of CEMB-GTGene 0.595 (µg/g) was noted in line L6/5-4 from V_2_-generation. All the transgenic sugarcane lines (Table 10) showed 100% cane borer (*Chilo infuscatellus*) mortality and efficiently complete tolerance against glyphosate application 3000 mL/ha (Figs. 15, 16, Table 10).

**Table 5:** Molecular analysis of CPF-246 (L4/2-9) transgenic sugarcane plants from V_2_-generation.

Serial no	PCR			Southern Blot analysis			Dipstick assay			ELISA (µg/g)			Glyphosate spray assay (1200 m/acre)
Transgenic lines of sugarcane CPF-246	CEMB-*Cry*1Ac	CEMB-Cry2A	CEMB-GTGene	CEMB-Cry1Ac	CEMB-*Cry*2A	CEMB-GTGene	CEMB-*Cry*1Ac	CEMB-*Cry*2A	CEMB-GTGene	CEMB-*Cry*1Ac	CEMB-*Cry*2A	CEMB-GTGene	
CPF-246 (4L/2-9) 1	+VE	+VE	+VE	+VE	+VE	+VE	+VE	+VE	+VE	0.675	0.645	0.565	Tolerant
CPF-246 (4L/2-9) 2	+VE	+VE	+VE	+VE	+VE	+VE	+VE	+VE	+VE	0.677	0.641	0.561	Tolerant
CPF-246 (4L/2-9) 3	+VE	+VE	+VE	+VE	+VE	+VE	+VE	+VE	+VE	0.598	0.596	0.551	Tolerant
CPF-246 (4L/2-9) 4	+VE	+VE	+VE	+VE	+VE	+VE	+VE	+VE	+VE	0.645	0.644	0.560	Tolerant
CPF-246 (4L/2-9) 5	+VE	+VE	+VE	+VE	+VE	+VE	+VE	+VE	+VE	0.678	0.656	0.562	Tolerant
CPF-246 (4L/2-9) 6	+VE	+VE	+VE	+VE	+VE	+VE	+VE	+VE	+VE	0.670	0.651	0.567	Tolerant
CPF-246 (4L/2-9) 7	+VE	+VE	+VE	+VE	+VE	+VE	+VE	+VE	+VE	0.671	0.655	0.570	Tolerant
CPF-246 (4L/2-9) 8	+VE	+VE	+VE	+VE	+VE	+VE	+VE	+VE	+VE	0.612	0.634	0.559	Tolerant
CPF-246 (4L/2-9) 9	+VE	+VE	+VE	+VE	+VE	+VE	+VE	+VE	+VE	0.615	0.629	0.545	Tolerant
CPF-246 (4L/2-9) 10	+VE	+VE	+VE	+VE	+VE	+VE	+VE	+VE	+VE	0.679	0.642	0.568	Tolerant

**Table 6:** Molecular analysis of CPF-246 (L5/5-8) transgenic sugarcane plants from V_2_-generation.

Serial no	PCR			Southern Blot analysis			Dipstick asaay			ELISA (µg/g)			Glyphosate spray assay (1200 m/acre)
Transgenic lines of sugarcane CPF-246	CEMB-*Cry*1Ac	CEMB-Cry2A	CEMB-GTGene	CEMB-Cry1Ac	CEMB-*Cry*2A	CEMB-GTGene	CEMB-*Cry*1Ac	CEMB-*Cry*2A	CEMB-GTGene	CEMB-*Cry*1Ac	CEMB-*Cry*2A	CEMB-GTGene	
CPF-246 (5L/5-8) 1	+VE	+VE	+VE	+VE	+VE	+VE	+VE	+VE	+VE	0.680	0.598	0.577	Tolerant
CPF-246 (5L/5-8) 2	+VE	+VE	+VE	+VE	+VE	+VE	+VE	+VE	+VE	0.670	0.590	0.560	Tolerant
CPF-246 (5L/5-8) 3	+VE	+VE	+VE	+VE	+VE	+VE	+VE	+VE	+VE	0.676	0.595	0.575	Tolerant
CPF-246 (5L/5-8) 4	+VE	+VE	+VE	+VE	+VE	+VE	+VE	+VE	+VE	0.678	0.601	0.572	Tolerant
CPF-246 (5L/5-8) 5	+VE	+VE	+VE	+VE	+VE	+VE	+VE	+VE	+VE	0.674	0.596	0.580	Tolerant
CPF-246 (5L/5-8) 6	+VE	+VE	+VE	+VE	+VE	+VE	+VE	+VE	+VE	0.670	0.590	0.578	Tolerant
CPF-246 (5L/5-8) 7	+VE	+VE	+VE	+VE	+VE	+VE	+VE	+VE	+VE	0.665	0.580	0.581	Tolerant
CPF-246 (5L/5-8) 8	+VE	+VE	+VE	+VE	+VE	+VE	+VE	+VE	+VE	0.673	0.598	0.572	Tolerant
CPF-246 (5L/5-8) 9	+VE	+VE	+VE	+VE	+VE	+VE	+VE	+VE	+VE	0.675	0.591	0.570	Tolerant
CPF-246 (5L/5-8) 10	+VE	+VE	+VE	+VE	+VE	+VE	+VE	+VE	+VE	0.678	0.588	0.575	Tolerant

**Table 7:** Molecular analysis of CPF-246 (L6/5-4) transgenic sugarcane plants from V_2_-generation.

Serial no	PCR			Southern Blot analysis			Dipstick assay			ELISA (µg/g)			Glyphosate spray assay (1200 m/acre)
Transgenic lines of sugarcane CPF-246	CEMB-*Cry*1Ac	CEMB-Cry2A	CEMB-GTGene	CEMB-Cry1Ac	CEMB-*Cry*2A	CEMB-GTGene	CEMB-*Cry*1Ac	CEMB-*Cry*2A	CEMB-GTGene	CEMB-*Cry*1Ac	CEMB-*Cry*2A	CEMB-GTGene	
CPF-246 (6L/5-4) 1	+VE	+VE	+VE	+VE	+VE	+VE	+VE	+VE	+VE	0.685	0.605	0.589	Tolerant
CPF-246 (6L/5-4) 2	+VE	+VE	+VE	+VE	+VE	+VE	+VE	+VE	+VE	0.680	0.598	0.590	Tolerant
CPF-246 (6L/5-4) 3	+VE	+VE	+VE	+VE	+VE	+VE	+VE	+VE	+VE	0.681	0.596	0.585	Tolerant
CPF-246 (6L/5-4) 4	+VE	+VE	+VE	+VE	+VE	+VE	+VE	+VE	+VE	0.680	0.601	0.591	Tolerant
CPF-246 (6L/5-4) 5	+VE	+VE	+VE	+VE	+VE	+VE	+VE	+VE	+VE	0.682	0.591	0.595	Tolerant
CPF-246 (6L/5-4) 6	+VE	+VE	+VE	+VE	+VE	+VE	+VE	+VE	+VE	0.679	0.595	0.586	Tolerant
CPF-246 (6L/5-4) 7	+VE	+VE	+VE	+VE	+VE	+VE	+VE	+VE	+VE	0.680	0.599	0.591	Tolerant
CPF-246 (6L/5-4) 8	+VE	+VE	+VE	+VE	+VE	+VE	+VE	+VE	+VE	0.677	0.596	0.594	Tolerant
CPF-246 (6L/5-4) 9	+VE	+VE	+VE	+VE	+VE	+VE	+VE	+VE	+VE	0.621	0.610	0.588	Tolerant
CPF-246 (6L/5-4) 10	+VE	+VE	+VE	+VE	+VE	+VE	+VE	+VE	+VE	0.678	0.611	0.585	Tolerant

**Table 8:** Molecular analysis of CPF-246 (L8/4-3) transgenic sugarcane plants from V_2_-generation.

Serial no	PCR			Southern Blot analysis			Dipstick assay			ELISA (µg/g)			Glyphosate spray assay (1200 ml/acre)
Transgenic lines of sugarcane CPF-246	CEMB-*Cry*1Ac	CEMB-Cry2A	CEMB-GTGene	CEMB-Cry1Ac	CEMB-*Cry*2A	CEMB-GTGene	CEMB-*Cry*1Ac	CEMB-*Cry*2A	CEMB-GTGene	CEMB-*Cry*1Ac	CEMB-*Cry*2A	CEMB-GTGene	
CPF-246 (L8/4-3) 1	+VE	+VE	+VE	+VE	+VE	+VE	+VE	+VE	+VE	0.645	0.640	0.551	Tolerant
CPF-246 (L8/4-3) 2	+VE	+VE	+VE	+VE	+VE	+VE	+VE	+VE	+VE	0.640	0.644	0.542	Tolerant
CPF-246 (L8/4-3) 3	+VE	+VE	+VE	+VE	+VE	+VE	+VE	+VE	+VE	0.641	0.642	0.534	Tolerant
CPF-246 (L8/4-3) 4	+VE	+VE	+VE	+VE	+VE	+VE	+VE	+VE	+VE	0.640	0.622	0.538	Tolerant
CPF-246 (L8/4-3) 5	+VE	+VE	+VE	+VE	+VE	+VE	+VE	+VE	+VE	0.630	0.635	0.525	Tolerant
CPF-246 (L8/4-3) 6	+VE	+VE	+VE	+VE	+VE	+VE	+VE	+VE	+VE	0.638	0.635	0.535	Tolerant
CPF-246 (L8/4-3) 7	+VE	+VE	+VE	+VE	+VE	+VE	+VE	+VE	+VE	0.642	0.632	0.552	Tolerant
CPF-246 (L8/4-3) 8	+VE	+VE	+VE	+VE	+VE	+VE	+VE	+VE	+VE	0.635	0.638	0.551	Tolerant
CPF-246 (L8/4-3) 9	+VE	+VE	+VE	+VE	+VE	+VE	+VE	+VE	+VE	0.635	0.640	0.540	Tolerant
CPF-246 (L8/4) 10	+VE	+VE	+VE	+VE	+VE	+VE	+VE	+VE	+VE	0.641	0.646	0.550	Tolerant

**Table 9:** Molecular analysis of CPF-246 (L9/6-10) transgenic sugarcane plants from V_2_-generation.

Serial no	PCR			SOUTHERN			DIPSTICK			ELISA (µg/g)			Glyphosate spray assay (1200 m/acre)
Transgenic lines of sugarcane CPF-246	CEMB-*Cry*1Ac	CEMB-Cry2A	CEMB-GTGene	CEMB-Cry1Ac	CEMB-*Cry*2A	CEMB-GTGene	CEMB-*Cry*1Ac	CEMB-*Cry*2A	CEMB-GTGene	CEMB-*Cry*1Ac	CEMB-*Cry*2A	CEMB-GTGene	
CPF-246 (L9/6-10) 1	+VE	+VE	+VE	+VE	+VE	+VE	+VE	+VE	+VE	0.648	0.620	0.565	Tolerant
CPF-246 (L9/6-10) 2	+VE	+VE	+VE	+VE	+VE	+VE	+VE	+VE	+VE	0.646	0.611	0.561	Tolerant
CPF-246 (L9/6-10) 3	+VE	+VE	+VE	+VE	+VE	+VE	+VE	+VE	+VE	0.644	0.603	0.558	Tolerant
CPF-246 (L9/6-10) 4	+VE	+VE	+VE	+VE	+VE	+VE	+VE	+VE	+VE	0.650	0.599	0.555	Tolerant
CPF-246 (L9/6-10) 5	+VE	+VE	+VE	+VE	+VE	+VE	+VE	+VE	+VE	0.640	0.598	0.560	Tolerant
CPF-246 (L9/6-10) 6	+VE	+VE	+VE	+VE	+VE	+VE	+VE	+VE	+VE	0.645	0.599	0.559	Tolerant
CPF-246 (L9/6-10) 7	+VE	+VE	+VE	+VE	+VE	+VE	+VE	+VE	+VE	0.641	0.610	0.556	Tolerant
CPF-246 (L9/6-10) 8	+VE	+VE	+VE	+VE	+VE	+VE	+VE	+VE	+VE	0.639	0.596	0.567	Tolerant
CPF-246 (L9/6-10) 9	+VE	+VE	+VE	+VE	+VE	+VE	+VE	+VE	+VE	0.633	0.613	0.560	Tolerant
CPF-246 (L9/6-10) 10	+VE	+VE	+VE	+VE	+VE	+VE	+VE	+VE	+VE	0.640	0.615	0.562	Tolerant

**Table 10:** Level of pest control and dose assessment of CEMB-*Cry*1Ac, CEMB *Cry*2A (µg/g) and CEMB-GTGene (µg/g) from five transgenic sugarcane lines in V_2_-generation.

Serial no	Level of pest control and dose assessment at the plant age of 80 days			Glyphosate spray assay (3000 mL/hectare)	ELISA CEMB-GTGene dose assessment (µg/g)
Transgenic lines of sugarcane CPF-246	Mortality (%age)	ELISA			
		CEMB-*Cry*1Ac (µg/g)	CEMB-*Cry*2A (µg/g)		
1-CPF-246 (4L/2-9) 5	100%	0.678	0.656	Tolerant	0.562
2-CPF-246 (5L/5-8) 4	100%	0.678	0.601	Tolerant	0.572
3-CPF-246 (5L/5-8) 8	100%	0.673	0.598	Tolerant	0.572
4-CPF-246 (6L/5-4) 2	100%	0.680	0.598	Tolerant	0.590
5-CPF-246 (6L/5-4) 4	100%	0.680	0.601	Tolerant	0.591
6-CPF-246 (6L/5-4) 5	100%	0.682	0.591	Tolerant	0.595
7-CPF-246 (6L/5-4) 7	100%	0.680	0.599	Tolerant	0.591
8-CPF-246 (6L/5-4) 8	100%	0.677	0.596	Tolerant	0.594
9-CPF-246 (L8/4-3) 3	100%	0.641	0.642	Tolerant	0.534
10-CPF-246 (L8/4-3) 9	100%	0.635	0.640	Tolerant	0.540
11-CPF-246 (L8/4-3) 10	100%	0.641	0.646	Tolerant	0.550
12-CPF-246 (L9/6-10) 2	100%	0.646	0.611	Tolerant	0.561
13-CPF-246 (L9/6-10) 4	100%	0.650	0.599	Tolerant	0.555
14-CPF-246 (L9/6-10) 8	100%	0.639	0.596	Tolerant	0.567
15-CPF-246 (L9/6-10) 10	100%	0.640	0.615	Tolerant	0.562

### Dipstick assay

Dipstick assay confirmed the protein expression of three genes CEMB-*Cry*1Ac, CEMB-*Cry*2Ac and CEMB-GTGene in the lines of V_1_-generation. The bright dark color bands on the dipstick showed higher protein expressions in these lines CPF-246(4L/2-7) CPF-246(5L/5-2), CPF-246(6L/5-3), CPF-246(L8/4-10), and CPF-246 (L9/6-9) of sugarcane transgenic plants that verified the stability or stable expression of these transgenes into the genome of V_1_-geneartion.

### Enzyme linked immuno sorbent assay (ELISA)

PCR and Dipstick assay confirmed transgenic sugarcane plants were further evaluated by ELISA. ELISA was performed to check and quantify the level of protein expressions from CEMB-*Cry*1A, CEMB-*Cry*2A and CEMB-GTGene transgenes in the transgenic sugarcane leaves of V_1_-generation. Initially at randomly selected transgenic sugarcane (CPF-246) lines (2L/6)-3, (2L/6)-5, (3L/3)-8, (3L/4)-4, (4L/2)-7, (4L/7)-1, (4L/8)-4, (5L/1)-7, (5L/5)-2, (6L/2)-10, (6 6L/5)-3, (7L/2)-1, (7L/7)-7, (L8/4)-10, and (L9/6)-9 from field were analyzed for expression level of CEMB-*Cry*1Ac, CEMB-*Cry*2A and CEMB-GTGene genes. ELISA results demonstrated through plate reader Model-ELx800 confirmed that expressions of CEMB-*Cry*1Ac, CEMB-*Cry*2A and CEMB-GTGene proteins was much higher as compared to negative control and non transgenic sugarcane plants. Accordingly, V_1_-generation plants that were selected randomly showed almost stable quantities of these trans-proteins in plants (Table 4; Supplementary Figures 8–15). With reference to overall CEMB-*Cry*1Ac, CEMB-*Cry*2A and CEMB-GTGene proteins expression from all the transgenic lines, (4L/2)-7, (5L/5)-2, (6L/5)-3, (L8/4)-10, and (L9/6)-9 lines were distinct with respect to their quantified transproteins (Supplementary Figures 16, 17). These lines were highly insect resistant and glyphosate tolerant in their respective bioassays from V_1_ and V_2_-generations.

### Leaf bio-toxicity assay of transgenic sugarcane plants in V_1_-generation

**Figure 17:**
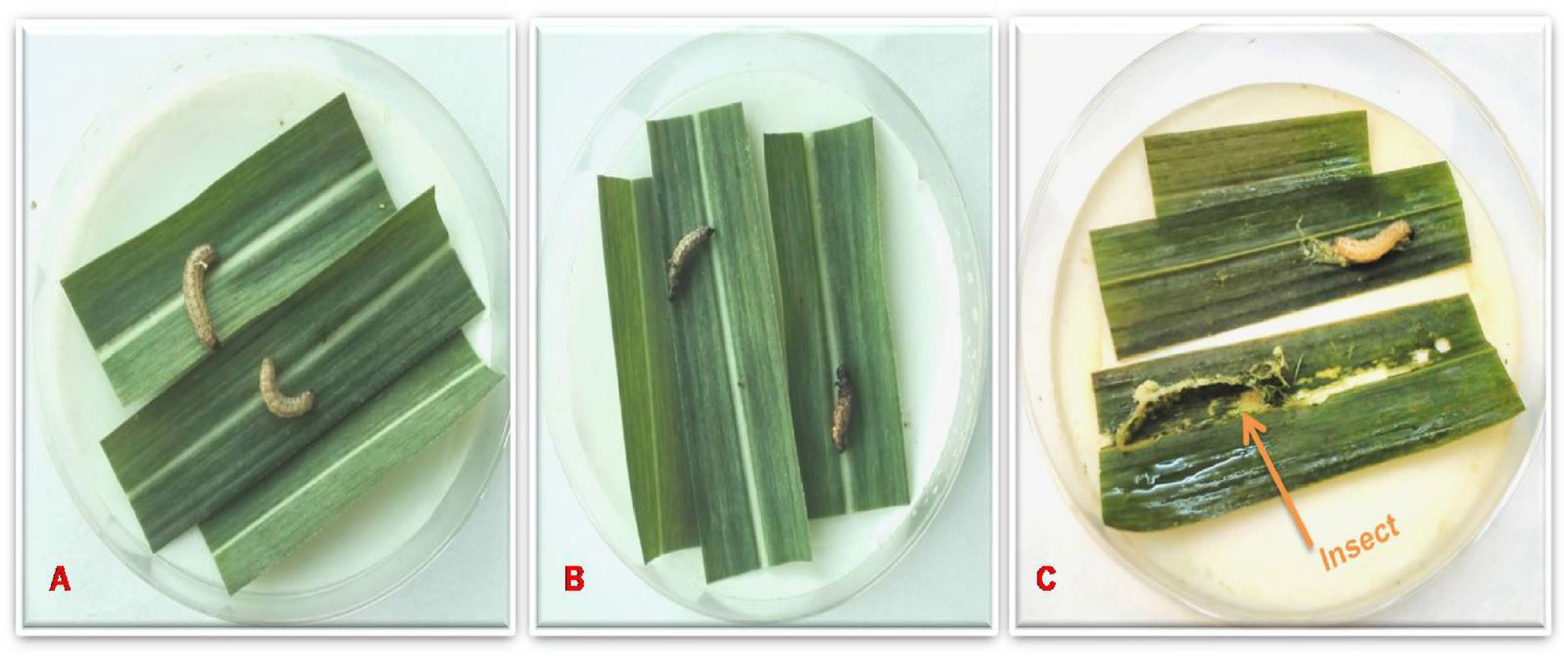
Leaf bioassay of transgenic sugarcane plant leaves and control plants in V_1_-generation. (**A**) Transgenic young leaf with *Chilo infuscatellus.* (**B**) Dead insects *Chilo infuscatellus.* (**C**) Control (non-transgenic plants) *Chilo infuscatellus* alived and healthy.

For further evaluation, these sugarcane transgenic lines were subjected to leaf bioassay against cane borer *Chilo infuscatellus* larvae (Fig. 17). Larvae of three different instars from *Chilo infuscatellus* were permitted, in three replicates, to feed on non-transgenic and transgenic plant leaves. The time intervals of 20, 40, 60 and 80 days were same as in-V_0_-generation after plantation in field soil. After 3 days of leaf bioassay, data was composed by counting the alive and dead insect larvae alongside assessing the leaf damages done by these larvae during this experimental time period (Supplementary material Table 9). Up to 70 to 100 (%) mortality of *Chilo infuscatellus* larvae was resulted from the expressed toxins of these transgenic sugarcane plants, according to variation in the toxins quantity which were observed from these lines during the course of time. Moreover, in control plates, the non transgenic leaves were totally damaged, eaten up and all the larvae were alived and healthy. Different statistical analyses such as Analysis of Variance (ANOVA), Least Significant Difference test (LSD) and Dunnett’s test were applied for toxicity-significance-analysis of transgenic sugarcane lines in comparison with non transgenic plant leaves. ANOVA showed that all the sugarcane transgenic lines significantly differ from the non transgenic plants and Dunnett’s (Supplementary material Table 9) results demonstrated that five (05) transgenic lines from V_1_-geneartion of CPF-246 are considerably differ from non-transgenic plants, as well as from other transgenic sugarcane lines (within the group) i.e. 4L/2, 5L/5,6L/5, L8/4 and L9/6 lines with respect to their toxicity expression against *Chilo Infuscatellus* insects.

## Discussion

High yielding is the ultimate objective of the crop production including sugarcane^1,35,42,43^. The major constraints to sugarcane are cane borer insects, weeds, drought stress and different viruses^2,5,44^. The present study was aimed to control the cane borers and weeds through the genetic modification of sugarcane with codon optimized insect resistant (CEMB-*Cry*1Ac + CEMB-*Cry*2A) and glyphosate tolerant genes (CEMB-GTGene).

The agro-climatic environment of the Pakistan is relatively encouraging for the sugarcane cultivation. In spite of worldwide area wise fifth position, Pakistan stands at fifteenth position in sugar production which is lowest yield per unit area. In monocots, a stable reproducible transformation method has been very important^45,46^ and in sugarcane^44,47^. In the current study, an effort was made for the development of an efficient procedure for its regeneration from calli and inbuilt resistance/tolerance in sugarcane transgenic plants against cane borers and glyphosate herbicide. For this purpose, four local elite varieties CPF-213, CPF-234, HSF-240, and CPF-246 were selected for establishing tissue culture experiments. Young immature leaves were used as explants for calli induction. Immature leaves were found excellent explants for embryogenic callus formation^48^, which is compulsory for genetic modification of sugarcane and also strengthen the genetic transformation procedure in the sugarcane^49^. The embryogenic calli of all the four varieties were obtained on a callus formation media containing 2, 4-D^50,51^. This media was supplemented with casein for the enhancement of embryogenic potential of sugarcane calli^52,53^. The calli were regenerated into micro-shoots, and after the multiplication of these micro-shoots, root intiation was started. These four varieties gave different responses for tissue culturing with reference to callogenesis and regeneration due to genotypic variability among cultivars^37,54^. It was also observed that increased concentration of 2,4-D, more than 4 mg/L, in callus induction media significantly adversely affected the regeneration efficiency of the calli. On increased concentrations, complete loss of the regeneration potential of these calli was noted. These results were consistent with the study by Chengalrayan et al.^55^, who described that fresh biomass of the calli reduced on adding upto 6 mg/L of 2,4-D in the media.

All the four varieties were critically judged/screened through tissue culture response^2,50^, and through transformation efficiency on the basis of calli survived (%) and calli regenerated (%) on selection media and finally through glyphosate field spray assays. After through screening, CPF-246 variety was selected for the next generation field studies. The results of tissue culture studies proved CPF-246 (callus induction 90%, regeneration 99% and multiplication 100%) as an excellent variety. Bakhsh et al.^56^ also selected varieties for genetic modification by means of regeneration response. Crop genetic modification through the introduction of insecticidal (*Cry*1Ac, *Cry*2A) and glyphosate tolerant (GTG) genes is more advantageous over conventional breeding techniques^57^. The transgenic sugarcane provides resistance against the cane borer pests with *Bt.* genes^5,58^. More than 100 *Bt*. genes have been sequenced which were highly dissimilar in amino-acid sequence and in their few biochemical properties as well. Studies proved that in these toxins’ combination, *Cry*1Ac with *Cry*2A is better against borer insects or *lepidopteran* insects^59,60^. The introduction of double *Bt*. genes *Cry*1Ac and *Cry*2A also represents the gene pyramiding which helps in creating multiple traits in one variety^61^.

Presently, a broad spectrum herbicide, glyphosate is the most commonly used herbicide. Glyphosate is a non-selective- herbicide that can stop the growth of all plants along with a wide range of herbs and weeds^62^. Glyphosate inhibits the synthesis of 5-enolpyuvyl-3-phosphoshikimate (EPSPS) during the shikimate metabolic pathway and subsequently stops the synthesizing of three very important amino acid tyrosine, tryptophan and phenylalanine^63^. Many crops for herbicide resistance has been developed formerly, including the non-glyphosate herbicide-tolerant transgenics with 2,4-D, Dicamba, HPPD inhibitors and bar transgenes^64,65^. However, all these crops were resistant against specific kind of herbicide, while transgenics with c*p*4EPSPS are not confined with single kind of herbicid^27,66^. Up till now, nine glyphosate-resistant crops have been introduced, comprising soybean, wheat, canola, polish canola, corn, alfalfa, sugar beet, creeping bent-grass and cotton^17,37^.

The modified sequences of CEMB for *Bt.* (CEMB-*Cry*1Ac + CEMB-*Cry*2A) and glyphosate tolerant (c*p*4EPSPS) genes were used for this transformation study. The nuclear encoded chimeric genes were used in preliminary efforts for the expression of *Bt.* toxins but the resulted expression level was very low^67^. It was supposed that *Bt.* genes are AT-rich in comparison with plant genes and therefore led to the consideration that the reasons for low expression can be termination of pre-mature transcription, abnormal mRNA splicing, instable mRNA or incompetent codon usage^68^. Synthetic *Bt.* toxin genes were created (designed), constructed and cloned to neglect all these undesirable aspects, and the resulted expression of these transgenes in plants was significantly enhanced^69,70^. This jointly with experimenting a variety of promoters and other sequences, led to a considerable development of insect borer resistance and improvement in expressing the toxin levels 0.8% of the overall leaf protein^71^.

In the current study we have constructed and introduced three codon optimized synthetic genes named as CEMB-*Cry*1Ac + CEMB-*Cry*2A and CEMB-GTGene in the nuclear genome of sugarcane. The expression of these synthetic genes is under the control of maize Ubiquition-1 promoter^37^. Simple and minimal plasmid cassettes were constructed for maximum protein expression^72,73^. A study was made on comparison between expression of *Bt*. and GTG genes through two different promoters CAMV35S and Maize Ubiquitin-1 (data not included). The resulted expression under Ubiquitin promoter confirmed higher toxin levels^70^. So maize Ubiquitin promoter was used to enhance the expression of these transgenes and improved increased resistance against cane bores and tolerance for glyphosate herbicide. After transformation a number of transgenic sugarcane plants were regenerated from transformed calli using this plant expression constructs (*p*CEMB-SC12) for CEMB-*Cry*1Ac + CEMB-*Cry*2A and *p*CEMB-SGTG for glyphosate tolerant gene (CEMB-GTGene).

In this present study the stability and inheritance of three transgenes up to three vegetative generations (V_0_, V_1_, and V_2_) verified that this technique of transformation can be used efficiently^2^. From 100 transformed calli, 81% was survived on the selection pressure (Kanamycine 50 mg/L) while 48% transformed calli were regenerated on double selection medium (Kanamycine 50 mg/L + glyphosate 50 mM). From 1000 resistant multiplied plants (CPF-246), 15 plants were positive for CEMB-*Cry*1Ac, CEMB-*Cry*2A and CEMB-GTGene, resulting transformation efficiency 1.5% after optimized glyphosate spray assay (1200 mL/80 L/acre). In monocots, the transformation efficiency was reported from 1 to 5%^30^ while co-integration of combine two genes was 85% when a combination of 14 diverse (pUC- based) plasmids was transformed^74^. Joyce et al.^52^ reported 0.8–4.8% transformation efficiency in sugarcane.

From these, 15 putative transgenic plants positive for three genes CEMB-*Cry*1Ac, CEMB-*Cry*2A and CEMB-GTGene were obtained. Molecular analyses of putative transgenic plants were carried out, first for their screening and then for their stable integration and expression level of transformed transgenes. Transgenic plants obtained on the basis of selection (kanamycine 50 mg/L) were considered as putative plants. The GUS assay revealed that activity of this gene is observable in all parts of the plants. The leaf and stem segments from the GUS positive plants were examined under fluorescent microscope and uniformly distributed expression was observed. The linked genes with 100% co-integration frequency are already reported^75^. Chen et al.^74^ also described the successful co-transformation with 14 different transgenes in rice. PCR analysis was carried out for the screening of putative transgenic plants selected on Kanamycine and glyphosate selection media. At V_0_-generation, the fifteen (15) screened plants were positive for CEMB-*Cry*1AC + CEMB-*Cry*2A and CEMB-GTGene in PCR amplification through gene specific primers.

The integration studies of these three transgenes in the genome of the sugarcane were confirmed by Southern DNA hybridization. Both single and double restriction digestion of the plasmids *p*CEMB-SC12 and *p*CEMB-SGTG was performed to digest the full cassettes and for having an estimation of the copy numbers. In current study 1–2 copy numbers were found. There are many reports for 1–5 copy numbers of transgenes introduced into crop plants by gene gun method in monocots^76,77^. For V_1_-generation, most of the time there was a co-relation between *Cry*1Ac and *Cry*2A banding pattern and intensity of the hybridizing bands, indicative of similar copy numbers of the transgenes. This recommends that the integrated plasmids, in general, integrate as a whole unit^77^. There were no rearrangements detections for these transgenes, Southern DNA hybridization confirming the integration of intact transgenes copies. Komari^78^ reported 1–6 copies of integrated genes while majority of the transgenics consist of 1–2 copies.

Transprotein expression levels were confirmed by the ELISA and Dipstick assay. The results of Dipstick-ELISA assay or immuno-Strip assay and quantification through DAS-ELISA confirmed that all of the 15 clones (V_0_) were consisting of CEMB-*Bt*. and CEMB-GTGene genes, although the expression levels of these transgenes were variable^79^. There might be many reasons for this, such as variability in transgene copy number^80^, transgene insertion locations in the genome^8^ or heterozygosity of the sugarcane transgenic lines, internal as well as external environment^81^. Koncz et al.^82^ and Zambryski et al.^83^ also reported that in transgenic plants, foreign DNA can integrate randomly on the chromosomal sites in the cells of plants through non-homologous recombination. The highest transprotein expression of CEMB-*Cry*1Ac, CEMB-*Cry*2A and CEMB-GTGene were observed as 0.475 µg/g, 0.567 µg/g, and 0.486 µg/g respectively. Overall ELISA based comparison CPF-246-(5L/5), CPF-246-(6L/5) were highest in their three expressions and CPF-246 (2L/8) and CPF-246 (5L/1) lines were lowest. The clones of V_0_-generation multiplied with 150 replicates (V_1_-generation) in the field showed positive results for PCR amplification through gene specific primers. Fifteen plants which were randomly selected from V1 and V_2_-generation showed stability for the integration and in protein expressions of CEMB-*Cry*1Ac, CEMB-*Cry*2Ac and CEMB-GTGene. The highest transprotein expressions of CEMB-*Cry*1Ac, CEMB-*Cry*2A and CEMB-GTGene were observed as 0.687 µg/g, 0.611 µg/g, and 0.589 µg/g respectively. Overall ELISA based comparison CPF-246-(5L/5), CPF-246-(6L/5) were highest in their three expressions and CPF-246-(2L/8) and CPF-246 (5L/1) lines were lowest. The stable integration of these transgenes was confirmed through Southern Blot analysis. These results showed similarity to those achieved by Bashir et al.^84^, for transgenic Basmati rice. These results were similar to those obtained by Kiani et al*.*^85^ while studying the expression of *Cry*1Ac protein through ELISA in transgenic cotton plants. Deng et al.^86^ observed similar quantified values in the transgenic lines of rice. In V_2_-generation, screened five transgenic sugarcane lines as 50 clones were multiplied in the field. Molecular analyses of three transgenes confirmed its stable trans-protein expression. The clones of these five lines were positive for all the three transgenes by PCR amplification, Southern Blotting, Dipstick assay and ELISA. All the sugarcane clones in this (3rd) generation (V_2_) were stable in their expressions^87^. The glyphosate tolerant gene expression was stable with respect to their ELISA protein quantification values and as well as by spray assays. The results showed similarity to those found by Leibbrandt et al.^88^ and Manickavasagam et al.^89^. Inheritance of integrated genes was studied stable in these three generations. The transprotein of integrated genes in V_0_, V_1_, and V_2_-generations further verified through insect (*Chilo infuscatellus*) bioassay.

### Bio-toxicity leaf assay

Leaf bio-assay was carried out to check the efficacy of transgenes (CEMB-*Cry*1Ac, CEMB-*Cry*2A and CEMB-GTGene) proteins against *Chilo infuscatellus* (2nd instar larvae) shoot borer at four different time intervals. The expressional level of toxins from these genes is significantly imprtant as it should be in such sufficient quantitative level at the infestation time that can protect the crop against attack of shoot borers of *Lepidopteran.* In V_0_-generation, with the passage of time the toxin levels of CEMB-*Cry*lAc and CEMB-*Cry*2A were decreased and percentage of cane borer damage increased, as the toxin level dropped from 0.475 to 0.252 µg/g for CEMB-*Cry*1Ac and 0.467 to 0.278 µg/g for CEMB-*Cry*2A, but all the transgenic lines showed significant insect mortality (60–100%) in comparison with control plants^90^. In V_1_-generation 15 lines with 150 clones were analyzed for leaf bioassay, each transgenic line was significantly differed from non-transgenic plants and performed better against in leaf bio-toxicity assays. Five lines were stable in their transproteins (CEMB-*Cry*1Ac and CEMB-*Cry*2A) expression and were also free of any kind of insect attack in the field where as control plants were totally damaged. In bioassay less damage to transgenic plant and complete consumption of non-transgenic plants verified strong defense of transgenic sugarcane against cane borer (*Chilo infuscatellus*). Similar results were published against borers^5,8,91^.

Analysis of Variance (ANOVA), the Least Significant Difference Test (LSD) and Dunnett’s Test confirmed the significant differences for *Chilo infuscatellus* mortality (%) between the control and transgenic sugarcane plants. ANOVA and Dunnett’s test verified highly significant difference at 5% level of significance for transgenic sugarcane line with respect to *Chilo infuscattelus* mortality. In V_2_-generation insect mortality was highly stable (90%-100). LSD analysis illustrated that transgenic leaf samples from CPF-246 (6L/5-4) at 20-days produced significantly high *Chilo infuscatellus* larvae mortality percentage (88%) compared with transgenic line (5L/1-9), which showed lowest mortality percentage value (66%). In general this data revealed the significant leaf damage and insect mortality (%) differences in control and transgenic plants respectively and also supported the conclusions of Riaz et al.^2^; Weng et al.^8^; Manikandan^58^.

Success of transformation studies depends upon the integration of the desired genes in the genome of the desired plants in addition to its inheritance to the next generation plants. Inheritance of stably integrated genes was studied up to three vegetative generations. Results acquired from PCR, Southern Blot analysis, Dipstick and ELISA (V_0_-generation) clearly confirmed the amplification and integration of three genes in the genome of sugarcane plants. Mainly the Southern Blot analysis of V_1_-generation indicated that two copies of transformed genes were present in the genome of sugarcane plants. ELISA analysis, on the other hand also confirmed the actively integrated genes were efficiently transcribed into protein as toxin. The transprotein of integrated genes in V_0_, V_1_ and V_2_ generation were verified through insect (*Chilo infuscatellus*) bioassay.

### Herbicide spray assay

Weeds affect by limiting the available nutrients to the major cash crops^92^. When glyphosate (3000 mL/ha) sprayed on the transgenic sugarcane plants, after 15 days 160 out of 300 plants was tolerant to glyphosate stress. The rest of the field plants showed necrotic symptoms, expired tissues and weeds were totally dead after this spray application. In the V_1_-generation, 75% of plants were alived and tolerant for glyphosate (3000 mL/ha) while 25 percent non tolerant plants were observed dead in some lines. The low expression may be due to inner or outer environmental conditions. In the V_2_-generation, ten clone plants from each of five transgenic (CPF-246) lines 4L/2, 5L/5, 6L/5, L8/4, and L9/6 were tested. Such comparable results were formerly reported by Joyce et al.^52^. In V_2_-generation all the lines with their clone replicates were tolerant to glyphosate spray (3000 ml/80 L/hectare). Finally, CPF-246 produced five tolerant lines with CEMB glyphosate tolerant gene. These results are in accordance with the previous studies^37,93–95^. It can be concluded that CEMB-*Cry*1Ac, CEMB-*Cry*2A and CEMB-GTG genes successfully introduced into the genome of the sugarcane CPF-246. Stable expression and insecticidal activity of transgenic plants was confirmed in V_0_, V_1_ and V_2_-generations. Molecular analyses confirmed the expression level of toxin in the transgenic lines of V_0_, V_1_ and V_2_ generations. Transformation of sugarcane with two *Bt.* and GTGene will open new directions for the development of a high yielding cane borer resistant and glyphosate tolerant sugarcane. These transgenic lines can be further improved with other high sugar yielding characters. The end product can be a transgenic variety with a number of beneficial traits. There is also an international inclination towards economically safe, ecologically and environmentally friendly methods which can enhance the defense mechanism of the crop against pest pathogens^96,97^.

Biological insect control methods by using naturally available bacteria, fungi, and viruses received limited acceptance from the users, as their capability to defend plants has commonly been substandard to results achieved by chemical ways. Insecticides have been relatively successful in increasing crop production by minimize the losses rooted by insects, but the use of potentially dangerous chemical sprays and pesticides is disfavored in several countries and even a few compounds deregistered, secondly chemical sprays are not acceptable in sugarcane. The most effectual way of achieving high crop production is to set up the desired resistance into the crop plant by genetic transformation. Now it’s possible to create plants that are resistant to insect borers or having multiple novel disease resistant genes which are modified according to the crop genome and adapted for specific soil or environmental conditions.

## Conclusion

The five transgenic sugarcane lines (4L/2, 5L/5, 6L/5, L8/4, L9/6) from CPF-246 variety harboring codon optimized CEMB-*Cry*1Ac, CEMB-*Cry*2A and CEMB-GTGene have revealed excellent potential up to 100% mortality of *Chilo infuscattellus* larvae (*Lepidopteran*) at the plant age of 80 days along with complete weed removal on 3000 mL/ha glyphosate tolerance level. This study concludes that if approved by the biosafety committee, this transgenic sugarcane can be a good starting material for the farmer’s community for the cost effective control of insects and weeds. Further studies are recommended to increase the stable expression of *Bt.* toxins up to the maturity, which can be achieved by further modification of the gene constructs. It has also been reported that glyphosate crops with tolerance level up to 5000 mL/ha might be more useful to control all types of sugarcane weeds in all the agroclimatic regions of the country. To achieve the above goals new vegetative genes such as Vip3A and Vip3B from *Bt.* in combination with CEMB-*Cry*1Ac and CEMB-*Cry*2A can be more effective, with different enhancers, promoters etc. can be developed for further strengthening/safeguarding this industrial cash crop from the insects and weeds**.**

## Supplementary Information

Supplementary Information.
