# Supplementary material for: Novel approaches to circumvent the devastating effects of pests on sugarcane
Source: Sci Rep. 2021 Jun 14;11:12428. doi: 10.1038/s41598-021-91985-8 (PMC8203629; doi:10.1038/s41598-021-91985-8)
Supplement: Supplementary file 1 — Supplementary Information. [file 41598_2021_91985_MOESM1_ESM.docx]

# Supplementary Materials


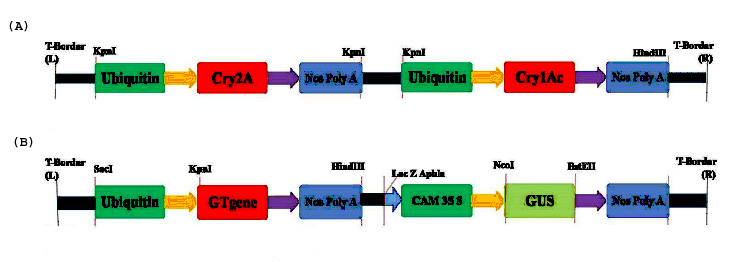


Supplementary Figure No.1: Physical as well as restriction map of chemically sized gene cassettes; (A): Cassette of CEMB-Cry2A and CEMB-Cry1Ac. (B): Cassette of CEMB-GTGene.

*Supplementary Table.1: Sequences of primers used for PCR amplification of CEMB-Cry1Ac, CEMB-Cry2A and CEMB-GTGene.*

| **Primer ID** | **Sequence (5’-3’)** |
| --- | --- |
| CEMB-*Cry*1Ac-F | GTTCTGCCCCAAGGTATCGAA |
| CEMB-*Cry*1Ac-R | GGACATTGTTGTTCTGTGG |
| CEMB-*Cry*2A-F | GAAGGAGTGGATGGAGTGGA |
| CEMB-*Cry*2A-R | GCGGTCTGGTAGGTGTTGAT |
| CEMB-GTGene-F | GGCCAGATCTATGTCCCACGGTGCTTCT |
| CEMB-GTGene-R | GGCCAGATCTCTAAGCAGCCTTAGCCTTAGTGTCGGAGA |

**Table 2: SphI enzyme for restriction digestion**

| **Reagents** |  | **Quantity** |
| --- | --- | --- |
| *p*UC construct | | 100ug |
| Tango Buffer |  | 2µL |
| SphI(10U/uL) |  | 1µL |
| H_2_O | | 3µL |
| Total volume | | 20µL |

### Table 3: Restriction Endonuclease Digestion and Ligation

| **Reagents** |  | **Quantity** |
| --- | --- | --- |
| *p*UCvector | | 1ug |
| Kpn1(10U) |  | 1µl |
| HindIII(10U) |  | 1µl |
| Tango buffer | | 2µl |
| H_2_O | | 5µl |
| Total volume | | 20µl |

For the construction/confirmation of plant expression vector *p*CEMB-SGTG the reaction mixture was as

| **Reagents** |  | **Quantity** |
| --- | --- | --- |
| *p*UCvector | | 1ug |
| Sac1(10U) |  | 1µL |
| HindIII(10U) |  | 1µL |
| Tango buffer | | 2µL |
| H_2_O | | 5µL |
| Total volume | | 20µL |

Enzymes and Reagents

Restriction enzymes of Fermentas and reagents of Sigma Company were used.


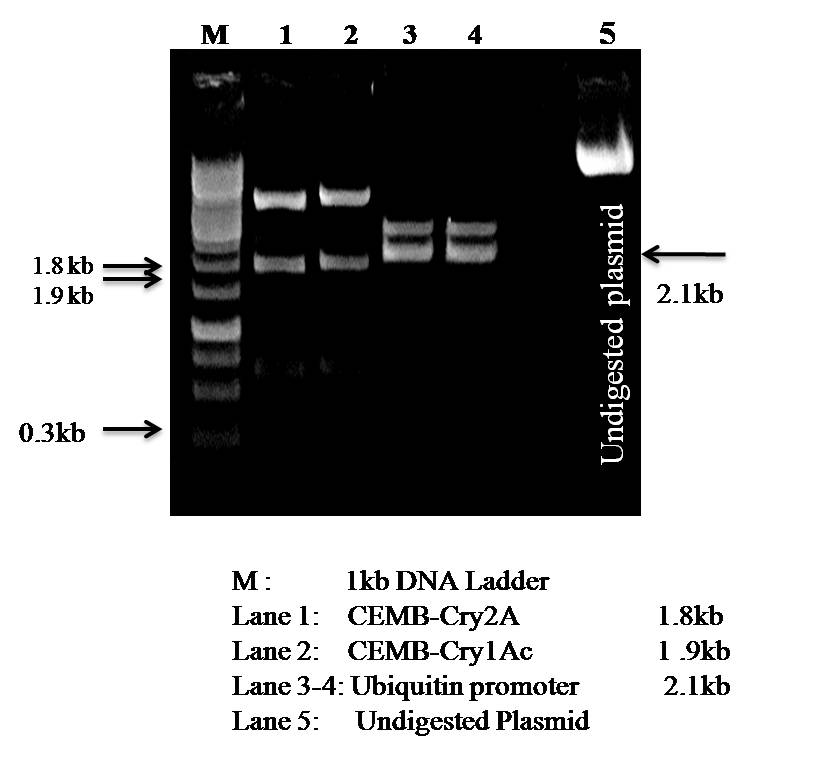


**Figure 2**: Restriction Digestion analysis for the confirmation of CEMB-*Cry*1Ac and CEMB-*Cry*2A

**Lane M:**  1kb DNA Ladder

**Lane 1:** CEMB-*Cry*2A 1.8kb

**Lane 2:** CEMB-*Cry*1Ac 1.9kb

**Lane 3-4:** Ubiquitin promoter 2.1kb

**Lane 3-4:** Undigested Plasmid constructs (*p*CEMB-SC12)


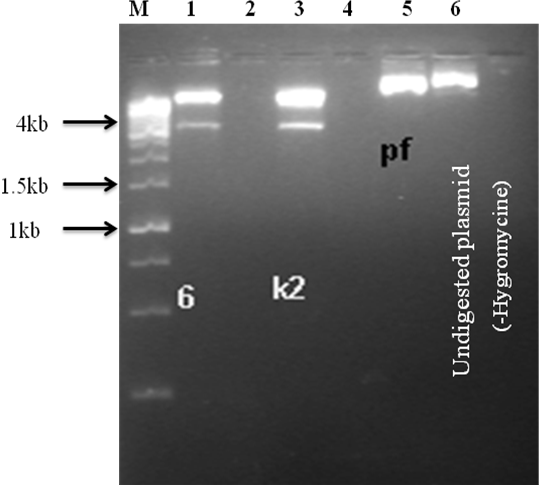


Figure 3A: Confirmation of cloned expression cassettes in final expression vector (*p*CEMB-SC12) through Restriction Digestion analysis

**Lane 1:** 1kb DNA Ladder

**Lane 2:** Restricted construct cassette Ubi- CEMB *Cry*2Ac-NOS (4kb)

**Lane 3:** Restricted construct cassette Ubi- CEMB *Cry*1Ac-NOS (4kb)

**Lane 5-6:** Undigested plant plasmid constructs (*p*CEMB-SC12)


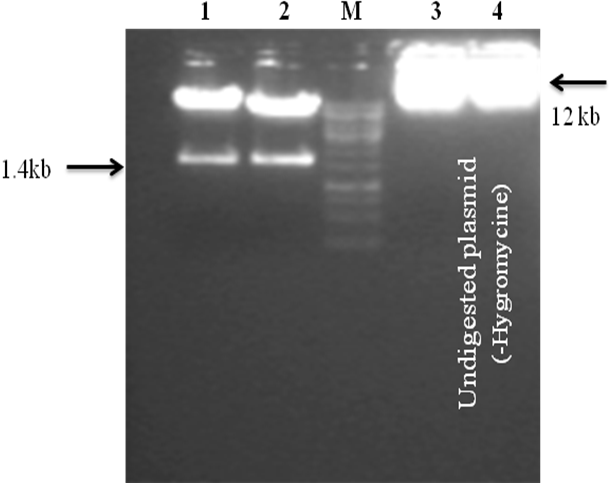


Figure 3B: Confirmation of cloned CEMB-GTG through Restriction Digestion analysis

**Lane M:** 1kb DNA Ladder

**Lane 1-2:** Double Digestion CEMB-GTGene (1.4kb)

**Lane 3-4:** Undigested plant plasmid constructs (*p*CEMB-SGTG)


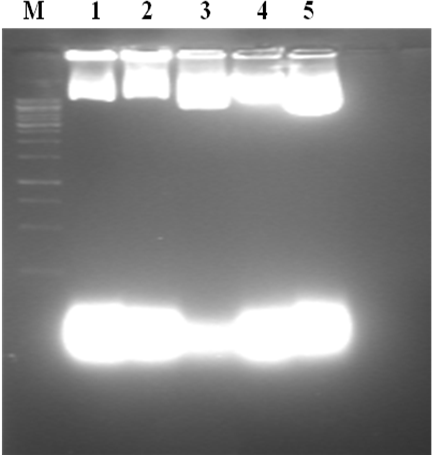


Figure 3C: Maxi-prep for the transformation in sugarcane through Biolistic method

**Lane M**: 1kb DNA Ladder

**Lane 1-2:** Plasmid Construct *p*CEMB-SGTG

**Lane 3-5:** Plasmid Construct *p*CEMB-SC12


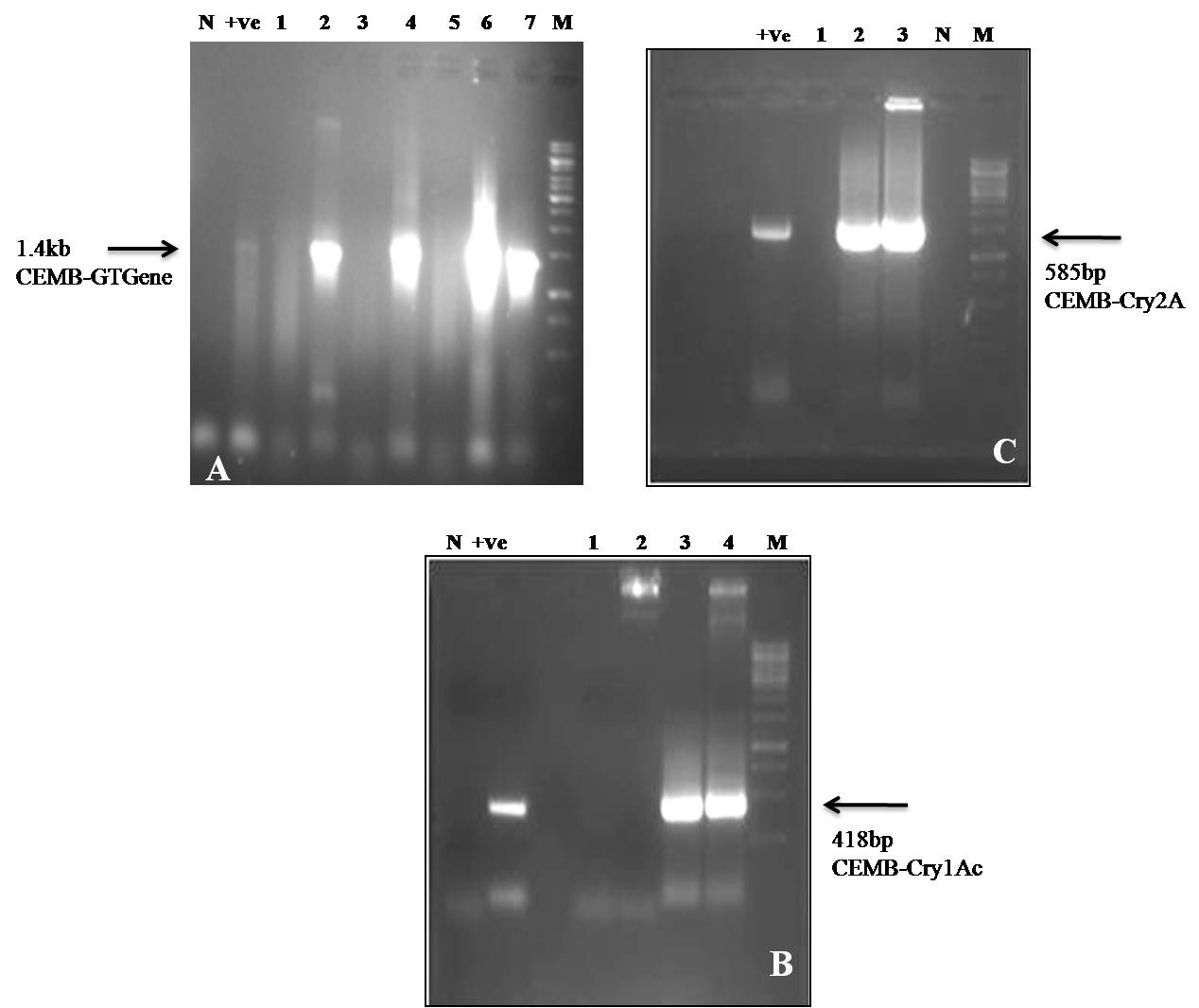


Figure 4: PCR amplification of three genes positive transformant from *Agrobacterium* electroporated with *p*CEMB-SC12 and *p*CEMB-SGTG constructs

| **A**)  M: 1kb DNA Ladder  Lane: 1-7:1.4 kb CEMB-GTGene  (Screening of positive colonies)  +Ve : Positive colony  N: Negative control | **B**)  M: 1kb DNA Ladder  Lane: 1-3: 41bp CEMB-*Cry*1Ac  (Screening of positive colonies)  +Ve : Positive colony  N: Negative control | **C**)  M: 100bp DNA Ladder  Lane: 1-4:485 bp CEMB-*Cry*2A  (Screening of positive colonies)  +Ve : Positive colony  N: Negative control |
| --- | --- | --- |


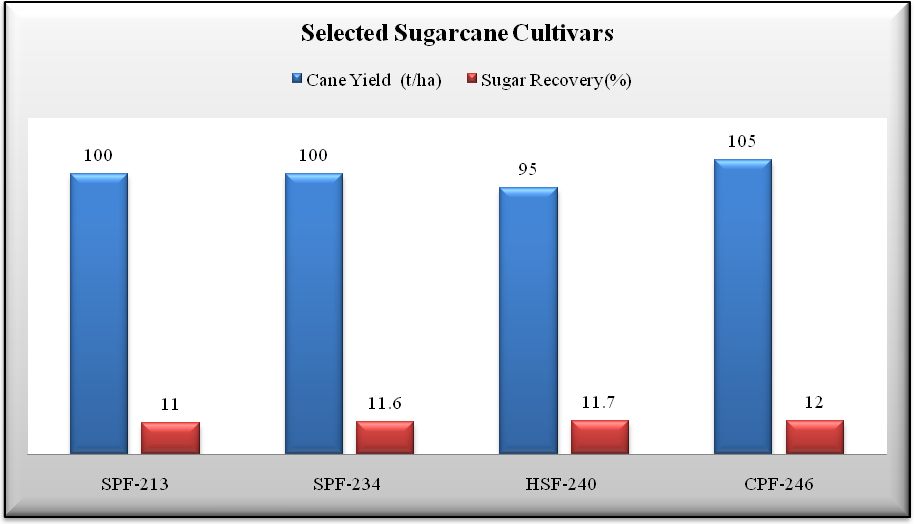


Figure 5: Four selected varities with cane yield (t/ha) and sugar recovery (%) for three genes transformation in sugarcane (*Saccharum officinarum* L.)


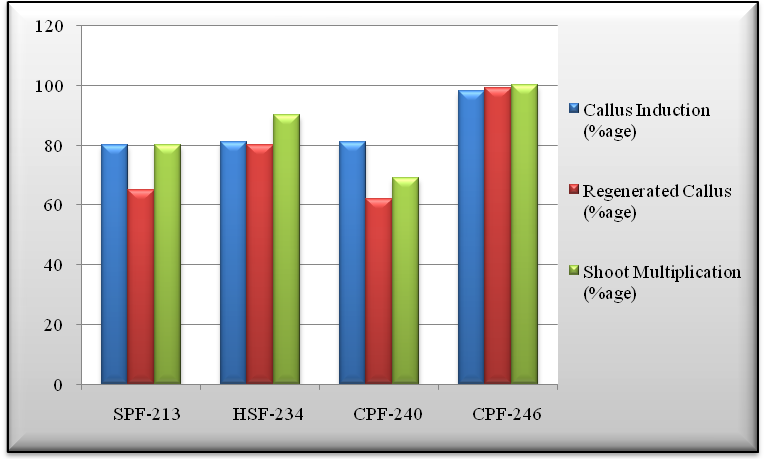


**Figure 6:** Callus induction, regeneration and shoot multiplication responses of four sugarcane varieties during tissue culturing

Table 4: Transformation efficiency on the basis of calli survived on selection media

| **Variety** | **No. of calli bombarded** | **Control** | **No. of calli survived on selection media (%)** | **Transformation efficiency (%)** |
| --- | --- | --- | --- | --- |
| **SPF-213** | 100 | 10 | 34 | 34% |
| **SPF-234** | 100 | 10 | 40 | 40% |
| **HSF-240** | 100 | 10 | 29 | 29% |
| **CPF-246** | 100 | 10 | 81 | 81% |

Table 5: Transformation efficiency on the basis of calli regenerated on selection media

| **Variety** | **No. of calli bombarded (%)** | **Control** | **No. of calli regenerated on selection media (%)** | **Transformation efficiency (%)** |
| --- | --- | --- | --- | --- |
| **SPF-213** | 100 | 10 | 21 | 21% |
| **SPF-234** | 100 | 10 | 32 | 32% |
| **HSF-240** | 100 | 10 | 13 | 13% |
| **CPF-246** | 100 | 10 | 48 | 48% |

Table 6: Estimation of Total Crude Protein in Putative Transgenic Plants by Bradford Assay

| **Serial No.** | **Plant Name** | **Concentration of Protein (µg/mL)** |
| --- | --- | --- |
| **1** | CPF-246 (2L/6) | 220 |
| **2** | CPF-246 (2L/8) | 280 |
| **3** | CPF-246 (3L/3) | 190 |
| **4** | CPF-246 (3L/4) | 160 |
| **5** | CPF-246 (4L/2) | 230 |
| **6** | CPF-246 (4L/7) | 180 |
| **7** | CPF-246 (4L/8) | 160 |
| **8** | CPF-246 (5L/1) | 190 |
| **9** | CPF-246 (5L/5) | 220 |
| **10** | CPF-246 (6L/2) | 170 |
| **11** | CPF-246 (6L/5) | 210 |
| **12** | CPF-246 (7L/2) | 180 |
| **13** | CPF-246 (7L/7) | 170 |
| **14** | CPF-246 (L8/4) | 230 |
| **15** | CPF-246 (L9/6) | 200 |


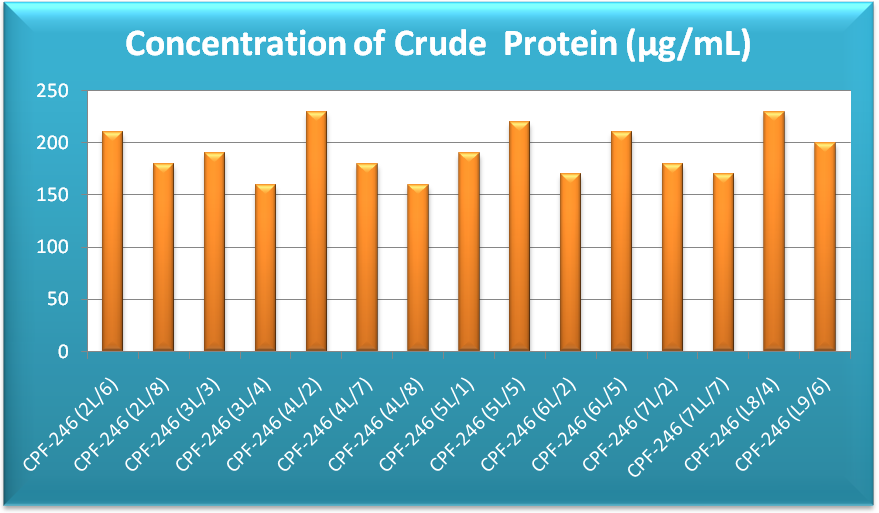


Figure 7: Estimation of total crude protein in putative transgenic plants by the Bradford Assay

Table 7: Quantification of CEMB-*Cry*1Ac, CEMB-*Cry*2A and CEMB- GTGene proteins from sugarcane plants of V_0_-Generation

| Plants Name | Protein (Conc.ug/g) CEMB-*Cry*1Ac | Protein (Conc.ug/g) CEMB-*Cry*2Ac | Protein (Conc.ug/g)  CEMB-GTGene |
| --- | --- | --- | --- |
| CPF-246 (2L/6) | 0.415 | 0.414 | 0.352 |
| CPF-246 (2L/8) | 0.360 | 0.278 | 0.320 |
| CPF-246 (3L/3) | 0.431 | 0.345 | 0.322 |
| CPF-246 (3L/4) | 0.356 | 0.401 | 0.425 |
| CPF-246 (4L/2) | 0.460 | 0.430 | 0.436 |
| CPF-246 (4L/7) | 0.341 | 0.341 | 0.334 |
| CPF-246 (4L/8) | 0.426 | 0.430 | 0.327 |
| CPF-246 (5L/1) | 0.252 | 0.322 | 0.348 |
| CPF-246 (5L/5) | 0.447 | 0.567 | 0.480 |
| CPF-246 (6L/2) | 0.351 | 0.400 | 0.386 |
| CPF-246 (6L/5) | 0.451 | 0.511 | 0.478 |
| CPF-246 (7L/2) | 0.382 | 0.323 | 0.331 |
| CPF-246 (7LL/7) | 0.386 | 0.360 | 0.435 |
| CPF-246 (L8/4) | 0.456 | 0.480 | 0.486 |
| CPF-246 (L9/6) | 0.475 | 0.490 | 0.447 |
| Negative control | 0.00 | 0.00 | 0.00 |
| Positive control | 0.058 | 0.032 | 0.056 |


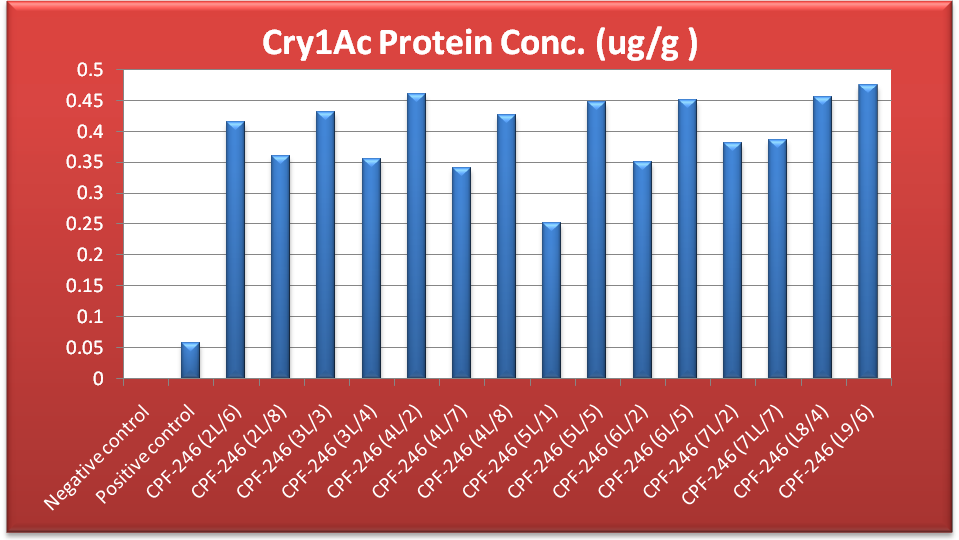


Figure 8: ELISA Quantification of CEMB-*Cry*1Ac transprotein of transgenic sugarcane plants in V_0_-generation

Lane 1: Control sugarcane plants Lane 2: Positive control, Lane 3-17: Sugarcane transgenic plants


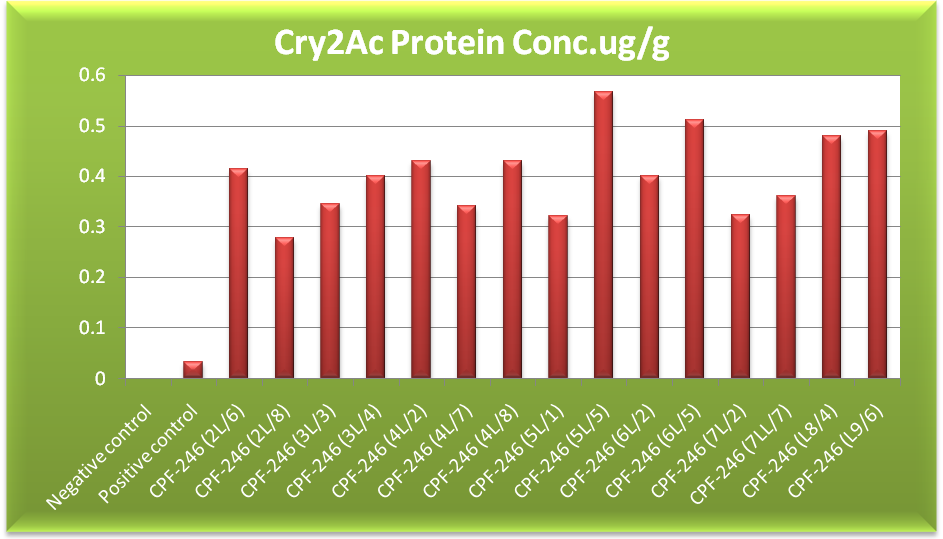


Figure 9: ELISA Quantification of CEMB-*Cry*2A transprotein of transgenic sugarcane plants in V_0_-generation

Lane 1: Control sugarcane plants Lane 2: Positive control Lane 3-17: Sugarcane transgenic plants


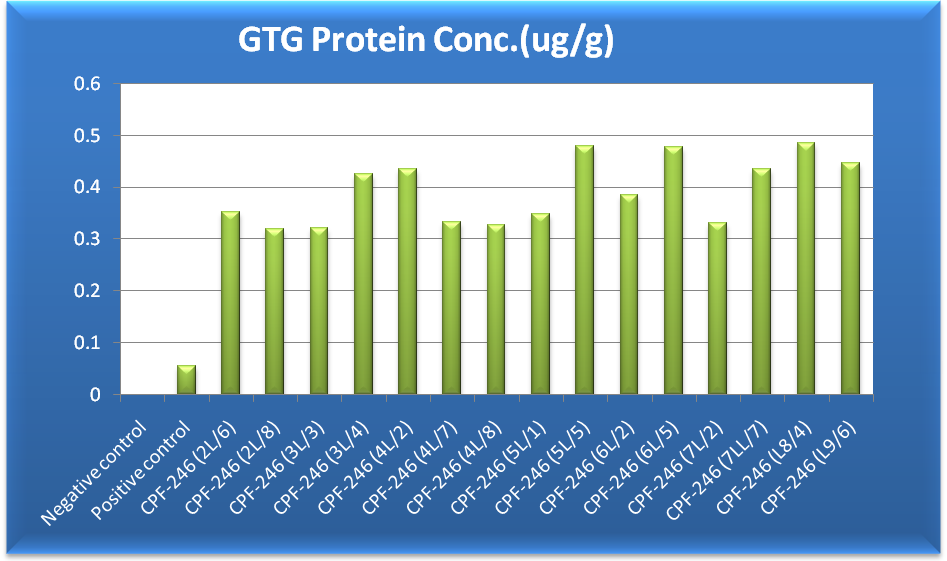


Figure 10: ELISA Quantification of CEMB-GTGene transprotein of transgenic sugarcane plants in V_0_-generation

Lane 1: Control sugarcane plants Lane 2: Positive control Lane 3-17: Sugarcane transgenic plants


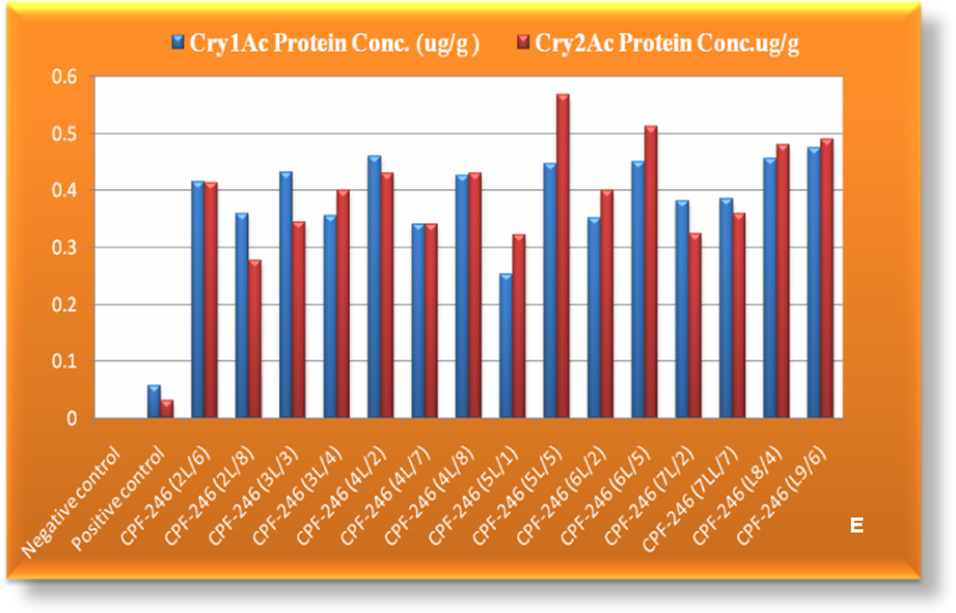


Figure 11: Based on ELISA quantification, comparisonbetween proteins of CEMB-*Cry*1Ac and CEMB-*Cry*2A of transgenic sugarcane plants in V_0_-generation

Lane 1: Control sugarcane plants Lane 2: Positive control Lane 3-17: Sugarcane transgenic plants


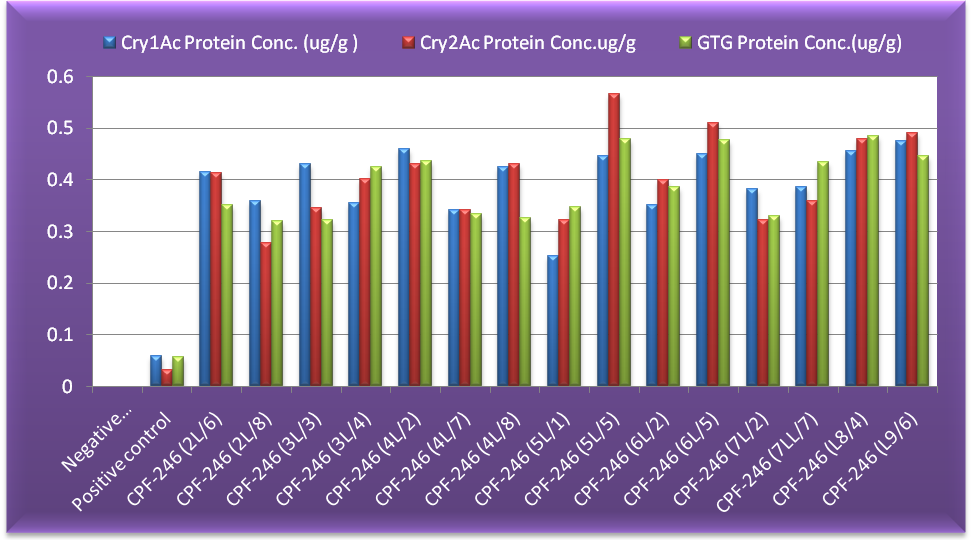


Figure 12: Based on ELISA quantification, comparison between proteins of CEMB-*Cry*1Ac CEMB-*Cry*2A and CEMB-GTGene of transgenic sugarcane plants in V_0_-generation

Lane 1: Control sugarcane plants Lane 2: Positive control Lane 3-17: Sugarcane transgenic plants


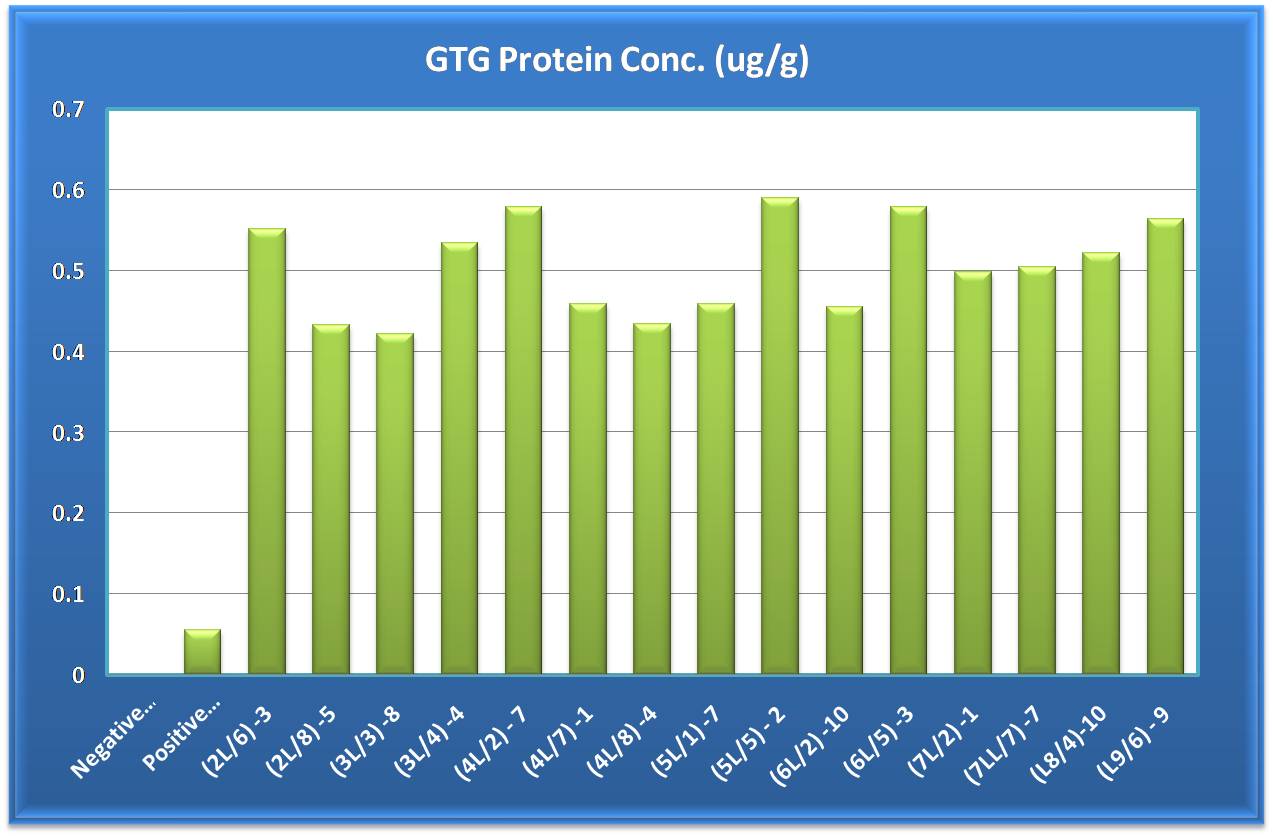


Figure 13: Randomly selected transgenic sugarcane CEMB-GTGene field plants in V_1_-generation.

Lane 1: Control sugarcane plants, Lane 2: Positive control, Lane 3-17: Sugarcane transgenic plants


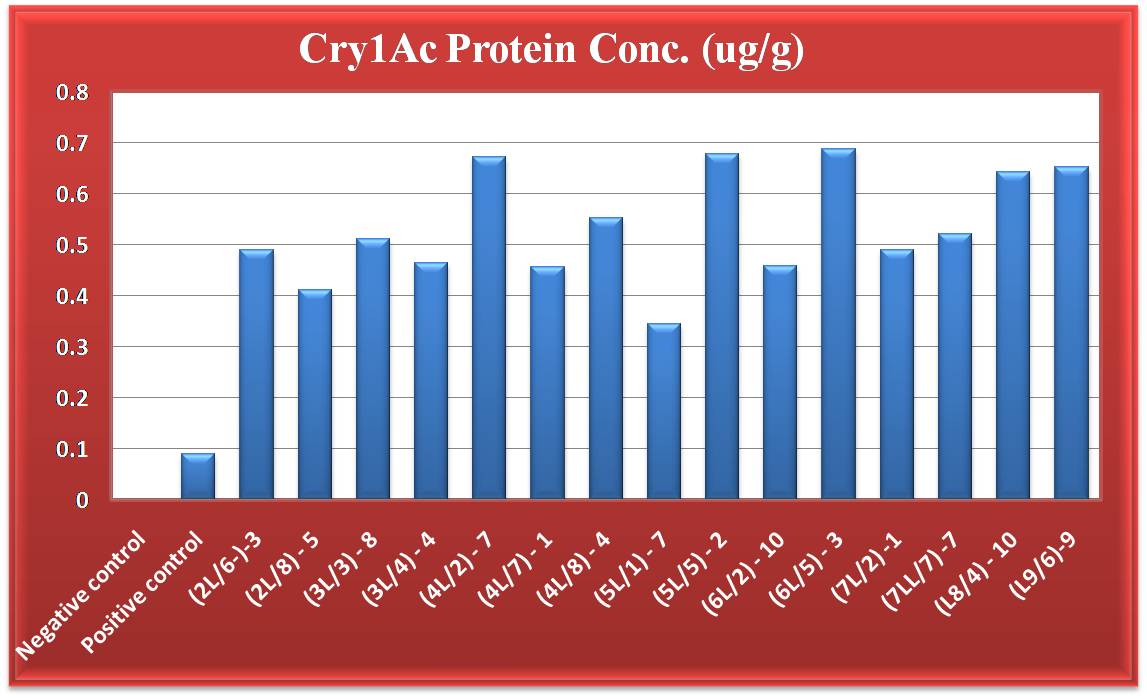


Figure 14: Randomly selected transgenic sugarcane CEMB-*Cry*1Ac field plants in V_1_-generation

Lane 1: Control sugarcane plants, Lane 2: Positive control, Lane 3-17: Sugarcane transgenic plants


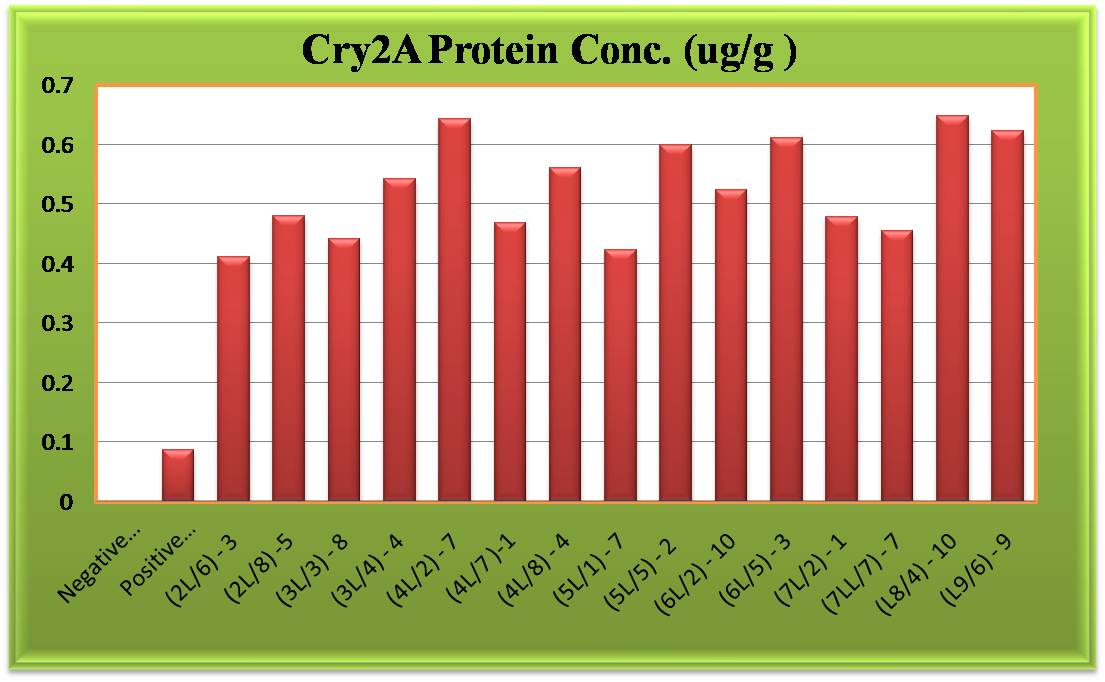


**Figure 15:** Randomly selected transgenic sugarcane CEMB-*Cry*2A field plants in V_1_-generation

Lane 1: Control sugarcane plants Lane 2: Positive control Lane 3-17: Sugarcane transgenic plants


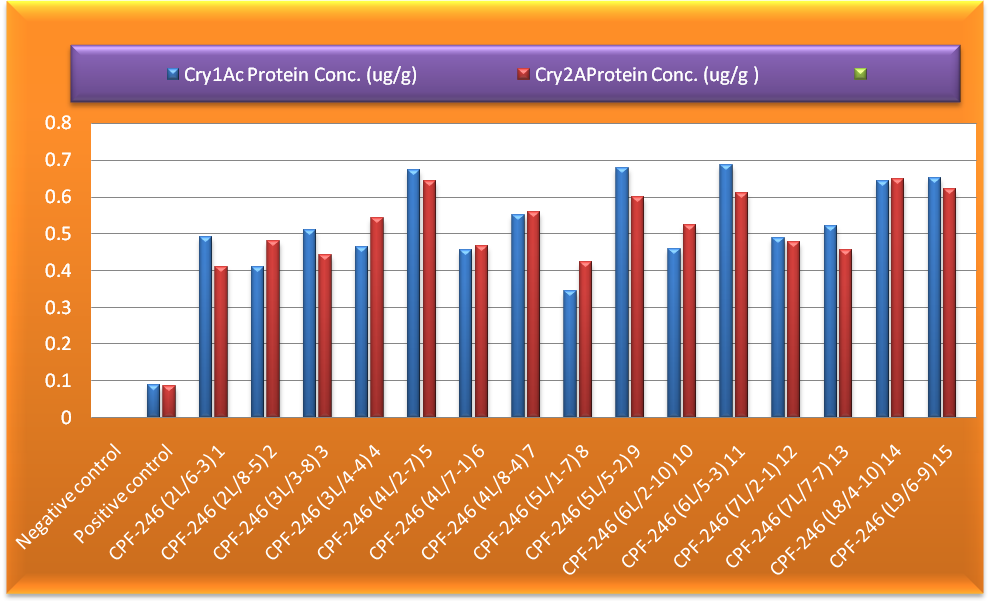


Figure 16: Comparison between expressions of CEMB-*Cry*1Ac and CEMB-*Cry*2A transgenes in transgenic sugarcane plants of V_1_-generation

Lane 1: Control sugarcane plants , Lane 2: Positive control, Lane 3-17: Sugarcane transgenic plants


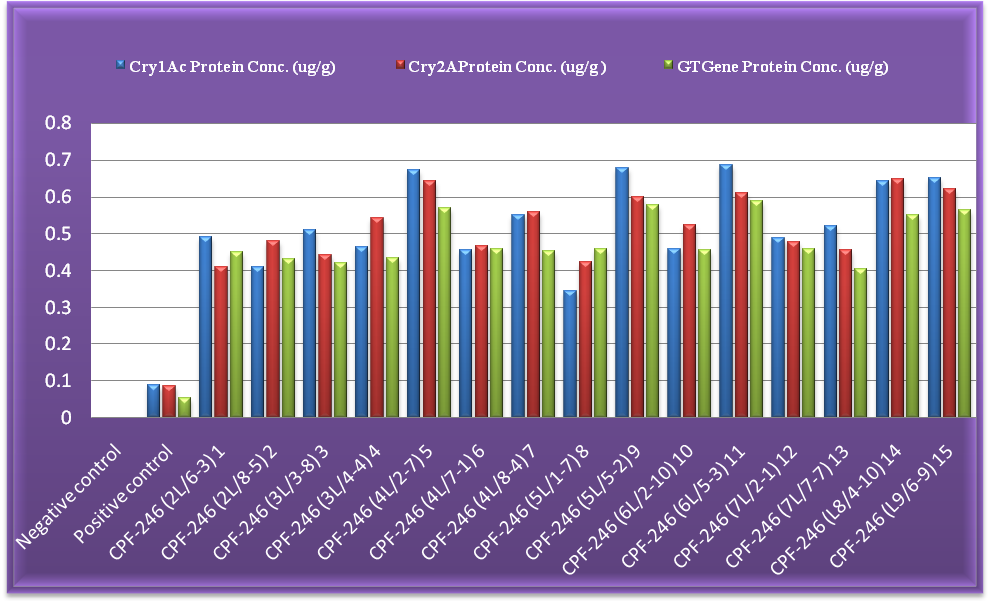


Figure 17: Comparison between expressions of CEMB-*Cry*1Ac, CEMB-*Cry*2A and CEMB-GTGene transgenes in transgenic sugarcane plants of V_1_-generation

Lane 1: Control sugarcane plants Lane 2: Positive control Lane 3-17: Sugarcane transgenic plants

Table 8: Glyphosate-herbicide spray assay of transgenic sugarcane plants in

V_1_-generation

| **Serial No** | **Transgenic Lines (CPF-246)** | **Percentage of Glyphosate Tolerant Transgenic plants (1200mL/acre)** |
| --- | --- | --- |
| **1** | **Control** | 0 % |
| **2** | CPF-246 (2L/6) | 70 % Tolerant plants & 30 % Died |
| **3** | CPF-246 (2L/8) | 70 % Tolerant plants & 30 % Died |
| **4** | CPF-246 (3L/3) | 70 % Tolerant plants & 30 % Died |
| **5** | CPF-246 (3L/4) | 70 % Tolerant plants & 30 % Died |
| **6** | CPF-246 (4L/2) | 75 % Tolerant plants & 25 % Died |
| **7** | CPF-246 (4L/7) | 71 % Tolerant plants & 29 % Died |
| **8** | CPF-246 (4L/8) | 70 % Tolerant plants & 30 % Died |
| **9** | CPF-246 (5L/1) | 71 % Tolerant plants & 29 % Died |
| **10** | CPF-246 (5L/5) | 75 % Tolerant plants & 25% Died |
| **11** | CPF-246 (6L/2) | 71 % Tolerant plants & 29 % Died |
| **12** | CPF-246 (6L/5) | 76 % Tolerant plants & 24 % Died |
| **13** | CPF-246 (7L/2) | 70% Tolerant plants & 30% Died |
| **14** | CPF-246 (7LL/7) | 7 0% Tolerant plants & 30% Died |
| **15** | CPF-246 (L8/4) | 75 % Tolerant plants & 25 % Died |
| **16** | CPF-246 (L9/6) | 75 % Tolerant plants & 25 % Died |

Table 9: Leaf Bio-toxicity assay of transgenic sugarcane plants from V1-generation

| Sugarcane Plants  (CPF-246) | | Leaf Bio-assay, Mortality percentage of *Chilo infuscatellus* Larvae at different ages of transgenic plants (days) | | | |
| --- | --- | --- | --- | --- | --- |
|  |  | 20 days  (%age) | 40 days  (%age) | 60 days  (%age) | 80 days  (%age) |
| Non-transgenic sugarcane plants | 1-10 | 0 | 0 | 0 | 0 |
| CPF-246 (2L/6)  CPF-246 (2L/6)  CPF-246 (2L/6)  CPF-246 (2L/6)  CPF-246 (2L/6)  CPF-246 (4L/2)  CPF-246 (2L/6)  CPF-246 (2L/6)  CPF-246 (2L/6)  CPF-246 (2L/6) | 1  2  3  4  5  6  7  8  9  10 | 80  80  70  70  70  70  80  70  80  70 | 80  80  80  80  70  70  80  70  80  70 | 80  80  80  80  80  70  80  80  80  70 | 80  90  80  90  80  70  80  80  80  70 |
| CPF-246 (2L/8)  CPF-246 (2L/8)  CPF-246 (2L/8)  CPF-246 (2L/8)  CPF-246 (2L/8)  CPF-246 (2L/8)  CPF-246 (2L/8)  CPF-246 (2L/8)  CPF-246 (2L/8)  CPF-246 (2L/8) | 1  2  **3**  4  **5**  6  7  8  9  10 | 80  80  80  70  80  70  80  80  70  80 | 80  80  80  70  80  80  80  80  80  80 | 80  80  80  90  80  90  80  80  80  80 | 80  80  80  90  80  90  90  80  80  90 |
| CPF-246 (3L/3)  CPF-246 (3L/3)  CPF-246 (3L/3)  CPF-246 (3L/3)  CPF-246 (3L/3)  CPF-246 (3L/3)  CPF-246 (3L/3)  CPF-246 (3L/3)  CPF-246 (3L/3)  CPF-246 (3L/3) | **1**  **2**  **3**  **4**  **5**  **6**  **7**  8  **9**  **10** | 70  60  70  80  70  70  60  80  70  70 | 70  60  70  80  70  80  70  80  70  70 | 80  70  80  80  70  80  70  80  70  80 | 80  70  80  80  70  90  70  80  70  80 |
| CPF-246 (3L/4)  CPF-246 (3L/4)  CPF-246 (3L/4)  CPF-246 (3L/4)  CPF-246 (3L/4)  CPF-246 (3L/4)  CPF-246 (3L/4)  CPF-246 (3L/4)  CPF-246 (3L/4)  CPF-246 (3L/4) | 1  2  **3**  4  **5**  6  7  8  9  10 | 70  70  70  80  70  70  70  70  80  60 | 70  70  70  80  70  80  70  80  80  60 | 70  80  70  80  70  80  70  80  80  60 | 70  80  70  80  70  90  70  80  80  70 |
| CPF-246 (4L/2)  CPF-246 (4L/2)  CPF-246 (4L/2)  CPF-246 (4L/2)  CPF-246 (4L/2)  CPF-246 (4L/2)  CPF-246 (4L/2)  CPF-246 (4L/2)  CPF-246 (4L/2)  CPF-246 (4L/2) | 1  2  **3**  4  **5**  6  7  8  9  10 | 80  80  80  80  80  70  80  80  100  80 | 90  90  80  80  80  70  80  80  100  80 | 90  90  80  80  80  70  80  90  100  80 | 90  90  90  90  80  80  80  90  100  80 |
| CPF-246 (4L/7)  CPF-246 (4L/7)  CPF-246 (4L/7)  CPF-246 (4L/7)  CPF-246 (4L/7)  CPF-246 (4L/7)  CPF-246 (4L/7)  CPF-246 (4L/7)  CPF-246 (4L/7)  CPF-246 (4L/7) | 1  2  **3**  4  **5**  6  7  8  9  10 | 80  70  70  70  70  70  60  70  70  60 | 80  70  80  70  70  70  60  70  70  60 | 80  70  80  70  80  70  60  80  70  70 | 80  70  80  70  80  80  60  80  80  70 |
| CPF-246 (4L/8)  CPF-246 (4L/8)  CPF-246 (4L/8)  CPF-246 (4L/8)  CPF-246 (4L/8)  CPF-246 (4L/8)  CPF-246 (4L/8)  CPF-246 (4L/8)  CPF-246 (4L/8)  CPF-246 (4L/8) | 1  2  **3**  4  **5**  6  7  8  9  10 | 80  70  70  80  80  60  60  70  70  60 | 80  80  70  80  80  60  60  70  70  70 | 80  80  70  80  80  60  70  70  80  70 | 80  80  70  80  80  60  70  80  80  70 |
| CPF-246 (5L/1)  CPF-246 (5L/1)  CPF-246 (5L/1)  CPF-246 (5L/1)  CPF-246 (5L/1)  CPF-246 (5L/1)  CPF-246 (5L/1)  CPF-246 (5L/1)  CPF-246 (5L/1)  CPF-246 (5L/1) | 1  2  **3**  4  **5**  6  7  8  9  10 | 70  70  70  60  70  60  60  70  60  70 | 70  70  70  60  70  60  80  70  60  70 | 70  70  80  60  70  60  80  70  60  70 | 70  80  80  60  70  60  80  70  70  70 |
| CPF-246 (5L/5)  CPF-246 (5L/5)  CPF-246 (5L/5)  CPF-246 (5L/5)  CPF-246 (5L/5)  CPF-246 (5L/5)  CPF-246 (5L/5)  CPF-246 (5L/5)  CPF-246 (5L/5)  CPF-246 (5L/5) | 1  2  **3**  4  **5**  6  7  8  9  10 | 80  90  80  90  80  80  80  100  70  80 | 80  90  80  90  80  80  80  100  80  80 | 80  90  90  90  80  90  80  100  80  80 | 80  90  90  90  90  90  80  100  90  80 |
| CPF-246 (6L/2)  CPF-246 (6L/2)  CPF-246 (6L/2)  CPF-246 (6L/2)  CPF-246 (6L/2)  CPF-246 (6L/2)  CPF-246 (6L/2)  CPF-246 (6L/2)  CPF-246 (6L/2)  CPF-246 (6L/2) | 1  2  **3**  4  **5**  6  7  8  9  10 | 70  60  80  60  60  60  70  60  70  80 | 70  70  80  70  70  60  70  70  70  80 | 70  70  80  70  70  80  70  80  70  80 | 70  70  80  70  70  80  70  80  90  80 |
| CPF-246 (6L/5)  CPF-246 (6L/5)  CPF-246 (6L/5)  CPF-246 (6L/5)  CPF-246 (6L/5)  CPF-246 (6L/5)  CPF-246 (6L/5)  CPF-246 (6L/5)  CPF-246 (6L/5)  CPF-246 (6L/5) | 1  2  **3**  4  **5**  6  7  8  9  10 | 90  90  90  100  90  70  80  100  80  80 | 90  90  90  100  90  70  80  100  90  80 | 90  90  90  100  90  80  90  100  90  90 | 90  100  90  100  90  80  90  100  90  90 |
| CPF-246 (7L/2)  CPF-246 (7L/2)  CPF-246 (7L/2)  CPF-246 (7L/2)  CPF-246 (7L/2)  CPF-246 (7L/2)  CPF-246 (7L/2)  CPF-246 (7L/2)  CPF-246 (7L/2)  CPF-246 (7L/2) | 1  2  **3**  4  **5**  6  7  8  9  10 | 70  60  60  70  60  60  60  70  70  60 | 70  70  70  70  70  60  70  70  70  60 | 70  70  70  80  70  70  70  70  70  70 | 70  70  80  80  70  70  70  70  70  70 |
| CPF-246 (7L/7)  CPF-246 (7L/7)  CPF-246 (7L/7)  CPF-246 (7L/7)  CPF-246 (7L/7)  CPF-246 (7L/7)  CPF-246 (7L/7)  CPF-246 (7L/7)  CPF-246 (7L/7)  CPF-246 (7L/7) | 1  2  **3**  4  **5**  6  7  8  9  10 | 70  60  60  70  60  70  80  60  70  60 | 70  60  60  70  70  70  80  60  70  70 | 70  60  60  70  80  70  80  70  70  70 | 70  60  60  70  80  70  80  70  80  70 |
| CPF-246 (L8/4)  CPF-246 (L8/4)  CPF-246 (L8/4)  CPF-246 (L8/4)  CPF-246 (L8/4)  CPF-246 (L8/4)  CPF-246 (L8/4)  CPF-246 (L8/4)  CPF-246 (L8/4)  CPF-246 (L8/4) | 1  2  **3**  4  **5**  6  7  8  9  10 | 80  80  90  80  80  80  80  80  80  90 | 90  80  90  80  80  80  80  90  90  90 | 90  90  100  90  90  90  80  90  80  80 | 90  90  100  100  90  90  80  90  80  90 |
| CPF-246 (L9/6)  CPF-246 (L9/6)  CPF-246 (6L/5)  CPF-246 (L9/6)  CPF-246 (L9/6)  CPF-246 (L9/6)  CPF-246 (L9/6)  CPF-246 (L9/6)  CPF-246 (L9/6)  CPF-246 (L9/6) | 1  2  **3**  4  **5**  6  7  8  9  10 | 80  90  80  80  90  80  90  80  90  80 | 80  90  80  80  90  80  90  80  90  90 | 90  90  80  80  90  80  90  80  90  100 | 90  90  90  100  100  80  90  90  90  100 |

Note: Only those sugarcane transgenic lines were considered which were highly insect resistant and glyphosate spray tolerant (1200mL/80L/acre)

**Original Gel and Blot figures
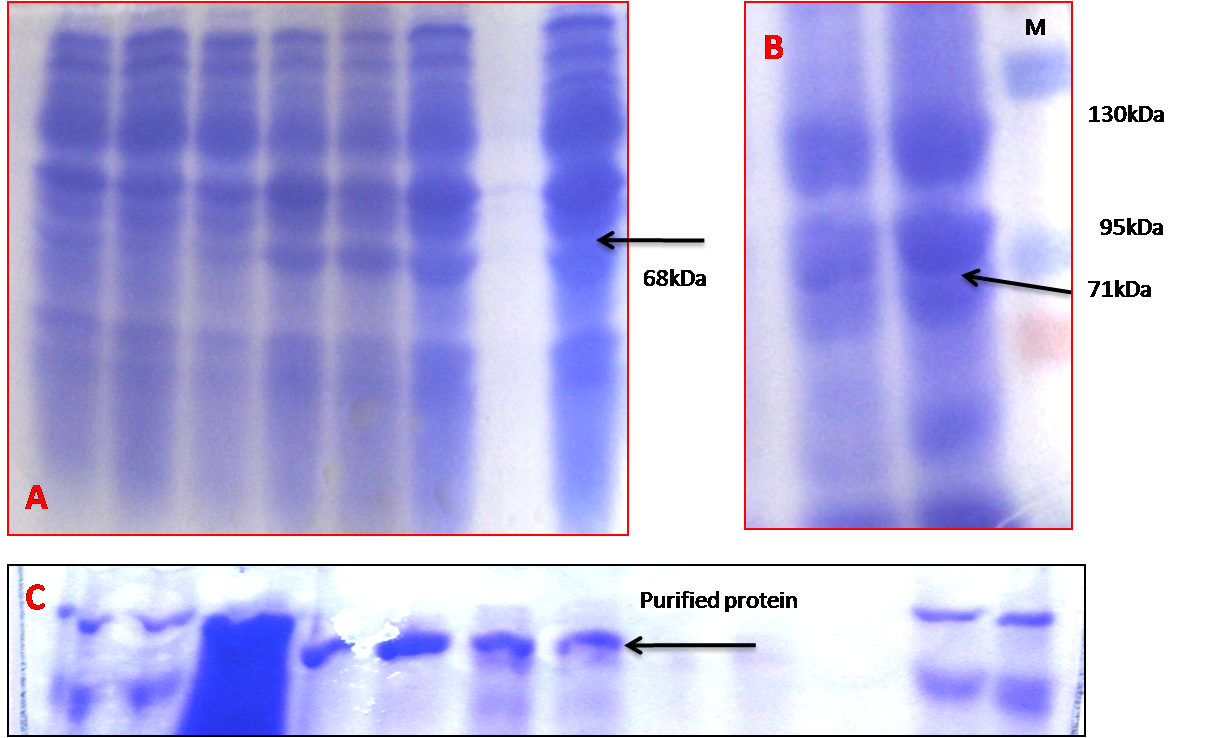

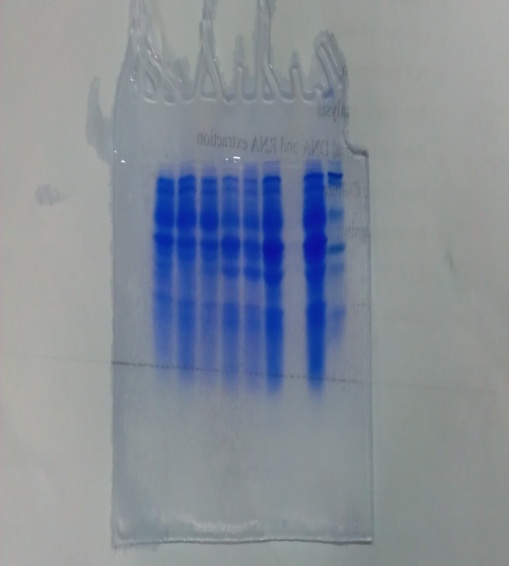

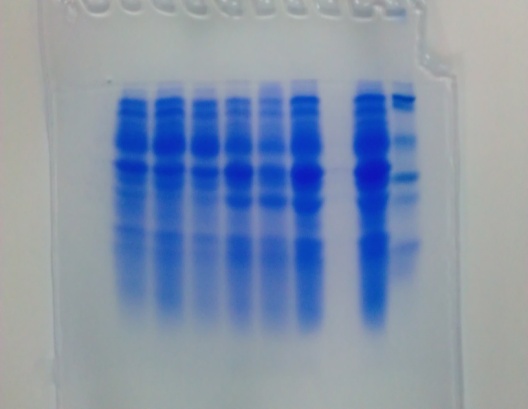

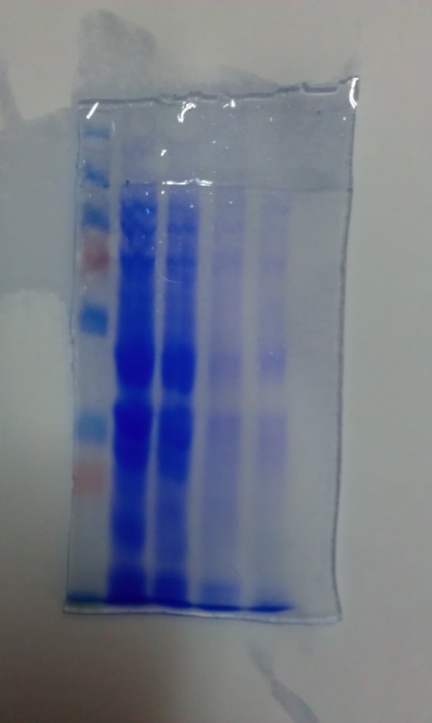
**

**
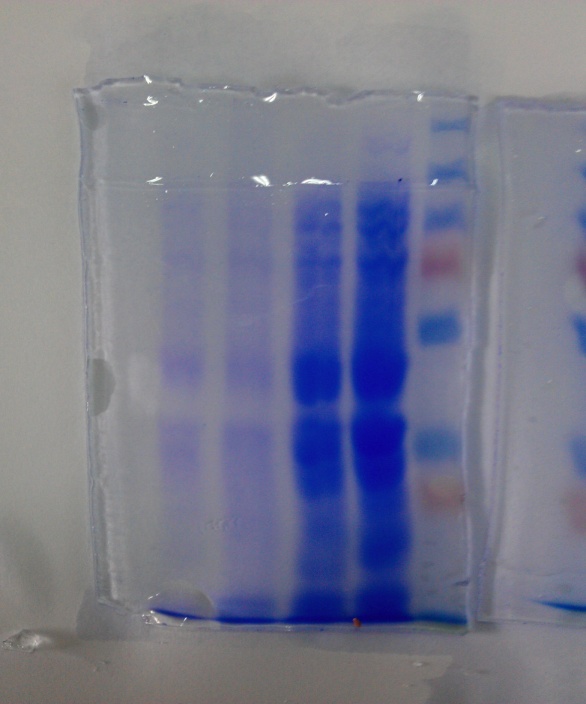

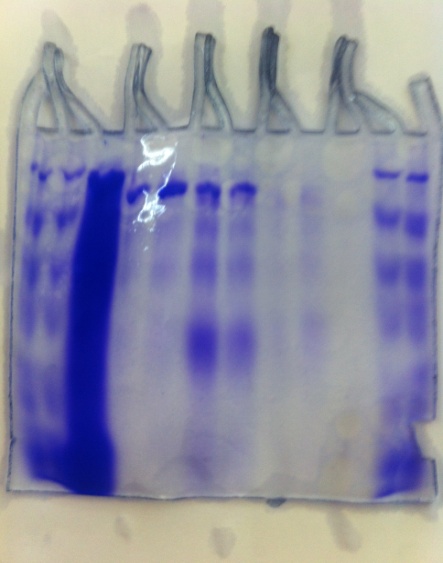
**

**
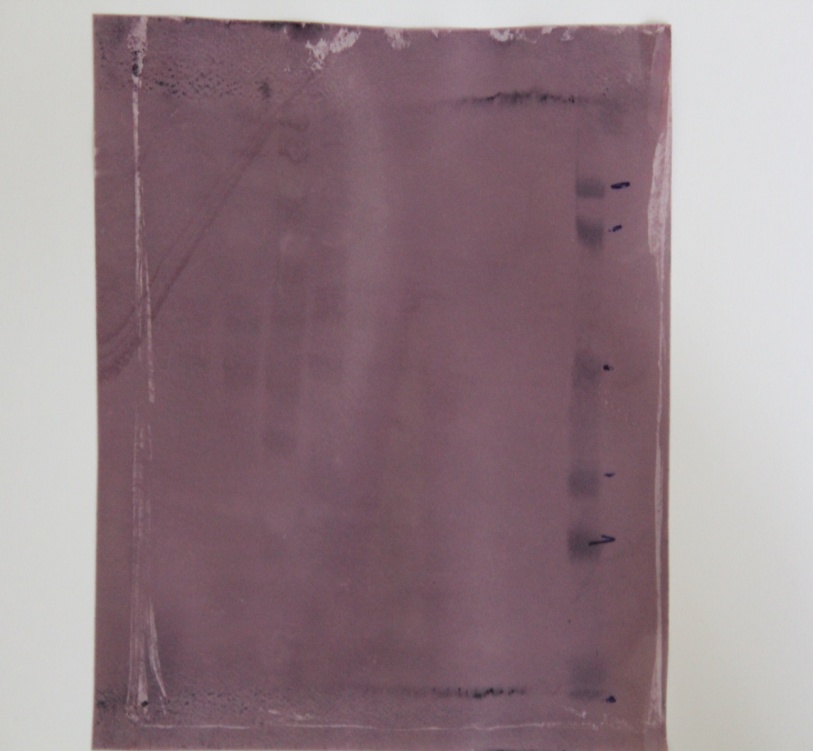
**

**
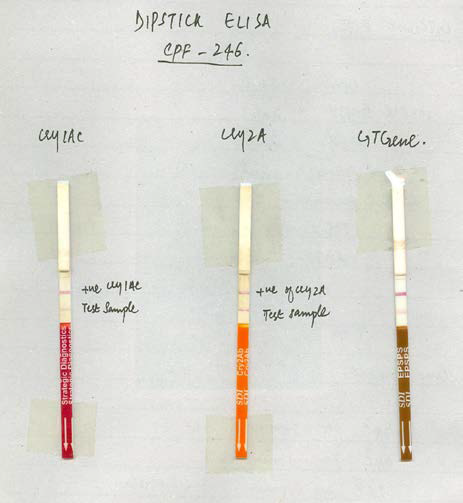
**

**
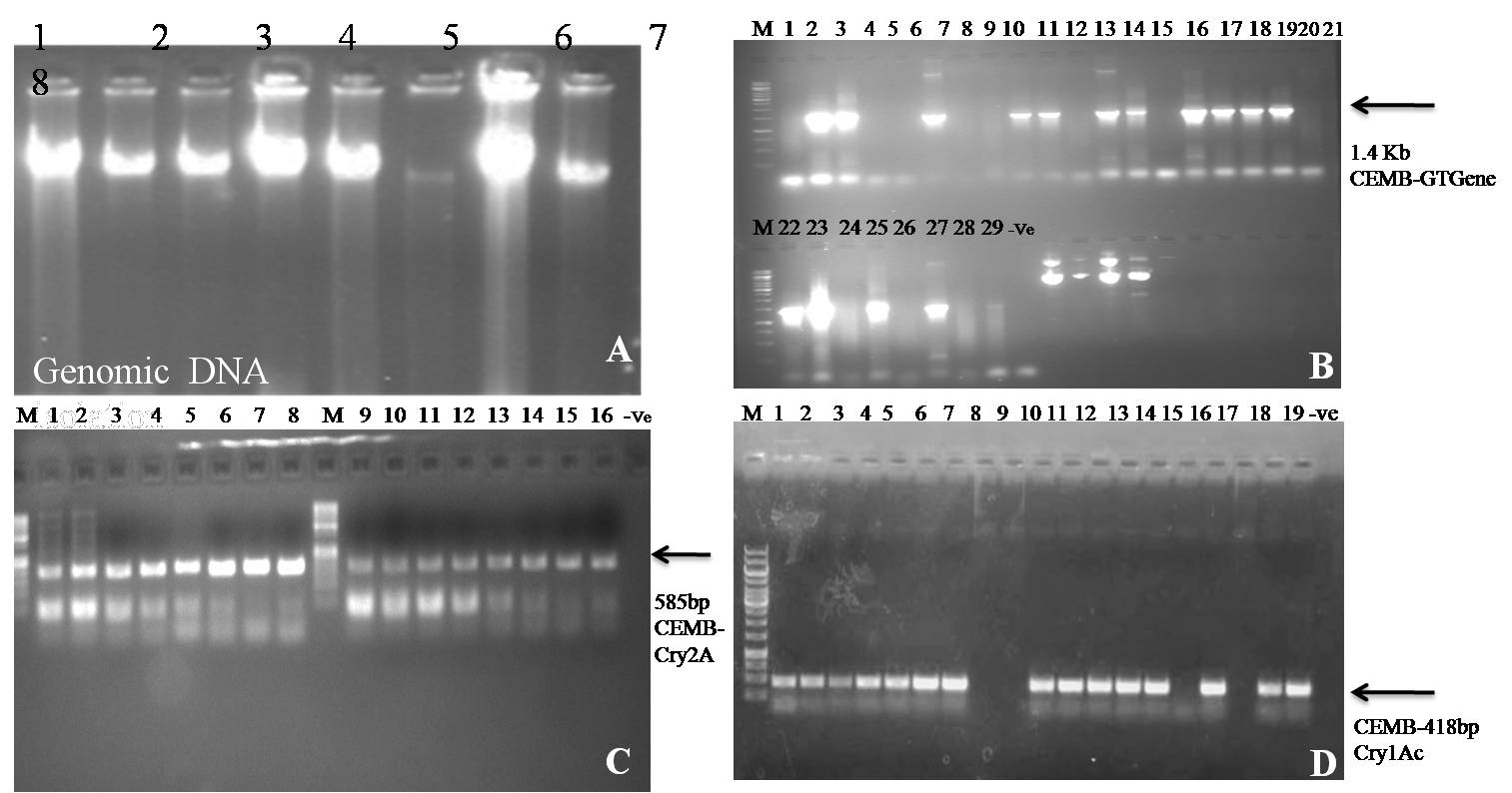

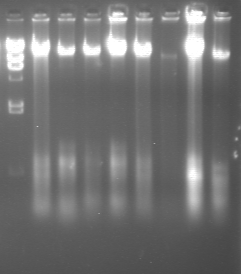

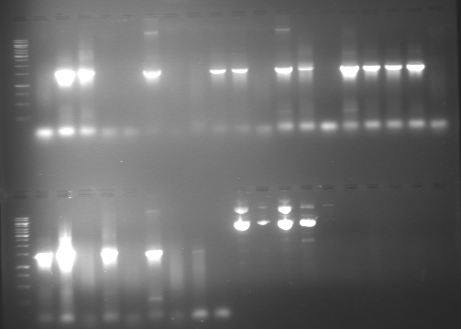

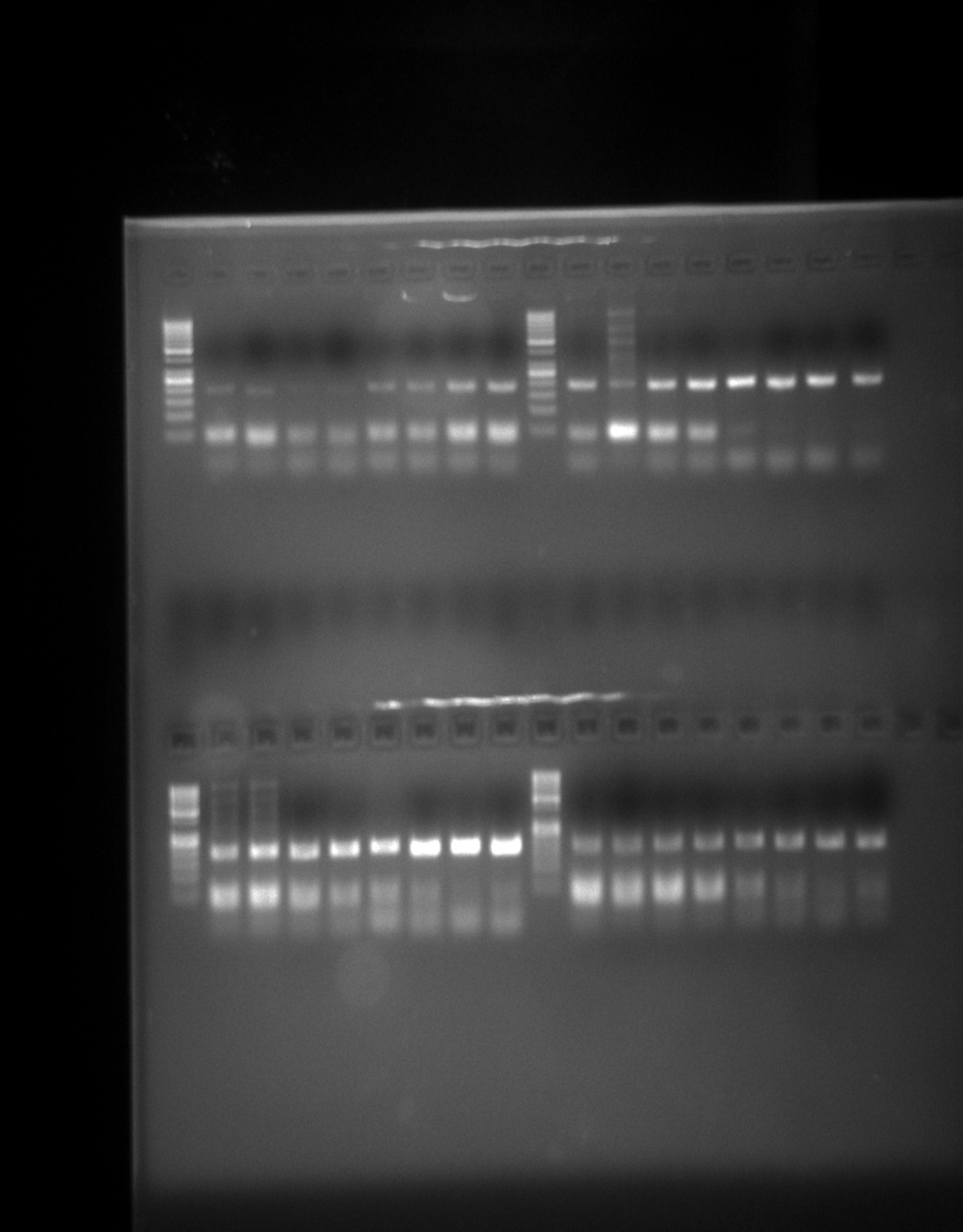
**

**
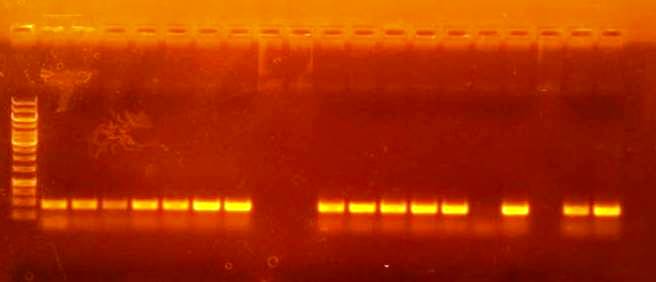
**

**
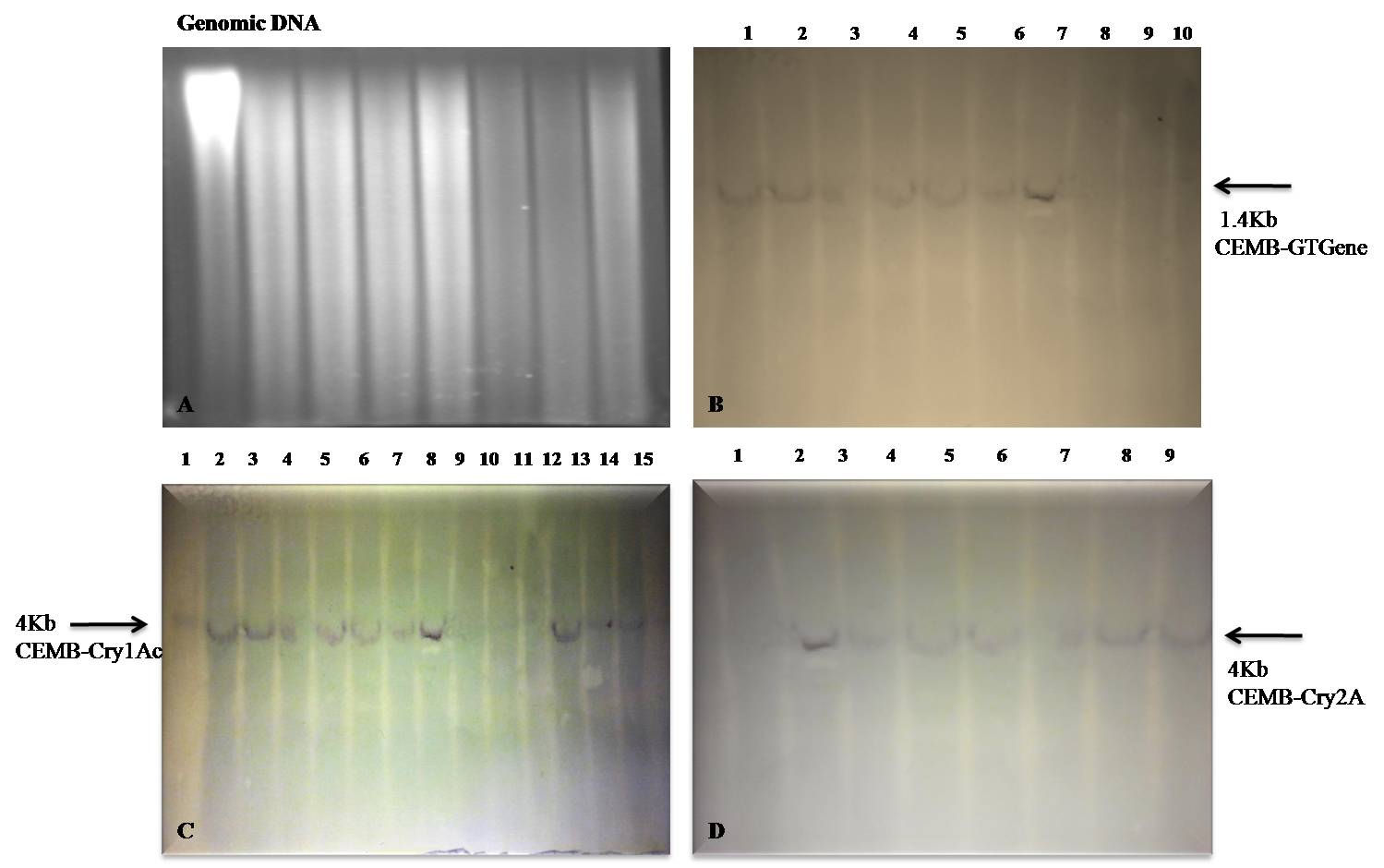

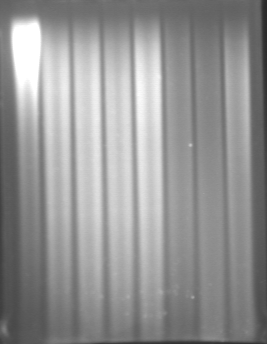

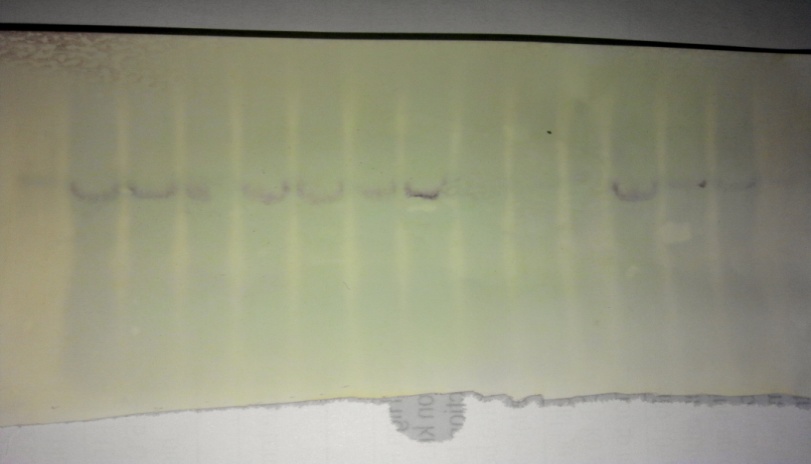

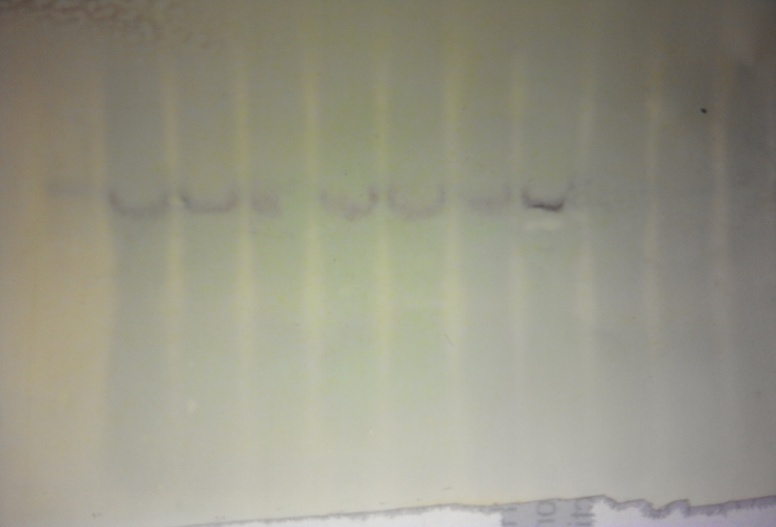
**

**
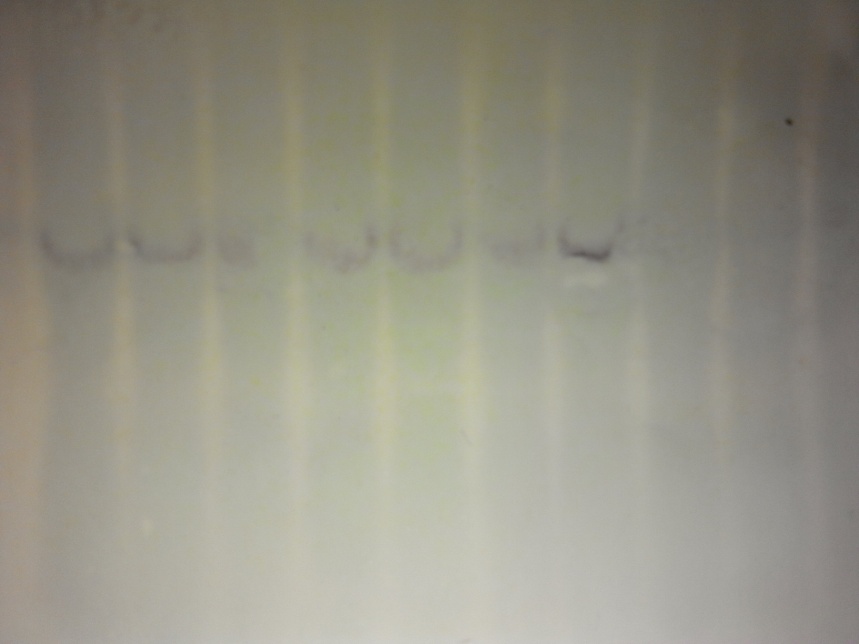
**

**
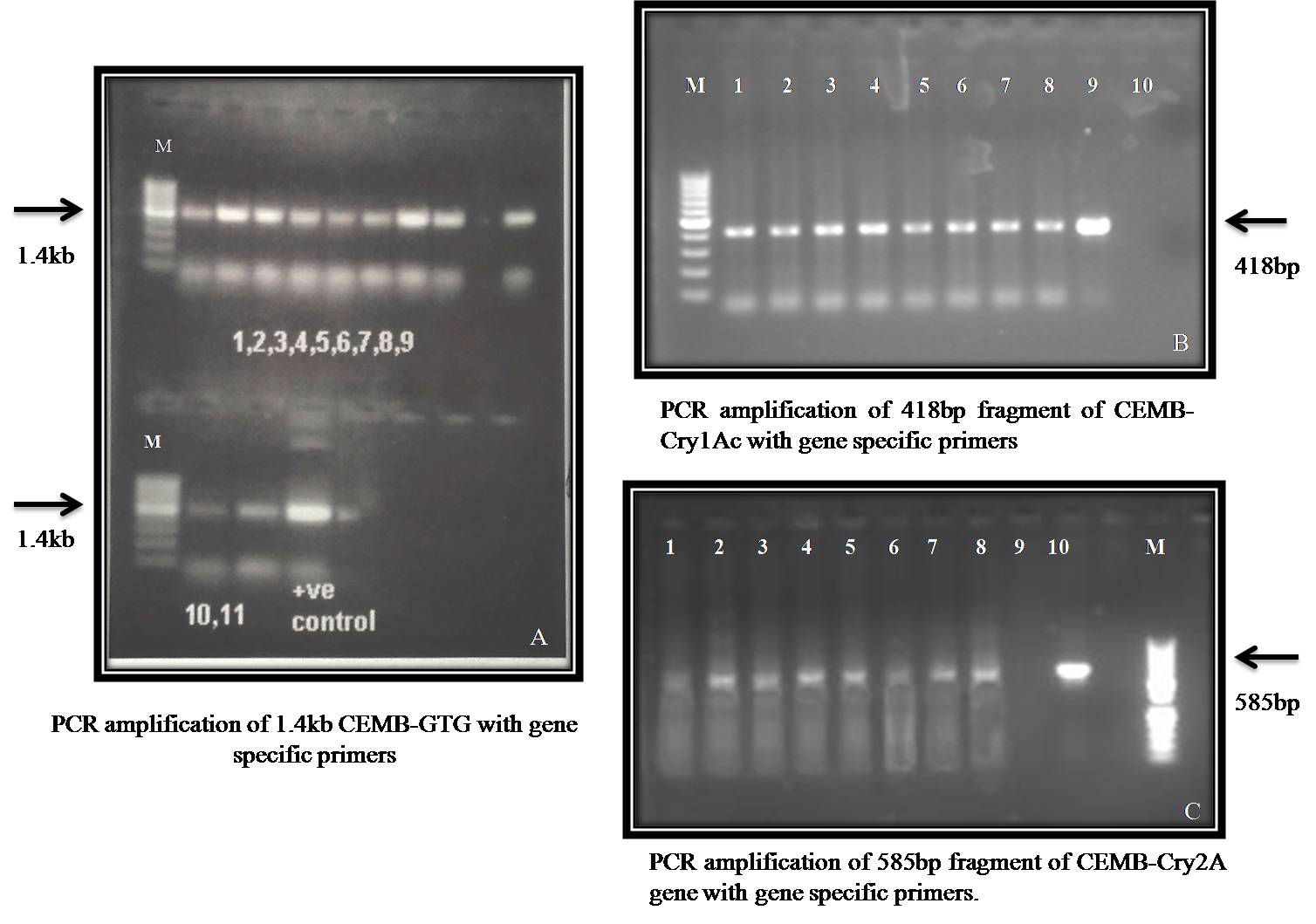

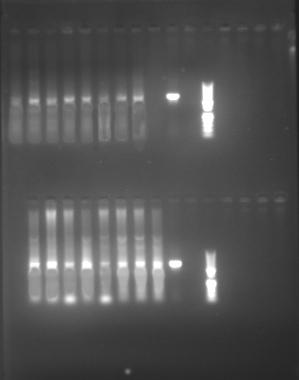

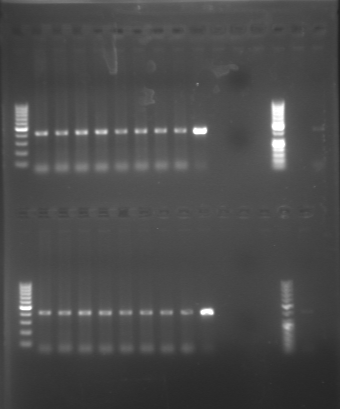
**

**
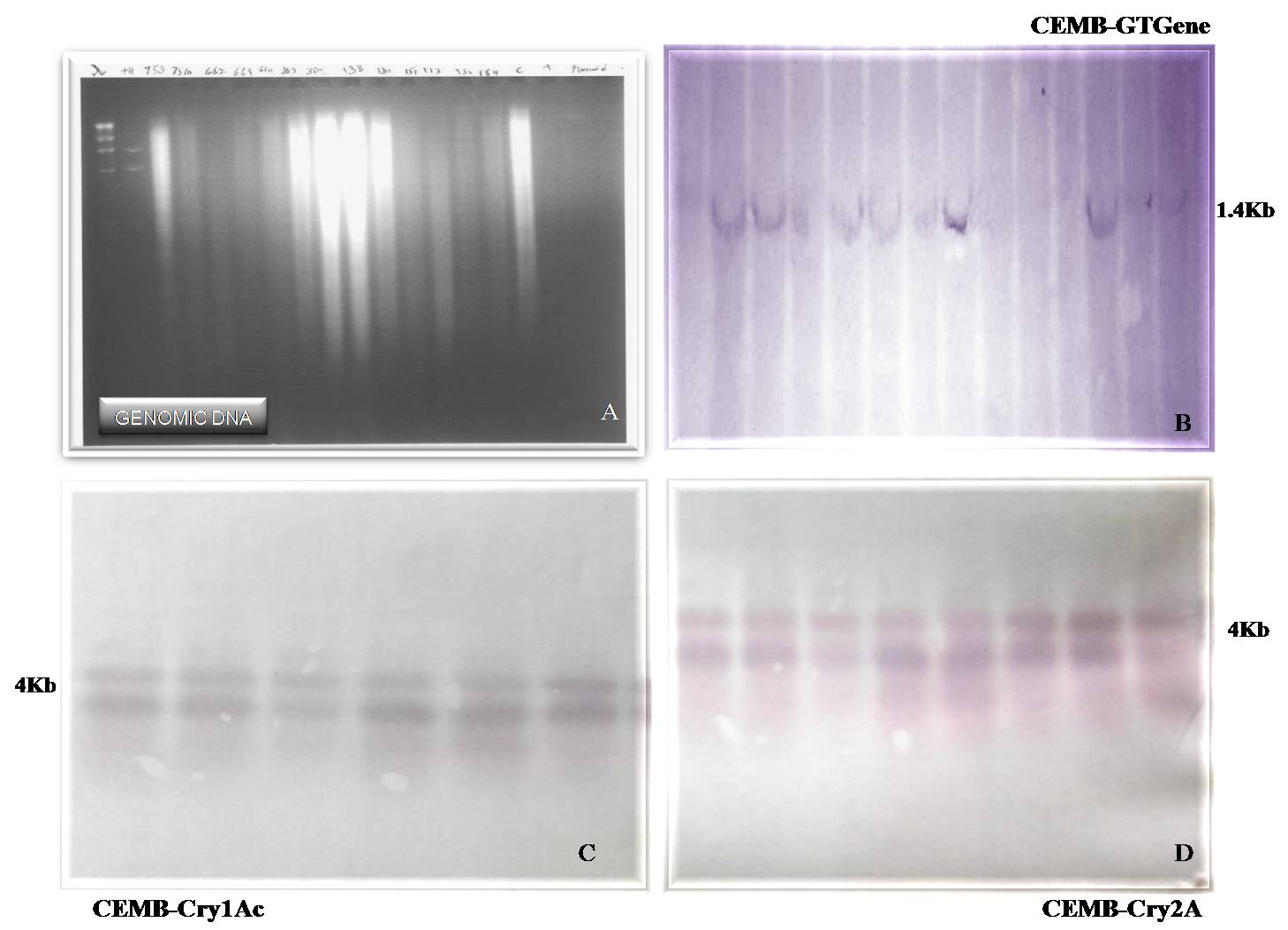

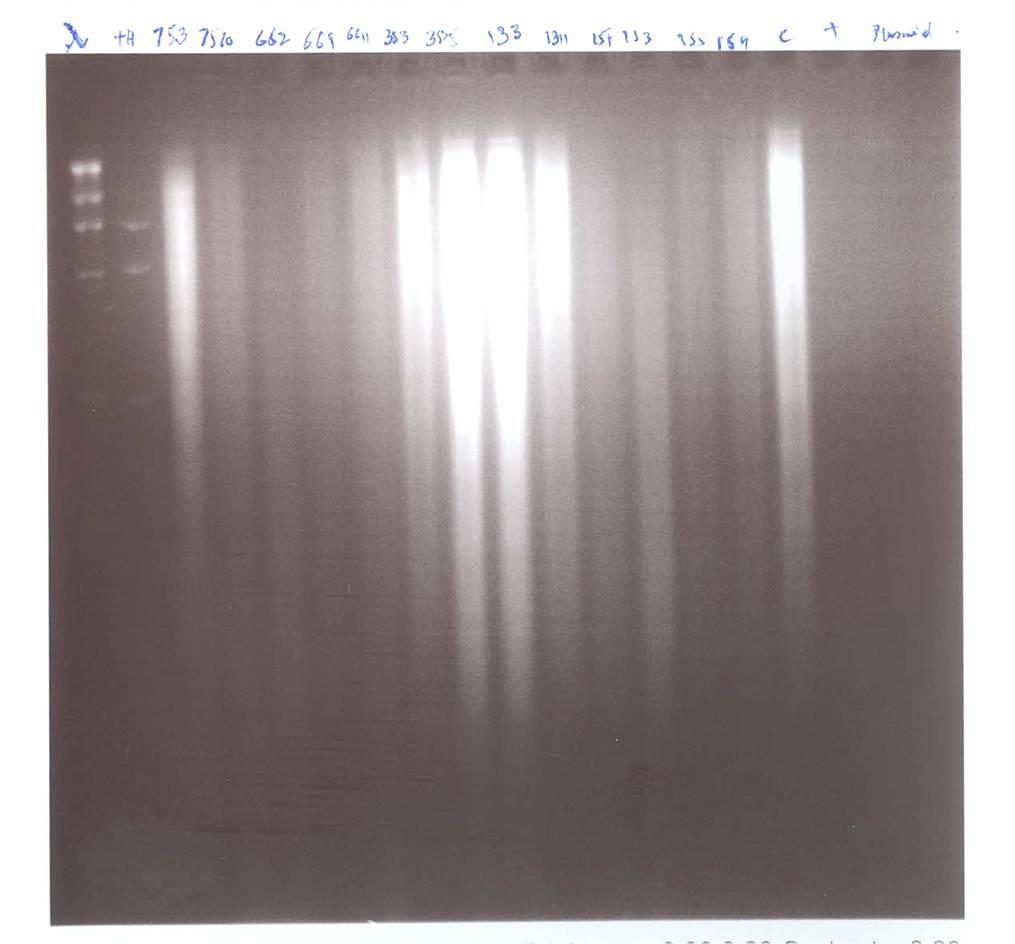
**
